# Solvent-Free Catalytic Hydrotreatment of Lignin to Biobased Aromatics: Current Trends, Industrial Approach, and Future Perspectives

**DOI:** 10.1021/acs.energyfuels.4c05174

**Published:** 2024-12-17

**Authors:** Ramesh Kumar Chowdari, Parameswaram Ganji, Blaž Likozar

**Affiliations:** † Institute of Chemistry, University of Graz, Heinrichstrasse 28/II, 8010 Graz, Styria, Austria; ‡ Department of Catalysis and Chemical Reaction Engineering, National Institute of Chemistry, Hajdrihova Ulica 19, 1001 Ljubljana, Slovenia; § Jozef Stefan Institute, Department of Surface Engineering, Jamova Cesta 39, 1000 Ljubljana, Slovenia

## Abstract

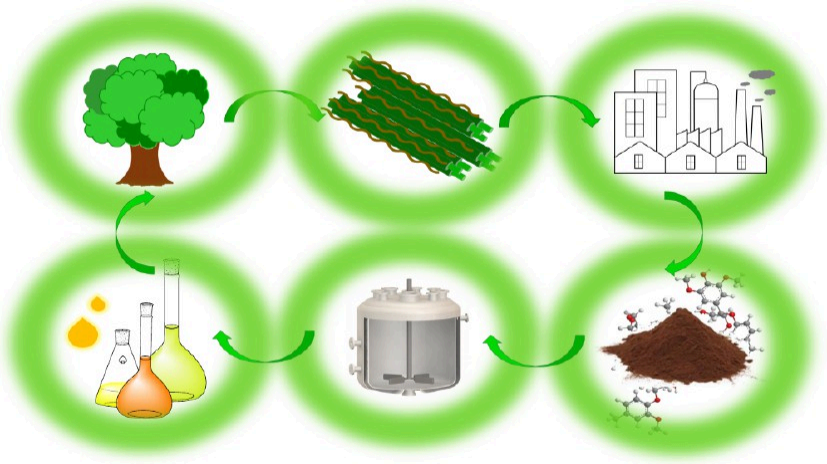

Lignin is the only naturally occurring, renewable biopolymer and an alternative for the production of six-membered aromatic chemicals. The utilization of lignin can increase the additional revenue of biorefineries and reduce the dependence on crude oil for the production of aromatic chemicals. Therefore, the development of technologies for the production of valuable chemicals from lignin waste in biorefineries is of great importance. Catalytic hydrotreatment of lignin is considered one of the most promising technologies for the production of biobased aromatic chemicals and fuels. Among the various hydrotreatment routes, the solvent-free hydrotreatment approach is advantageous because this process reduces production costs and is similar to petroleum refinery processes such as cracking and heteroatom removal. This review addresses recent developments in solvent-free catalytic hydrotreatment of various lignins such as sulfur-containing, sulfur-free, and pyrolytic lignins to produce low oxygen-containing aromatics such as alkylphenolics in batch, semicontinuous, and continuous reactors. Special emphasis is given to the various noble and non-noble metal catalysts, the best route between single and two-stage processing, key factors in solvent-free depolymerization of lignin, techno-economic evaluation, crude oil vs lignin oil refining, challenges and future prospects, etc.

## 1 Introduction

Crude oil has become a major source of aromatic chemicals and fuels since its discovery. Energy demand continues to increase due to the growing population; currently, 85% of energy demand is met by fossil fuels.^1^ It is expected that by 2030, 27% of the EU’s total energy consumption and 20% of the U.S. energy demand will come from biorenewable resources.^2,3^ However, the production of fuels and petrochemical products such as aromatics (BTX) from fossil fuels is questionable due to “sustainability”. Therefore, the focus of research has shifted significantly to sustainable development for several decades.

Lignocellulosic biomass appears to be an important alternative to petrochemicals due to its sustainability and availability for the production of chemicals and biofuels. However, due to the high oxygen content in lignocellulosic biomass, the energy density is relatively lower than that of fossil.^4^ Lignocellulosic biomass is the main residue from forests and crops, in which three types of polymers are present, namely, cellulose (40–50%), hemicellulose (20–30%), lignin (15–25%), and a small amount of pectin and protein.^5^ The content of these biopolymers depends on the origin of the wood species and age. The main fragment in cellulose and hemicellulose is the C_5_–C_6_ sugar unit. The lignin polymer, on the other hand, consists of aromatic structures and provides structural integrity to the cell wall. The carbon content in lignin is relatively high (64.2%) compared to cellulose and hemicellulose.^6^ Therefore, the extraction/isolation and conversion of these naturally occurring polymers produces sustainable fine chemicals and biofuels. The first generation of biofuel is ethanol derived from cellulose and hemicellulose.^7,8^ In the biorefinery about 0.5–1.5 kg of lignin is produced for each liter of ethanol.^9^ The biorefinery, pulp and paper industries produce about 130 million tons of lignin annually, which is generally used as fuel to generate heat for boilers.^10^ Therefore, the future biomass refinery should aim at producing no waste, i.e., using the abundant lignin. In this context, the utilization of all wood biomass, including lignin, into valuable chemicals would provide additional revenue to biorefinery companies. The literature reports that 98% of the lignin produced in the industry is burnt as fuel to produce internal energy for the industry, and only 2% is used commercially to produce valuable products.^11^

In addition to energy production, there are other opportunities such as the production of syngas, fuels, and chemicals, etc.^12^ Since lignin is the only naturally occurring biopolymer consisting of a six-membered aromatic structure with relatively high carbon and low oxygen content compared to other biopolymers such as cellulose and hemicellulose. Therefore, lignin is extremely interesting and can be converted into renewable chemical building blocks and fuels. However, the complexity, the degree of polymerization, and the various functional groups connecting the six-membered aromatic ring render lignin recalcitrant.^13^ Two main bonds are observed in the lignin polymer, namely ether (C–O–C) and C–C. In the depolymerization of lignin, the cleavage of ether bonds is easier than that of C–C bonds. Therefore, the challenges involve in lignin depolymerization are product yield, selectivity and efficiency.

In the past decade, enormous efforts have been made to develop effective techniques for the depolymerization of lignin and several review articles have also appeared in the literature.^14−22^ These include pyrolysis, supercritical solvents, enzyme/photo/electro-catalysis, hydrogenation, oxidation, microwave assisted lignin depolymerization etc. Among the processes reported, catalytic conversion of lignin is one of the most efficient.^20^ The advantages of heterogeneous catalysis include minimization of waste generation, reusability, long last activity, reduction of process cost etc.^21,23^ The depolymerization of lignin is generally performed under harsh reaction conditions, as the lignin polymer is recalcitrant and the production of aromatics with low/no-oxygen content is a challenging task. Therefore, it is very important to choose the type of catalyst and the reaction conditions so that a low amount of char and a high yield of the desired product can be produced. Furthermore, selective cleavage of the bonds is very important to control the desired product yields. By understanding the properties of the different types of bond in lignin and the reaction mechanism for the desired product, the catalyst can be developed.

The main objective of this review article is to provide the knowledge and latest findings on the production of 6-membered aromatics from lignins, especially high energy content chemicals such as low/no oxygen aromatics (e.g., Alkylphenolics) using a solvent-free hydrotreatment technique. This is because aromatics are the building blocks for the production of chemicals and fuels/biofuels (e.g., gasoline). The production of aromatics from lignin is therefore an alternative to nonrenewable crude oil/petroleum products. However, since lignin polymers contain a high proportion of oxygen in the form of polyphenols, the production of high-value chemicals such as low-oxygen/oxygen-free aromatics from lignin involves hydrogenolysis (breaking of C–C and C–O bonds) and hydrodeoxygenation reactions.^24,25^ In this context, catalytic hydrotreatment of lignin is one of the most studied technologies for the production of low oxygen/oxygen-free aromatics.^24^ However, there are limited research reports available for the solvent-free hydrotreatment. Therefore, this review article focuses on the various heterogeneous catalytic systems for the hydrotreatment of lignin to aromatics such as alkyl phenolics and oxygen-free aromatics.

In this review article, we address the different strategies and advances in solvent -free and thermo-catalytic depolymerization of lignin for the production of chemicals and fuels over the past decade. In solvent-free catalytic hydrotreatment, no external organic/aqueous solvents are used^26−34^ i.e., the reaction mixture consists of the following components: Lignin or its derivatives, such as pyrolytic lignin or pyrolysis oil are mixed with the catalyst, and high hydrogen pressure is applied. The reaction is usually carried out at high temperatures (>300 °C) and high pressures (>50 bar). This review covers lignin structure, classification of lignin, and its production, lignin depolymerization routes, advantages and disadvantages of solvent-based/solvent-free techniques, catalytic depolymerization of lignin in batch, semicontinuous and continuous reactors, key factors in lignin hydrotreatment, techno-economic evaluation and future direction of lignin depolymerization/hydrotreatment etc. As already mentioned, there are many review articles in the literature on the conversion of lignin into value-added products. In the few review articles, solvent-free hydrotreatment is discussed as part of the various lignin depolymerization routes. However, none of the review articles emphasized the solvent-free hydrotreatment technique. This is because the solvent-free lignin hydrotreatment strategy is different from the solvent-based and other reported methods. In this context, this article is important to bridge the knowledge gap between the other lignin utilization techniques and the solvent-free technique. To the best of the author’s knowledge, this is the first full-length review article devoted to “solvent-free catalytic hydrotreatment of lignin”.

## 2 Lignin Structure, Types, and Production

### 2.1 Lignin Structure

**Figure 1:**
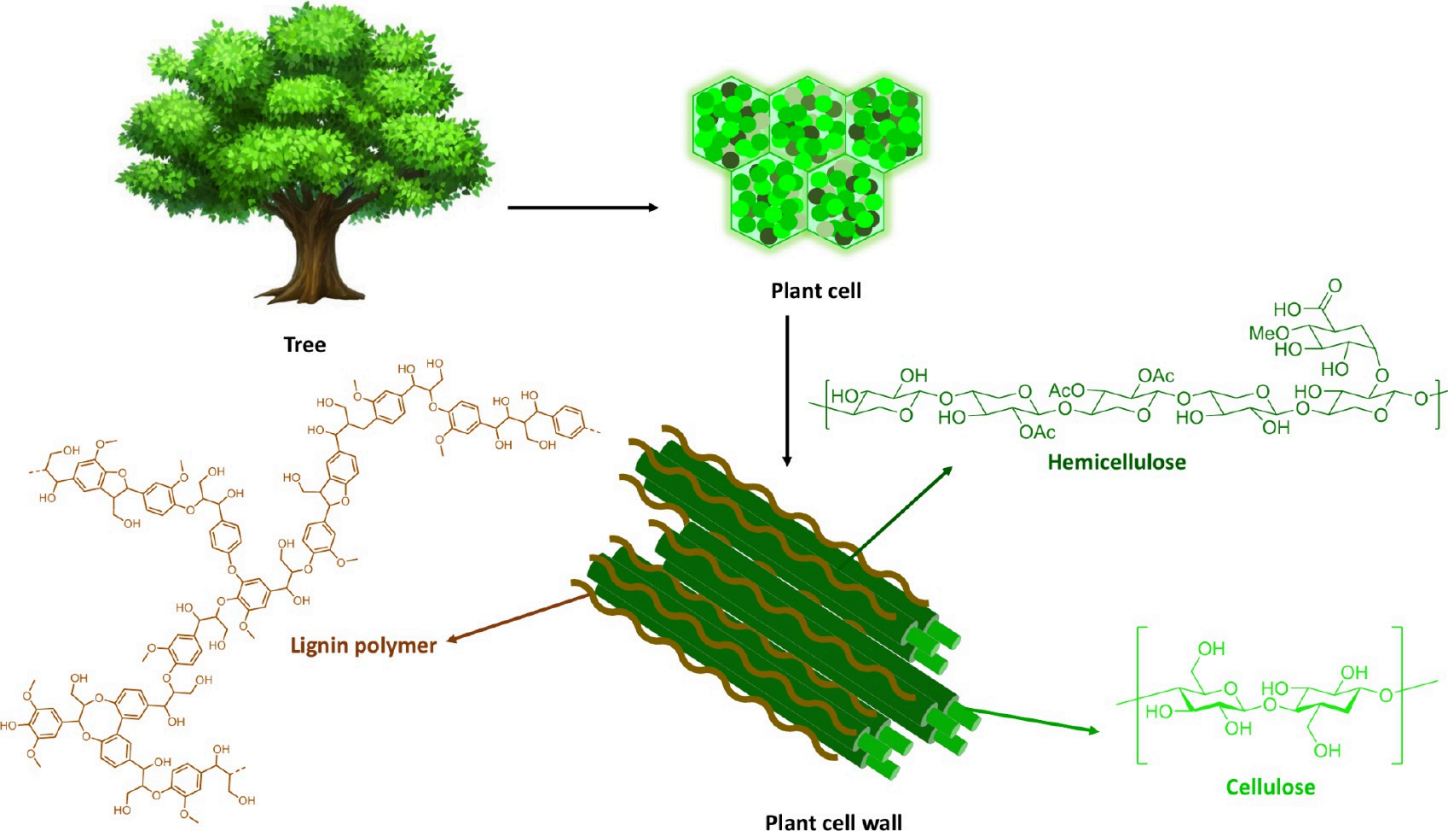
Location of lignin in lignocellulosic biomass.

In the lignocellulosic biomass, the lignin occupies the spaces between the cellulose and the hemicellulose and gives the strength to plant cell wall. The representation of the lignin position is shown in Figure 1.^35^ Lignin is the most abundant, readily available, water-insoluble (pH < 9) and highly functionalized biopolymer enriched in aromatic rings. The complexity and heterogeneity of lignin make it resistant to biological/chemical degradation. Therefore, lignin is considered a low-value byproduct in biomass processing industries such as paper, pulp, bioethanol, etc. For the production of chemicals from lignin, it is important to understand the basic structure of lignin and its properties. The molecular weight and lignin content of lignocellulose depend on the type of wood species and the extraction method. Soft wood contains a relatively higher lignin content (27–33%) than hard wood (18–25%).^36,37^ The lignin content of grasses is in the range of 17–24%. In addition, the lignin structure and the molecular weight of the extracted lignin depend on the isolation method and the process conditions.

**Figure 2:**
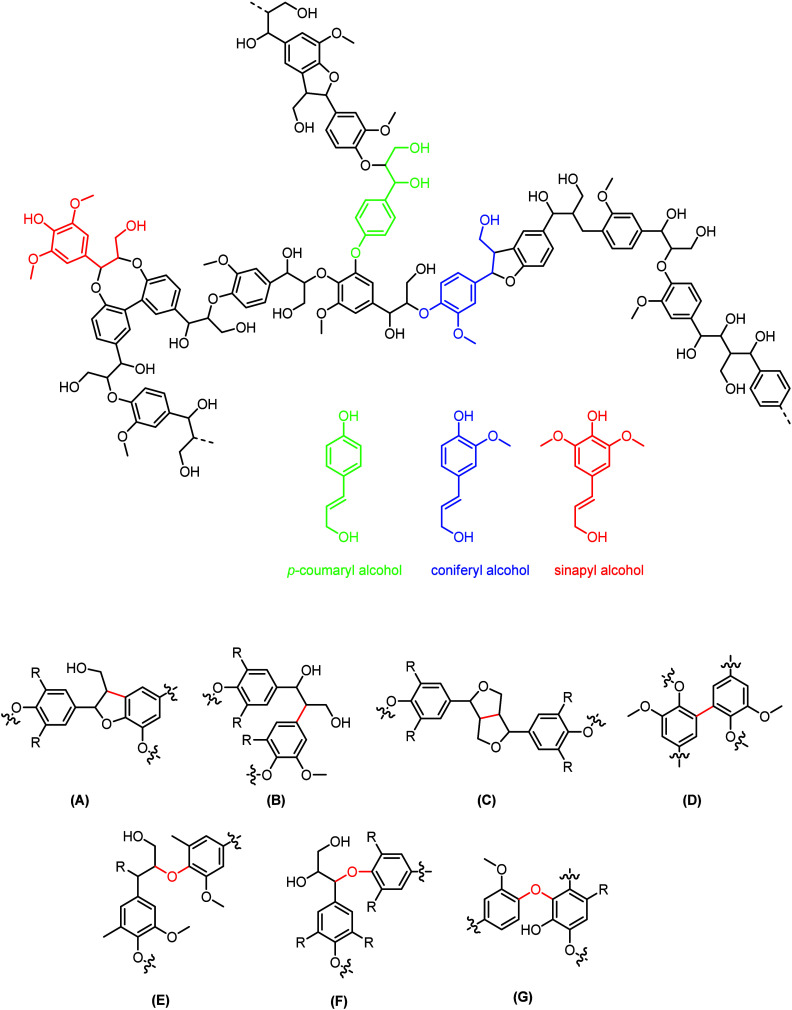
Lignin polymer structure, common phenylpropyl alcohol units, lignin structural motifs, and chemical bonds. Linkages in the lignin polymer: (A) β–5, (B) β–1, (C) β–β, and (D) 5–5 (C–C linkages) and (E) β–O–4, (F) α–O–4, and (G) 4–O–5 (C–O–C linkages).

There is no well-defined structure reported for the native lignin biopolymer. This is due to the structural changes that occur during the extraction of lignin from woody biomass. For example, Li et al. investigated the lignin structure extracted from pine wood using various techniques such as Kraft, organosolv, soda, GVL, IL and formaldehyde pretreatment methods.^38^ The yield of extracted lignin is in the following order Kraft > Organosolv > Soda ≈ formaldehyde-protected > IL-lignin. The results of the HSQC analysis show that the β–O–4 content is relatively low in the case of Kraft (12%) and higher in the case of formaldehyde-protected lignin. For the other lignins, the β–O–4 content is moderate and lies between Kraft and formaldehyde-protected lignin. This indicates that the Kraft process of lignin extraction leads to a greater structural change. It is also reported that the percentage of different functional groups in the extracted lignins is different. The highest content of phenolic hydroxyl, carboxylate and aliphatic hydroxyl was observed in Kraft (1.65 mmol/g), soda (0.7 mmol/g), organosolv (0.4 mmol/g) lignins, respectively. The lowest molecular weight was obtained with IL-lignin and the highest with formaldehyde-protected lignin. The order of molecular weights (*M*_w_) is as follows (low to high): IL-lignin (1280 kDa) < GVL-lignin (1917 kDa) > organosolv-lignin (1988 kDa) < Kraft lignin (2300 kDa) < soda lignin (3650 kDa) < formaldehyde-protected lignin (3844 kDa). In another research article, Xia et al. reported the structural changes of lignins extracted from poplar wood using DES (based on choline chloride and oxalic acid) and a milling process.^39^ Comparison of the ^1^H–^13^C NMR spectra of MWL and DES-lignin showed that the signals originating from β–O–4 bonds disappeared in the *in situ* generated lignin (DES-lignin). These results suggest that the structure of the lignin depends on the method of extraction. A similar type of observation has been made in several research papers.^40−46^ Numerous numbers of research papers have been published to elucidate the structure of lignin. Based on the study of lignin structure using various physical, chemical, and analytical techniques (NMR spectroscopy, GC–MS/FID/TCD, ^31^P NMR FT-IR and GPC etc.), the presence of various chemical bonds and linkages was established and thus the lignin structure was elucidated (see Figure 2).^47^ The structure of lignin was proposed by Erich Adler^48^ in 1977, who identified it as a complex branched polymer consisting of 6-membered aromatics linked by aliphatic hydrocarbons (via C–C and C–O–C bonds), and whose major functional groups are hydroxyl, methoxy, carbonyl, carboxyl groups etc. The recalcitrant units in lignin are phenylpropyl units, namely *p*-coumaryl alcohol, coniferyl alcohol and sinapyl alcohol etc. These building blocks are involved in the polymerization process (via enzymatic dehydrogenation) through nonspecific phenolic radical coupling reactions that form three major units of lignin, namely *p-*hydroxyphenyl (H), guaiacyl (G), and syringyl (S) units.^49^ In addition to the three monolignols, other building blocks such as monolignol conjugates (*p*-hydroxybenzoates, *p*-coumarates), acylated monolignols, monolignol acetates, methylated monolignols, hydroxy cinnamaldehyde, and hydroxycinnamic acid esters, etc. are also found in some wild-type plants.^10,50^ Most of these monolignol moieties are incorporated into the lignin structure in the usual way, with the exception of *p*-hydroxybenzoate and *p-*coumarate, as these molecules do not participate in the radical coupling reaction but are anchored to γ-hydroxyl groups in the lignin. Due to the radical coupling reactions in the formation of lignin polymers, the lignin structure contains a large number and variety of bonds. The most important bonds include β–O–4 (aryl ether), α–O–4 (phenylcoumaran), 5–5 (resinol), β–5 (phenylcoumaran), β–β (resinol), β–1 (1,2-diarylpropane), 4–O–5 (biphenyl ether), etc.^35^ It is also reported that the content of three main building blocks varies in softwood and hardwood. In general, the content of coniferyl and sinapyl alcohols in softwood lignin is between 90–95% and 5–10%, respectively. In hardwood, on the other hand, the composition of coniferyl and sinapyl alcohols is almost the same (50%).^35^ Regardless of the wood species, the proportion of ether bonds predominates among the various chemical bonds in lignin. However, the percentage of β–O–4 bonds is higher in lignin from hardwood (60–65%) than in lignin from softwood (40–45%).^51^ The percentage of different chemical bonds in lignins from softwood and hardwood is shown in Table 1. The cleavage of these chemical bonds in lignin, such as C–C and C–O–C, by chemical, physical, and biological processes can potentially produce valuable chemicals and fuels. However, it is known that the cleavage of the C–O bond is more favorable than that of the C–C bond in the depolymerization of lignin.^9^

**Table 1:** Percentage of the Interunit Linkages in Hardwood and Softwood Lignins (Data Obtained from Ref (37))

bond type		% in soft wood	% in hard wood
C–C	β–5	9–12	6
	β–1	7	7
	β–β	2	3
	5–5	9.5–11	4.5
C–O–C	β–O–4	46	60
	α–O–4	6–8	6–8
	4–O–5	3.5–4	6.5

### 2.2 Classification of Lignin and Its Production

Two main types of lignins are studied in the literature, namely, sulfur-containing (Kraft lignin and lignosulfonate) lignin and sulfur-free lignin (Organosolv and biorefinery stillage).^52,53^ The sulfur-containing lignins originates from the pulp and paper industry, and sulfur-free lignin comes from the solvent extraction process, biorefineries, and soda pulp processes.^54^ Depending on the extraction process, the bonds present in lignin vary and require different temperatures than native lignin for the production of monomers. This is due to the rearrangement of bonds in lignin and the formation of more C–C bonds.^10^ Various extraction methods are described in the literature. Some of the most important and significant isolation processes are discussed here.

#### 2.2.1 Kraft Process

The Kraft process is the most widely used technique in the pulp and paper industry for the production of cellulose. The cellulose is extracted from the lignocellulosic biomass by dissolving the lignin fibers in the black liquor. About 55 million tons of lignin are produced annually using the Kraft process. Four main methods for the isolation of lignin from Kraft pulp are described in the literature, namely, WestVaco (Indulin), LignoForce, LignoBoost, SLRP.^55^ Kraft lignin is produced commercially using this process by companies such as MeadWestvaco and Metso Corporation etc.^22^ In the Kraft process, the woody biomass is subjected to treatment with white liquor (Na_2_S and NaOH, pH > 10, and temperature 165–175 °C) and the cellulose fibers are separated. Thus, the lignin fibers are in the black liquor (which remains after cellulose extraction). The lignin is precipitated from the black liquor by acid treatment. Generally, H_2_SO_4_ or CO_2_ is used to precipitate the colloidal form of the lignin in the black liquor.

#### 2.2.2 Lignosulfonate Process

This is also another common technique used in the pulp and paper industry. Until the 1950s, this was the most popular method for obtaining pulp. The global production of lignosulfonates is about 1.8 million tons. In this process, wood biomass is treated with calcium/magnesium sulfite or bisulfites at a pH of 2–12 and 130–180 °C.^56^ The main reactive species in this method is sulfite or bisulfite, so that the resulting lignin consists of sulfonic acid groups.^36^

#### 2.2.3 Organosolv Process

Another process for the extracting lignin from woody biomass is the organosolv process. In the organosolv process, no sulfur compounds (sulfides or sulfate) are used. Therefore, this process is considered a green one since organic solvents (methanol, ethanol or water/ethanol, THF), organic acids (formic acid, acetic acid), mixtures of organic solvents, and inorganic acids (HCl, H_2_SO_4_) etc. are used for separatation.^57^ The purity of lignin obtained by this process is usually higher than that of lignin obtained by other processes such as Kraft/lignosulfonates. In addition, the organosolv method usually uses mild reaction conditions to maintain the structure of the lignin similar to that of native lignin. Therefore, the complications of producing chemicals by the organosolv method are relatively less than other methods. Among the organosolv processes, Acell is the best known, which was developed by General Electric Corporation in the 1970s. In 1989, Repap Enterprises commissioned a commercial plant to produce Alcell lignin from a hardwood mixture.^58^ In this process, the lignin is extracted from the hardwood mixture in a series of operations at 180–210 °C and 20–35 bar with 40–60% ethanol (50% most common).^37,59^ The efficiency of the organosolv process depends on the type of biomass, e.g., softwood, hardwood or herbaceous plants, etc.^60^ The advantages of this process are its environmentally benign, the efficient recovery of solvents, the high quality of the byproduct (pulp) and the associated additional profits. The main disadvantage of the organosolv process is that it is not economical, as it consumes a lot of solvent and requires expensive solvent recovery.

#### 2.2.4 Biorefinery Stillage

Stillage, also known as distillery effluent, is the residue resulting from the production of ethanol from lignocellulosic biomass.^61^ Stillage is rich in lignin and consists of residual/unused cellulose and hemicellulose fractions. The composition and properties of stillage depend on the unit operations, feedstock, and vary from distillery to distillery. For example, Priharto et al. reported that the lignin content in poplar stillage is 63 wt % of lignin.^62^ For every liter of alcohol produced, up to 20 L of stillage are produced as a byproduct.^63^ Every year, around 113.5 million liters of stillage are produced from biorefineries.^64^ Therefore, stillage from biorefineries could be considered as one of the sulfur-free feedstocks for the production of aromatics.

#### 2.2.5 Pyrolytic Lignin

In pyrolysis, wood is treated for a short time at high temperatures.^65^ The pyrolysis crude oil obtained from biomass consists of sugar and lignin fractions (oligomers) and tends to repolymerize during prolonged storage due to its acidic nature. The crude pyrolysis liquid could be upgraded to valuable chemicals by direct conversion and/or fractionation into its constituents (i.e., sugar and lignin fractions) and subsequent hydrotreatment/hydrodeoxygenation.^66,67^ The crude pyrolysis can be separated into water–soluble and insoluble components. This is the basis and an elementary step for a pyrolytic biorefinery. Therefore, the sugar fraction is separated by water fraction and called as pyrolytic sugar fraction. The insoluble pyrolysis oil, on the other hand, is referred to as pyrolytic lignin. As already mentioned, the chemical structure of pyrolytic lignin differs from that of native lignin or lignin derived from other processes in a similar way to Kraft lignin. This is because bond-breaking and bond-forming reactions take place during the pyrolysis of biomass at high temperatures (>400 °C). Compared to other types of lignin, pyrolytic lignins have a low molecular weight. However, the lignin fragments are very stable and therefore difficult to depolymerize.

However, the lignin structure undergoes several changes during extraction and acid/base treatment.^51^ Compared to native lignin, the extracted lignin is more reactive due to several structural changes that occur during the process, e.g., the formation of several functional groups such as hydroxyl, methoxy, carbonyl, carboxyl, etc. In the case of Kraft and lignosulfonates, about 2–3% sulfur is present. Therefore, lignin extracted from woody biomass consists of various lignin fragments with a wide range of molecular weights and polydispersity. The polydispersity of lignin for the different types of lignin is in the following order: lignosulfonates > Kraft lignin > Soda lignin > Organosolv lignin.^68^

## 3 Lignin Depolymerization Routes

**Figure 3:**
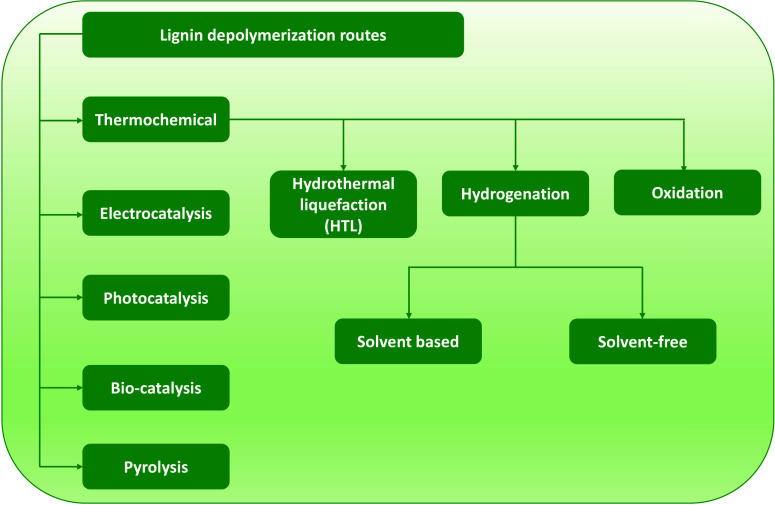
Various lignin depolymerization routes.

In the approaches for depolymerizing lignin, the starting material, lignin, is extracted from the wood biomass. Another approach is the direct depolymerization of woody biomass without extraction of lignin. In the first approach, the percentage of C–C bonds is higher, as the formation of C–C bonds cannot be avoided during the extraction of lignin under harsh conditions. In the second case, on the other hand, the lignin is in its native form, and the percentage of C–C bonds is relatively lower than that of the extracted lignin.^69^ However, the products obtained from wood biomass are complex and require several separation processes, as cellulose and hemicellulose products are also present. The conversion of the original biomass feedstock into the targeted value-added aromatic product requires a detailed understanding of the composition of the biomass, the extraction of the biopolymer (lignin), the main chemical bonds that are more favorable or unfavorable to cleave, the selection of the catalyst, the reaction engineering, the analytical techniques for the analysis of the starting lignin and the downstream products (molecular weight: GPC, CHNS, GC–MS/FID/TCD, NMR) etc. Various approaches for the conversion of lignin into the estimated products have been reported in the literature. These include biocatalytic, electrocatalytic, photocatalytic pyrolysis, hydrolysis, oxidation, and reduction etc. The most studied methods for depolymerization of lignin are shown in Figure 3 and are briefly discussed below:

### 3.1 Biocatalytic Conversion

One of the eco-friendly routes reported in numerous research articles is the biocatalytic conversion of lignin using microorganisms. However, there are some limitations when it comes to industrial applications, as these reactions depend on the temperature, pH and oxygen content in the medium.^70^

### 3.2 Electrocatalysis Route

Numerous approaches have been developed for the electrocatalytic depolymerization of lignin, including hydrodeoxygenation, electrohydrogenation, hydrogenolysis, carbonylation, hydrogenolysis etc. In electrocatalytic lignin depolymerization, the reaction medium/electrolyte is water. Compared to the conventional thermocatalytic route, the electrocatalytic route requires mild reaction conditions. The reaction proceeds via a direct or indirect electron transfer pathway.^71−73^ Electrochemical reduction or oxidation generates free-radicals that accelerate the reaction. However, the electrocatalytic depolymerization of lignin is subject to some limitations as it involves a multielectron process, a high energy barrier for lignin activation (due to the high aromaticity), and competition between water splitting and electrocatalytic reactions.^74^

### 3.3 Photocatalytic Approach

Another alternative green process for the producing chemicals from lignin is the photocatalytic process, which uses mild reaction conditions so that less charring is observed and the catalyst life is longer compared to thermocatalytic processes. For example, the photoelectrocatalytic process avoids the use of hydrogen and high temperatures.^75,76^

### 3.4 Pyrolysis Method

Pyrolysis is one of the most effective technologies for the production of biofuels, chemicals and gases from lignin. Since no oxygen is used in this process, it is considered a simple and economical process. Pyrolysis of lignin requires high temperatures in order to cleave linkages to produce aromatics.^77^ The major drawback of the pyrolysis technique is the formation of a high char.^78,79^ The monomer yield is relatively low in thermal pyrolysis compared to catalytic pyrolysis.^80^

### 3.5 Thermochemical Route

Thermochemical processes can be divided into three categories: hydrothermal liquefaction, oxidation and hydrogenation using homogeneous and heterogeneous catalysts.

#### 3.5.1 Oxidation

The oxidative lignin depolymerization reaction is carried out in the presence of oxygen or hydrogen peroxide, with the main products being vanillin and its derivatives.^22,81^ About 15% of vanillin is produced from lignin by the oxidation process. Copper-based catalysts are best suited for lignin oxidation, as Cu^2+^ ions play an important role in the formation of phenoxy radicals.^82^ Tobias et al. reported 65% bio-oil from Kraft lignin using heteropoly acid, with a 7% of vanillin derivatives.^83^

#### 3.5.2 Hydrogenation

Hydrogenation uses hydrogen or a hydrogen-donating solvent (usually alcohols) or a combination of solvent and hydrogen to depolymerize lignin. The aim of hydrogenation is to remove oxygen from the lignin polymer by hydrodeoxygenation to produce simple aromatics. Factors that affect lignin hydrogenation include the solubility of the lignin in the solvent, the type of lignin, the catalyst, the type of hydrogen donor, high pressures, etc. The most studied nonaqueous hydrogen donor solvents are ethanol and methanol due to the solubility of lignin in these solvents.^84^ The use of inert solvents such as dodecane, hexadecane and tetralin has also been described in the literature for lignin depolymerization.^22^ When choosing the reaction conditions (mild or harsh), it is important to consider the stability of the solvent, as harsh conditions can lead to solvent decomposition.^85^

#### 3.5.3 Solvent-Based Reactions

One of the most studied homogeneous catalysts for the depolymerization of lignin is NaOH/KOH. The use of a base as a catalyst or cocatalyst resulted in improved monomer yield, as the alkaline solution improved the solubility of lignin and thus the degree of depolymerization.^86−88^ However, the disadvantage of homogeneous catalysts is their low thermal stability and the fact that they can be used for optimal conversion of lignin to monomers below 200 °C.^89^ The use of organic solvents and heterogeneous catalysts for lignin depolymerization is discussed in Section 4.

#### 3.5.4 Hydrothermal Liquefaction

HTL is one of the proposed techniques for the depolymerization of lignin.^90^ HTL is a thermochemical route for the conversion of lignin under subcritical or supercritical conditions of water (374.1 °C and 22.1 MPa). The mechanism of the HTL technique involves the depolymerization of macro–biopolymers into microstructural molecules, which in a further step combine to form a new compound (i.e., decomposition of the biopolymer and recombination of the monomers). The main advantages of this technique are 1. Water serves as a solvent. Therefore, materials with high moisture content can be used in this process, which in turn reduces the energy demand as no drying step is required, 2. water under supercritical conditions can be used as a reactant, 3. the HTL process requires mild reaction conditions compared to other processes such as pyrolysis, gasification, 4. the HTL process is versatile and environmentally friendly, can be used in a wide range of applications.^91^ However, the liquefaction of lignin leads to a very low monomer yield of up to 10%.^92^ As mentioned above, several homogeneous and heterogeneous catalytic processes have been developed for the efficient depolymerization of lignin. However, since physical and biological processes are environmentally friendly, the yield of products is low compared to thermochemical routes.^70^

#### 3.5.5 Solvent-Free Reactions

Solvent-free depolymerization of lignins is one of the most promising and green approaches among the other routes described above. In this approach, no external solvent is used and the lignin is processed in solid form together with a heterogeneous catalyst at high temperatures (300–500 °C) and high hydrogen pressures (>100 bar).^26−34^ Therefore, “solvent-free lignin depolymerization” is also generally also called as “solvent-free lignin hydrotreatment”, as this approach is similar to cracking followed by HDS process in petroleum refining. Under the conditions of hydrotreatment, lignin itself serves as a solvent and promotes the depolymerization reactions. One of the main advantages of the solvent-free approach is its potential to reduce production costs when aiming for oxygen-free aromatics. The additional advantages of solvent-free lignin depolymerization over solvent-based techniques are discussed in detail in the Section 4.

## 4 Advantages and Disadvantages of Solvent-Based Depolymerization

Most of the available research and review articles on lignin depolymerization routes are solvent-based. The solvent-based processes have several disadvantages. The production costs increase due to the use and consumption of large amounts of solvents such as methanol, ethanol, acetone etc., as the alcoholic solvents cannot be fully recovered; moreover, they act as one of the reactants that increase the oxygen content in the final product and alter the product selectivity.^93^ Instead of removing oxygen from the lignin monomers, the addition of methoxy or ethoxy groups leads to an increase in the oxygen content in the final products, i.e., alkylphenols, cresols, etc.^94,95^ For example, Ma et al. reported the production of valuable chemicals from Kraft lignin using a nanostructured molybdenum carbide catalyst.^96^ Since the authors used ethanol as a solvent, they achieved a mass balance of more than 100%, i.e., the yield of liquid product is 1.64 g per gram of lignin. This clearly shows that solvents are consumed during hydrodeoxygenation. Moreover, the solvent-based technique is a tedious process as it involves stepwise product processing such as distillation and evaporation. Therefore, solvent-based techniques are questionable in terms of techno-economic viability. In another report, Anand et al. studied the hydrotreatment of Kraft lignin using NiW and NiMo catalysts under supercritical conditions of methanol.^97^ The main products are alkyl phenols/cresols, guaiacolics and catechols, and no significant number of aromatics (without oxygen). The high yield of oxygenated monomeric products is due to the use of methanol as a solvent. This is also evidenced by the influence of reaction time (in the range of 4–24 h) on the product distribution. Initially, guaiacolics are observed as the major product at low reaction times, and high reaction times lead to an increase in alkylphenols yield, indicating an isomerization reaction rather than hydrodeoxygenation. Similar observations were made in the conversion of guaiacols to phenols with a molybdenum carbide catalyst.^98^

To overcome the incorporation of protic solvents into the depolymerized lignin products, another alternative approach is to use oxygen-free solvents (inert or nonprotic) such as dodecane,^99−101^ hexadecane^102−106^ and tetralin^107−109^ etc., which have been reported in the literature. However, in most of the reports, a low yield of oxygen-free aromatics and/or alkylphenolics was observed, although a large amount of solvent was used. Interestingly, Abdus Salam et al.^102^ reported a high yield (28%) of alkylbenzenes with an overall monomer yield of 38.9% in hydrotreatment of Kraft lignin over NiMoS-USY catalyst in hexadecane medium. The high activity of the NiMoS-USY catalyst is due to the close proximity between the acidic and the active site (MoS_2_), which favors the stabilization of the lignin fragments. Based on these results, the authors investigated the unsupported NiMoS catalyst for the hydroconversion of hydrolysis lignin and compared the results with Kraft lignin.^103^ The best results were obtained for the hydrolysis lignin, as it showed a 17.3% higher monomer yield compared to Kraft lignin. The total monomer yield reported for the hydrolysis lignin was 64.3% (including 16% alkylphenolics and 20.1% aromatics) at 400 °C, 80 bar H_2_, 5 h, 10 wt % catalyst. Under optimized reaction conditions (20% catalyst loading), the monomer yield increased further to 76% (including 39% aromatics and 10.1% alkylphenolics). The factors favoring high monomer yield in lignin hydrolysis are (i) low sulfur and ash contents, (ii) structural changes in the lignin, (iii) the presence of small molecules, etc. The main advantage of inert solvents in the depolymerization of lignin is that they promote the reaction by hydroconversion.^22^ Despite the high yield of monomers and aromatics, the main disadvantages of high boiling point solvents and long-chain hydrocarbons in lignin depolymerization are the complex product extraction and the use of large amounts of solvents, which increase production costs, especially on an industrial scale. In addition, cracking of the long-chain hydrocarbons (dodecane, hexadecane etc.) cannot be avoided under hydrotreating conditions and in the presence of acidic supports (e.g., Zeolites). This further complicates the product workup process. Continuous pumping of the lignin together with nonpolar solvents also poses a challenge in fixed-bed reactors.

In contrast to the solvent-based hydrogenation route, the solvent-free conditions are advantageous because, (i) no solvent is used and therefore no change in product selectivity is observed, as the main reactions in this method are hydrodeoxygenation and cracking, (ii) the cost of aromatics production is relatively lower than the solvent-based processes, (iii) product separation by simple decantation/filtration is easy, (iv) the oxygen content in the product is relatively low, (iv) the lignin oil obtained by the solvent-free method can be processed with conventional refinery feedstocks such as VGO.^110^ Despite some advantages, solvent-free depolymerization of lignin also has some disadvantages, (i) no solvent is used in these reactions, and therefore repolymerization of lignin fragments can occur, leading to the formation of char, (ii) hydrogenation of aromatic ring.

The thermochemical hydrogenation reactions are generally carried out under mild or harsh conditions.^111^ Solvent-free lignin depolymerization of lignin requires high pressure and high temperatures. Typically, temperatures of 400–500 °C and up to 100 bar hydrogen are used for the hydrotreatment. The oxygen contained in the lignin is removed in the form of water.^112^ In the following section, the latest developments in the catalytic depolymerization of different lignins under solvent-free conditions are discussed in detail.

## 5 Solvent-Free Lignin Depolymerization

**Figure 4:**
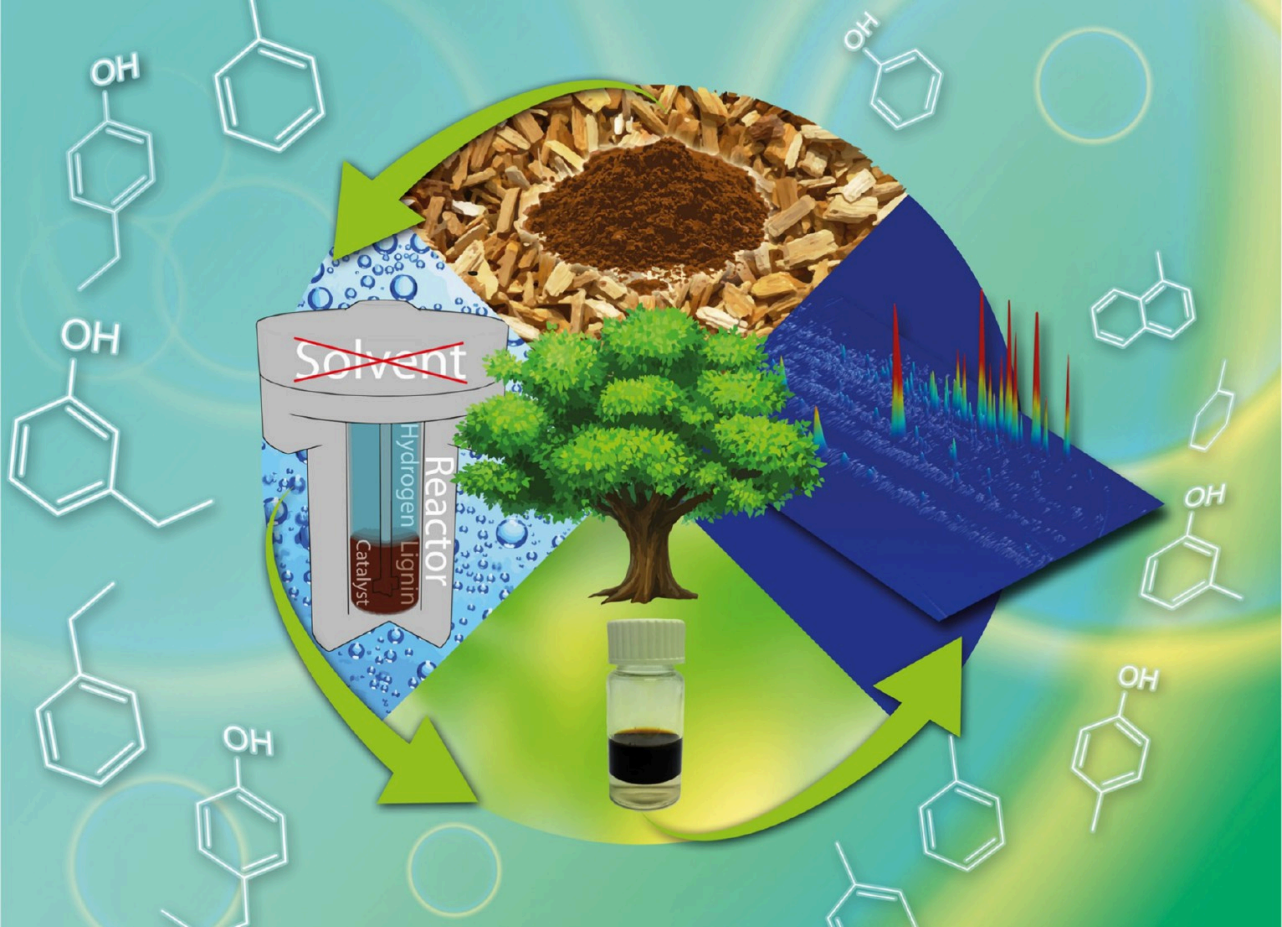
Production of fuels and chemicals from lignin by the solvent-free hydrotreatment process. Reproduced with permission from ref (26). Copyright 2015 RSC Publications.

Solvent-free lignin hydrotreatment is analogous to petroleum refining, where crude oil is the feedstock for the production of chemicals and fuels, whereas in lignin hydrotreatment process, lignin is the source for the production of aromatics and fuels. The main objective of solvent-free lignin hydrotreating is to produce valuable chemicals, such as aromatics with low/no oxygen content. After the solvent-free hydrotreatment of lignin/pyrolysis/bio-oil with heterogeneous catalysts, three phases are usually observed, namely the aqueous phase, the organic phase, and the solid phase. In the aqueous phase, water-soluble products such as acids and some organic compounds were observed. The organic phase, on the other hand, consists of various compounds such as alkylphenolics, aromatics, alkanes (branched/linear/cyclic), ketones, esters, etc. The main objectives of the solvent-free depolymerization of lignin are 1. effective removal of oxygen (hydrodeoxygenation) and low O/C ratio, 2. low yield of gaseous and solid products (char), 3. high yield of monomers, 4. reduction of overall costs. A schematic representation of solvent-free lignin hydrotreatment is shown in Figure 4. There are several approaches for the hydrotreatment of lignin in a single-stage and multistage process, which are described in detail in the following sections.

### 5.1 Hydrotreatment of Lignin in Batch Reactors

#### 5.1.1 Hydrotreatment of Sulfur-Free Lignins

**Figure 5:**
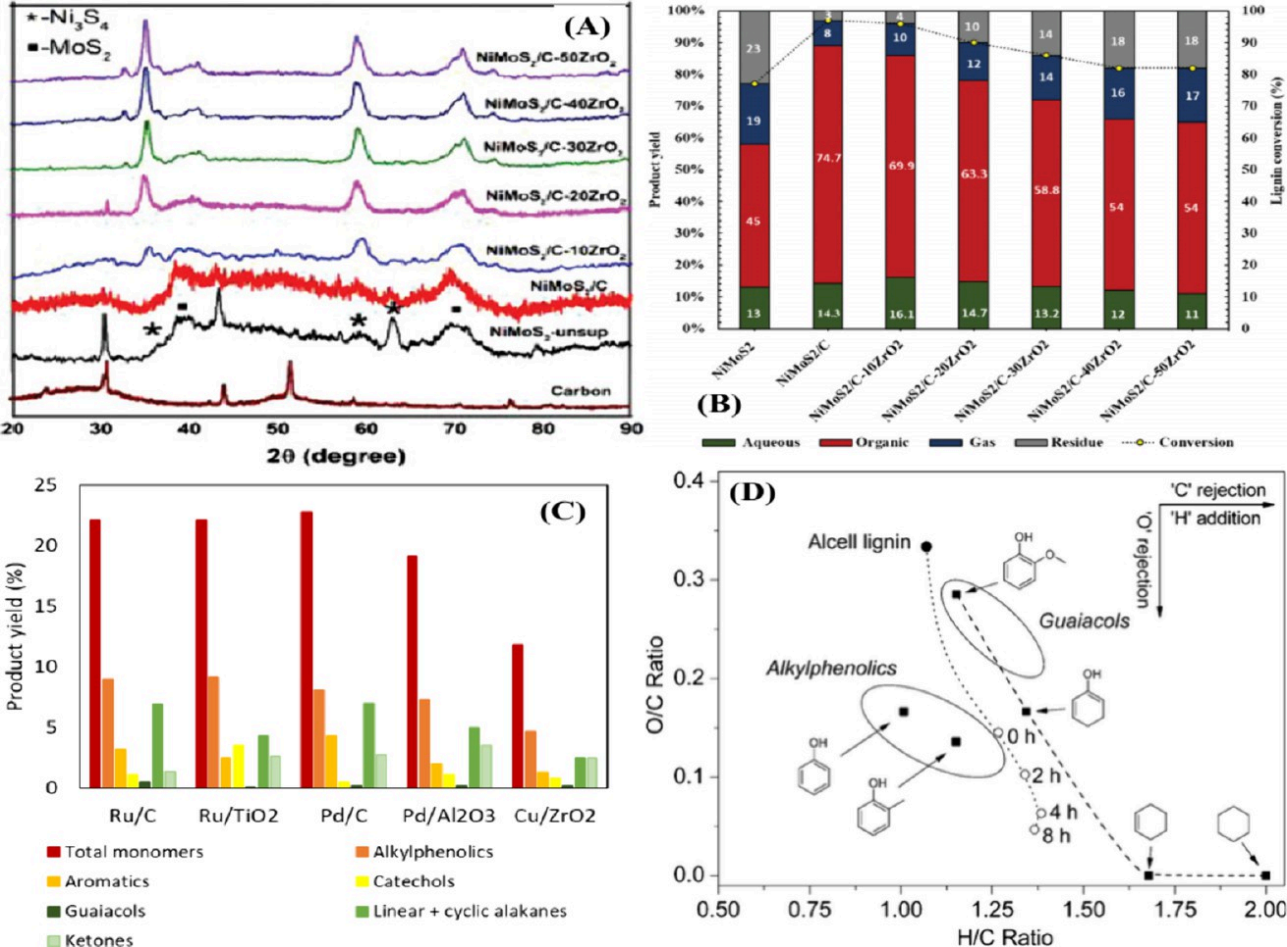
(A) XRD pattern of NiMoS_2_ on C–ZrO_2_ support, (B) conversion of softwood lignin and product yield on the NiMoS_2_/C–*x*ZrO_2_ catalysts. A and B reproduced with permission from ref (113). Copyright 2021 Wiley. (C) monomer yields on the supported noble metal and Cu/ZrO_2_ catalysts^27^ and (D) Van–Krevelen diagram for catalytic hydrotreatment of Alcell lignin on a Ru/C catalyst between 0 and 8 h.^27^

In the catalytic conversion of lignin to value-added chemical reactions, the lignin polymer is cracked and then hydrodeoxygenated. Therefore, the selection of a catalyst for the efficient depolymerization of lignin that has properties such as acidity and the ability to remove oxygen in the form of water is very important. In addition, the solvent-free lignin hydrotreatment reaction requires high temperatures and pressures to obtain organic and aqueous phases through which the products can be easily separated. This section describes the catalytic hydrotreatment of sulfur-free lignins such as Alcell, organosolv, soda etc. From the literature, it is found that the solvent-free catalytic hydrotreatment reaction mainly produces alkylphenolics as the main product and aromatics as the second main product, regardless of the lignin source (sulfur-containing, sulfur-free, pyrolytic lignin, pyrolysis oil, etc.). Mukundan et al. synthesized a series of NiMoS_2_/C–*x*ZrO_2_ catalysts by varying the ZrO_2_ content and tested them for the hydrodeoxygenation of softwood lignin.^113^ XRD analysis showed high dispersion of ZrO_2_ and MoS_2_/Ni in the case of the NiMoS_2_/C–10ZrO_2_ catalyst (Figure 5(A)). The yield of the organic phase depends on the ZrO_2_ content and the acidity of the catalyst. A high ZrO_2_ content led to the formation of char. A high product yield (69.9%) was obtained with the NiMoS_2_/C–10ZrO_2_ catalyst, with 69.1% of the products being detectable by GC due to the presence of tiny MoS_2_ crystals in the catalyst (Figure 5(A,B)). The HDO capability of the NiMoS_2_/C–10ZrO_2_ catalyst is proportional to the reaction time, as the alkylphenolics are highest after 4 h (23.1%) and decrease to 9.4% after 10 h, yielding oxygen-free aromatics. The yield of aromatics increased by 25%, and at the same time a slight increase in the yield of alkanes was also observed (due to ring hydrogenation). In the HDO of alkylphenolics or guaiacols, the cleavage of the aromatic carbon–oxygen (C–O) bond is much more difficult than ring hydrogenation, as reported in the literature.^114−116^ It is known that the HDO reaction of alkylphenolics proceeds through a tautomerization reaction, followed by hydrogenation and dehydrogenation reactions, etc. Therefore, a bifunctional catalyst (acid and metal sites) is required for the HDO of phenolics compounds.^117^ The NiMoS_2_/C–10ZrO_2_ catalyst has NiMoS_2_ and C–ZrO_2_ components, which are responsible for the C–O bond breaking and tautomerization reactions in the formation of aromatics, respectively. These results indicate that the incorporation of the acid in the form of ZrO_2_ and its content in the catalyst has a significant influence on lignin hydrotreatment. The NiMoS_2_/C–10ZrO_2_ catalyst is very stable as it showed consistent activity up to five cycles.

The catalytic activity depends on the active component and the type of support. The activity of the same metal on different supports shows large differences in lignin hydrotreatment. For example, Arjan et al. reported the catalytic hydrotreatment of Alcell lignin using noble metal (Ru, Pd, and Cu) on carbon, alumina, and titania supports.^27^ Since noble metal catalysts were used for the study, the formation of char was negligible in all the cases, and the yield of oil produced was up to 78%. Compared to the noble metal catalysts, the Cu/ZrO_2_ catalyst exhibited low activity, as the total yield of monomers in this case was only 11.8% (Figure 5(C)). The major differences in activity between noble metals and copper are due to the use of the reduced (Pd and Ru) and oxide (CuO) forms of the catalysts. When comparing Pd/C and Pd/Al_2_O_3_ catalysts, the best result was obtained in the case of carbon supports (22.8%). In contrast, Ru on carbon and TiO_2_ supports showed similar activity with an overall monomer yield of 22.1%. However, in the case of the Ru/TiO_2_ catalyst, the authors considered valuable products such as alkylphenolics (9.1%), catechols (3.5%) and aromatics (2.5%). However, these values are close to the products obtained in the case of Ru/C. Compared to the Ru/TiO_2_ catalyst, the Ru/C is less expensive. For this reason, the authors could consider the Ru/C catalyst for the optimization study. It was found that the total monomer yield increased from 12.9 to 29.7% at 0–8 h and the oxygen content also decreased (Figure 5(D)). This indicates that the reaction time has a major influence on lignin depolymerization and oxygen content. It is also clear that the HDO reaction is more pronounced at high reaction times, as evidenced by an increase in aromatics of 1.3–5.2%. Based on the activity results, it is clear that mild acidic or neutral supports are efficient in the hydrotreatment of lignin for aromatics production. The authors proposed a reaction network for the various gas and liquid phase reactions during Alcell lignin hydrotreatment.

**Figure 6:**
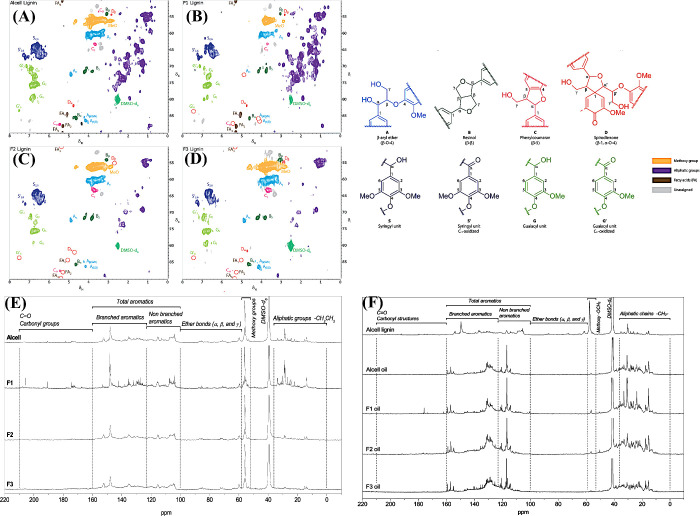
HSQC spectra of (A) Alcell lignin, (B) fraction F1, (C) fraction F2, (D) fraction F3, (E) ^13^C NMR spectra, and (F) hydrotreated Alcell lignin and its fractions (F1, extracted using diethyl ether; F2, methanol; F3, residue). Reproduced with permission from ref (118). Copyright 2016 RSC Publications.

Besides the catalytic aspects, fractionation and valorization of the lignins is one of the new strategies. Since lignin contains a range of polymer sizes, different lignin fractions can be separated for efficient hydrotreatment of lignin under solvent-free conditions. Fractionated Alcell lignin was used for hydrotreatment with Ru/C catalyst.^118^ The molecular weight of the original Alcell lignin, lignin fractions from diethyl ether (F1), methanol (F2) and residues (F3) were 1720, 660, 1640, and 3680 Da, respectively. The total yield of GC-detectable monomers from the different fractions is in the following order: F1 (27.6%) > Alcell lignin (21.4%) > F2 (17.2%) > F3 (13.4%), in reverse order to the molecular weight of the respective lignin fractions. Other important factors influencing the monomer yield are the molecular structure of the lignin fraction and the content of aliphatic/aromatic groups in the lignin fraction. The HSQC spectra of Alcell lignin and its fractions showed a great difference in terms of various bonds such as β–O–4, β–β, and β–5 etc. (Figure 6(A–D)). Since β–O–4 bonds are easy to cleave to depolymerize lignin, the content of this bond increases from F1–F3. Therefore, fractionation and detailed analysis of lignin contribute to efficient depolymerization. The ^13^C NMR spectra (Figure 6(E)) clearly show that the Alcell lignin has very low intensities for all types of functional groups. On the other hand, peaks with high intensities for different functional groups were observed in the lignin fractions, indicating that a range of lignin polymers (fractions) are present in Alcell lignin. In the case of Alcell lignin, moderate peak intensity was observed for aliphatic groups. For the lignin fractions, the peak intensity decreased in association with the aliphatic region from F1 to F3 (Figure 6(E)). Therefore, lignin fraction F1 produced a larger number of monomers than the other fractions. This was also confirmed by ^13^C NMR analysis (Figure 6(F)), which showed that after hydrotreating lignin and its fractions, the intensity of peaks in the aliphatic and aromatic regions increased and no methoxy groups were found in all lignin oils. Extraction of different fractions from lignin and subsequent hydrotreatment of lignin is one of the innovative strategies that helps in selecting a suitable catalyst based on the bonds present in the extracted fraction for efficient depolymerization.

**Figure 7:**
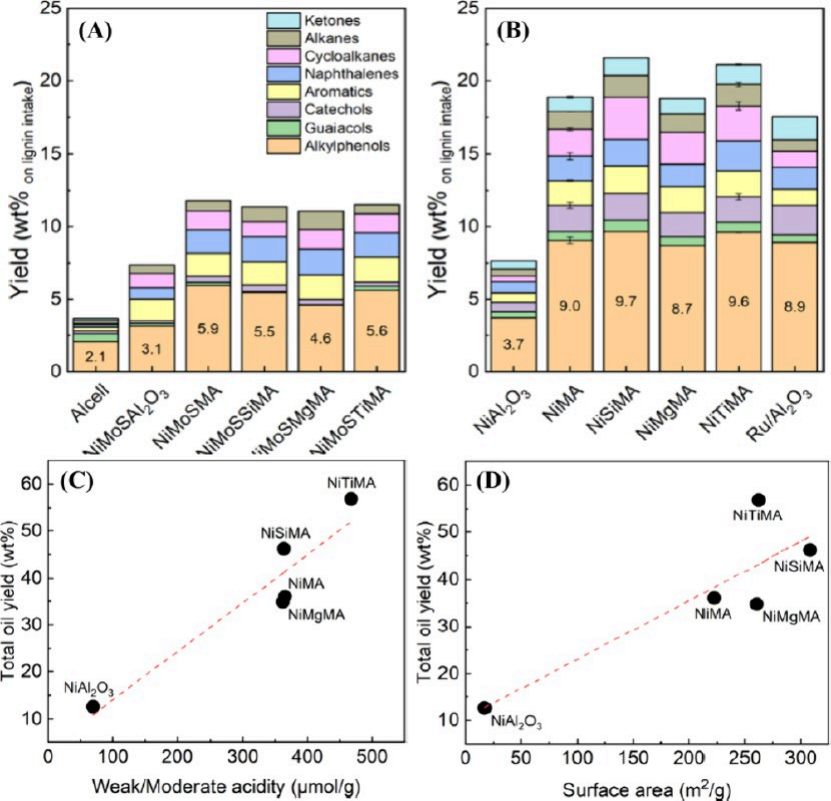
(A,B) Product yield from hydrotreated Alcell lignin over mono- and bimetallic Ni/Mo on ordered mesoporous alumina supports, (C) correlation between oil yield and catalyst acidity, and (D) dependence of oil yield on catalyst surface area.^28^

As already mentioned, weakly acidic or neutral supports are best suited for solvent-free lignin hydrotreatment. This is because the high acidity of the catalyst leads to the formation of char. In contrast, even high acidity of the catalyst promotes the hydrotreatment reaction when the supports are doped with suitable metals. Recently, Yang and co-workers reported mono- and bimetallic Ni/Mo on ordered mesoporous alumina for the hydrotreatment of Alcell lignin and also investigated the effects of doping with elements such as Si, Mg, Ti, etc.^28^ Two different strategies were used for the activity measurements, i.e., mono- and bimetallic catalysts in the form of reduced and sulfided phases, respectively. Among the catalysts investigated, the monometallic Ni catalysts clearly showed better performance (monomer yield: 7.5–22%) than the sulfided bimetallic catalysts (monomer yield: 7–11.5%), owing to the surface area and the total acidity (Figure 7(A,B)). The correlation of the activity of the monometallic catalysts with surface area and total acidity is shown in Figure 7(C,D). The high activity of Si- and Ti-doped catalysts (NiSiMA and NiTiMA) is due to the synergistic metal–support interactions, acidity, and oxygen vacancies. Since titania is known for the presence of oxygen vacancies, which acts as active sites, they showed higher activity (NiTiMA, 57 wt % oil yield, and 10 wt % alkylphenolics) than Si-doped and Ru/Al_2_O_3_ catalysts. In addition, the author reports that the ordered mesoporous alumina support is best suited to achieve high yields of monomers compared to conventional alumina supports. This is due to the presence of mesopores through which the lignin fragments can diffuse, a strong interaction of the metal with support, a large surface area (up to 308 m^2^/g) leading to high dispersion, and tiny Ni particles. This work shows that the high acidity of the catalyst with suitable dopants such as Ti can increase the monomer yield.

**Figure 8:**
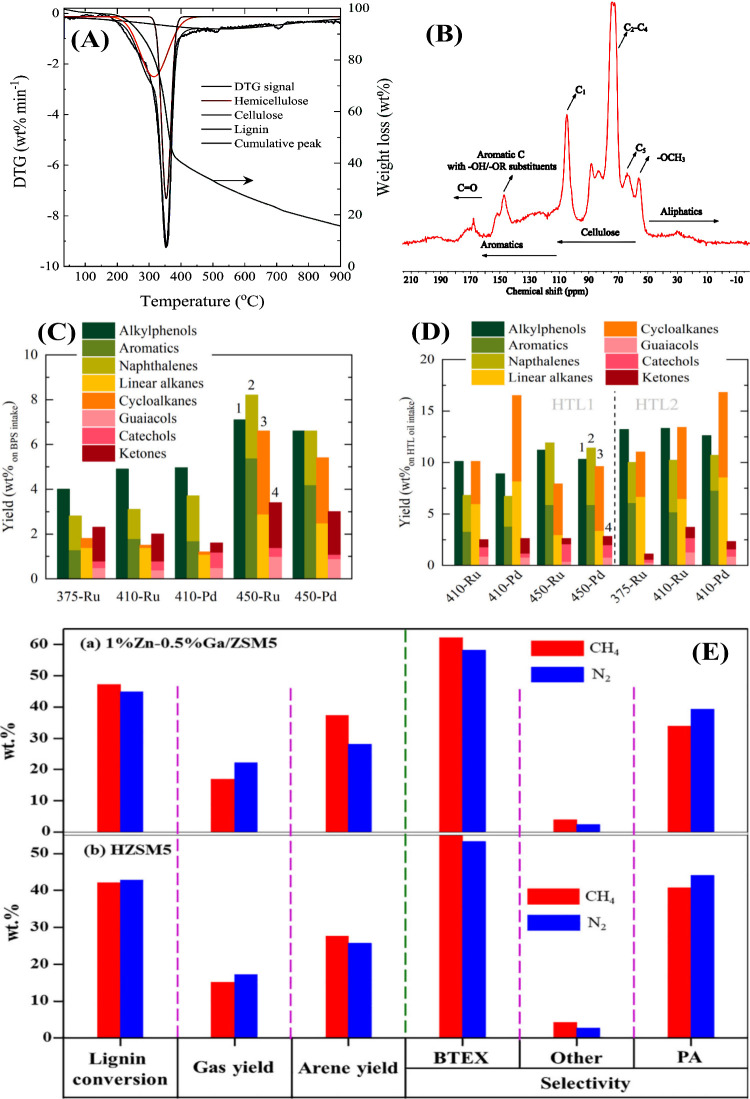
(A) TGA/DTA curves of the decomposition of BPS, (B) solid state CP/MAS ^13^C NMR spectra of BPS, (C) distribution of monomer yield in hydrotreated BPS, (D) distribution of monomer yield in HTL oil over Ru and Pd catalysts. (A–D) reproduced with permission from ref (119). Copyright 2021 Elsevier. (E) Comparison of the performance of HZSM–5 and 1% Zn–0.5% Ga/ZSM-5 catalysts for lignin depolymerization under N_2_ and CH_4_ environments. Reproduced with permission from ref (29). Copyright 2018 Elsevier.

Hita et al. reported a comparative study of a one- and two-step approach for the depolymerization of stillage, i.e., hydrothermal liquefaction and hydrodeoxygenation using Pd and Ru on carbon catalysts.^119^ The stillage used in this study consists of 36% cellulose, 31% hemicellulose, and 33% lignin fractions (Figure 8(A), TGA/DTA analysis). This was also confirmed by solid-state CP/MAS ^13^C NMR (Figure 8(B)), as evidenced by the presence of intense bands associated with the sugar fractions (δ 102 ppm: C1, δ 75 ppm: C2–C4, δ 64 ppm of C5) together with lignin (δ 50–59 ppm: methoxy, δ 110–160 ppm: aromatic C=C bond, δ 165–180 ppm: C=O bond). Up to 410 °C, alkylphenolics were the major product in the direct HDO studies, and at 450 °C aromatics predominated. The Ru/C catalyst gave the highest monomer yield of 25.2% (Figure 8(C)). In contrast, the HTL of stillage (305 °C) yielded only 10% monomers. In the HTL process with subsequent HDO process, the yield of valuable products (alkylphenolics + aromatics) was almost as high as in the one-step HDO approach (based on the lignin uptake, the monomer yields are 13.2 and 12.3% for HDO and HTL–HDO, respectively; Note: Figure 8(D) shows the monomer yields for HTL oil). The advantages of the HTL process over direct HDO are, i. easy pumping of the feedstock in continuous reactors, ii. prevention of coke formation, which increases the stability of the catalysts and their long-term activity. The energy efficiency and monomer yields in single-stage HTL/HDO and two-stage HTL–HDO processing of stillage are in the following order: HTL1 (305 °C) of stillage 98.4% > HTL2 (350 °C) 92.7% > HDO (83.5%, Ru/C at 375 °C) > HTL2–HDO (81.9%, Ru/C at 375 °C) > HTL1–HDO (74.8%, Pd/C). Finally, this study shows that HDO is a more appropriate approach than HTL or two-stage HDO–HTL strategies.

So far, hydrogen has been used for the hydrotreatment of lignin under solvent-free conditions. It is known that in petroleum refineries, methane is used for hydrogen production (methane reforming), and the excess methane is generally burned, which is called flare gas. It is very interesting to use two low-value reactants (lignin and natural gas) to produce valuable chemicals such as oxygen-free aromatics (BTX) and others. Interestingly, Wang et al. used methane as a hydrogen source for the depolymerization of alkali lignin^29^ using zeolites doped with Zn, Ga and Ru. The best results were obtained with H–ZSM5 compared to H−β, and H–Y zeolites due to the moderate pore size (HZSM5 (5.5 Å) > H−β (6.6 Å) > H–Y (7.4 Å)), which can prevent the formation of larger molecules during lignin depolymerization. The acidity of the zeolite plays a key role in the conversion of lignin and the formation of char; the best results were obtained at a Si/Al ratio of 30. The Zn–containing H–ZSM5 catalyst has no significant effect on lignin conversion, but improves the product selectivity of arene and BTEX. On the other hand, the addition of Ga to the zeolite increases the lignin conversion, but the selectivity of BTEX is limited. The combination of the properties of Ga and Zn leads to a significant improvement in arene yield (37.4%) and lignin conversion (48.2%). The noble metal catalyst (Ru–ZSM5) exhibited moderate activity, which could be due to the methane environment. Comparison of the experiments conducted in methane and nitrogen environments showed that methane has no significant effect on lignin conversion (Figure 8(E)). However, methane is incorporated into the final products in the form of methyl groups and is involved in the coaromatization of alkyl moieties, as shown by isotope labeling experiments with ^13^CH_4_. The role of metal ions (Ga, Zn) in zeolites leads to 1. redistribution of Bronsted and Lewis acid sites, 2. enhanced methane activation, 3. improved catalytic performance for the production of arenes with high BTEX selectivity.

The above-discussed different catalytic systems reported for sulfur lignins hydrotreatment are summarized in Table 2. Table 2 shows that mono- and bimetallic catalysts are used for the hydrotreatment of sulfur-free lignin under solvent-free conditions. The noble metals are the most studied catalytic systems for sulfur-free lignin. The Ru/C catalyst exhibited the best performance in the production of aromatics, independent of the lignin source. Moderate yields were observed for other transition metal supported catalysts. The highest yield of aromatic was observed with the metal sulfides, NiMoS_2_/C–10ZrO_2_. Interestingly, methane was used instead of hydrogen for the depolymerization of lignin and showed promising monomer yields.

**Table 2:** Solvent-Free Hydrotreatment of Sulfur-Free Lignins over Various Heterogeneous Catalysts

S. no.	lignin	catalyst	reaction conditions	total monomers yield (%)^a^	major product-alkylphenolics (%)	ref
1.	softwood lignin	NiMoS_2_/C–10ZrO_2_	300 °C, 80 bar H_2_ at RT, 3 h	69.1	23.1	(113)
2.	softwood lignin	NiMoS_2_/C–10ZrO_2_	300 °C, 80 bar H_2_ at RT, 10 h	58.3	25.2 (aromatics)	(113)
3.	Alcell	Ru/C	400 °C, 100 bar H_2_ at RT, 4 h	22.1	9.0	(27)
4.	Alcell	Ru/TiO_2_	400 °C, 100 bar H_2_ at RT, 4 h	22.1	9.1	(27)
5.	Alcell	Pd/C	400 °C, 100 bar H_2_ at RT, 4 h	22.8	8.1	(27)
6.	Alcell	Pd/Al_2_O_3_	400 °C, 100 bar H_2_ at RT, 4 h	19.1	7.3	(27)
7.	Alcell	Cu/ZrO_2_	400 °C, 125 bar H_2_ at RT, 4 h	11.8	4.7	(27)
8.	Alcell	Ru/C	400 °C, 100 bar H_2_ at RT, 0 h	12.9	5.6	(27)
9.	Alcell	Ru/C	400 °C, 100 bar H_2_ at RT, 2 h	20.5	10.3	(27)
10.	Alcell	Ru/C	400 °C, 100 bar H_2_ at RT, 8 h	29.7	12.2	(27)
11.	Alcell	Ru/C	400 °C, 100 bar H_2_ at RT, 4 h	21.4	9.0	(118)
12.	Alcell lignin fraction from ether (F1)	Ru/C	400 °C, 100 bar H_2_ at RT, 4 h	27.6	8.4	(118)
13.	Alcell lignin fraction from alcohol (F2)	Ru/C	400 °C, 100 bar H_2_ at RT, 4 h	17.2	5.3	(118)
14.	Alcell lignin fraction residue (F3)	Ru/C	400 °C, 100 bar H_2_ at RT, 4 h	13.4	6.8	(118)
15.	Alcell	NiMoSAl_2_O_3_	400 °C, 100 bar H_2_ at RT, 4 h	∼7.0	3.1	(28)
16.	Alcell	NiMoSMA	400 °C, 100 bar H_2_ at RT, 4 h	∼11.5	5.9	(28)
17.	Alcell	NiMoSSiMA	400 °C, 100 bar H_2_ at RT, 4 h	∼11.0	5.5	(28)
18.	Alcell	NiMoSMgMA	400 °C, 100 bar H_2_ at RT, 4 h	∼10.8	4.6	(28)
19.	Alcell	NiMoSTiMA	400 °C, 100 bar H_2_ at RT, 4 h	∼11.2	5.6	(28)
20.	Alcell	NiAl_2_O_3_	400 °C, 100 bar H_2_ at RT, 4 h	∼7.5	3.7	(28)
21.	Alcell	NiMA	400 °C, 100 bar H_2_ at RT, 4 h	∼18.5	9.0	(28)
22.	Alcell	NiSiMA	400 °C, 100 bar H_2_ at RT, 4 h	∼22.0	9.7	(28)
23.	Alcell	NiMgMA	400 °C, 100 bar H_2_ at RT, 4 h	∼18.5	8.7	(28)
24.	Alcell	NiTiMA	400 °C, 100 bar H_2_ at RT, 4 h	∼20.6	9.6	(28)
25.	Alcell	Ru/Al_2_O_3_	400 °C, 100 bar H_2_ at RT, 4 h	∼17.0	8.9	(28)
26.	stillage	Ru/C	375 °C, 100 bar H_2_ at RT, 4 h	∼10.8	4.0	(119)
27.	stillage	Ru/C	410 °C, 100 bar H_2_ at RT, 4 h	11.5	4.9	(119)
28.	stillage	Ru/C	450 °C, 100 bar H_2_ at RT, 4 h	25.2	7.5	(119)
29.	stillage	Pd/C	410 °C, 100 bar H_2_ at RT, 4 h	11.4	5.0	(119)
30.	stillage	Pd/C	450 °C, 100 bar H_2_ at RT, 4 h	22.9	6.6	(119)
31.	HTL oil–1^b^	Ru/C	410 °C, 100 bar H_2_ at RT, 4 h	15.6	5.4	(119)
32.	HTL oil–2^c^	Ru/C	410 °C, 100 bar H_2_ at RT, 4 h	17.6	5.8	(119)
33.	HTL oil–1	Ru/C	450 °C, 100 bar H_2_ at RT, 4 h	17.9	6.0	(119)
34.	HTL oil–1	Pd/C	410 °C, 100 bar H_2_ at RT, 4 h	18.5	4.7	(119)
35.	HTL oil–2	Pd/C	410 °C, 100 bar H_2_ at RT, 4 h	18.4	5.5	(119)
36.	HTL oil–1	Pd/C	450 °C, 100 bar H_2_ at RT, 4 h	18.1	5.5	(119)
37.	alkali lignin	H–ZSM5 (Si/Al = 30)	400 °C, 15 bar CH_4_ at RT, 1 h	27.6 ± 0.9	-	(29)
38.	alkali lignin	1%Zn/ZSM5	400 °C, 15 bar CH_4_ at RT, 1 h	31.0 ± 1.4	-	(29)
39.	alkali lignin	5%Zn/ZSM5	400 °C, 15 bar CH_4_ at RT, 1 h	24.2 ± 1.2	-	(29)
40.	alkali lignin	0.5%Ga/ZSM5	400 °C, 15 bar CH_4_ at RT, 1 h	33.5 ± 2.0	-	(29)
41.	alkali lignin	1%Ga/ZSM5	400 °C, 15 bar CH_4_ at RT, 1 h	34.1 ± 2.3	-	(29)
42.	alkali lignin	1%Ru/ZSM5	400 °C, 15 bar CH_4_ at RT, 1 h	28.8 ± 1.9	-	(29)
43.	alkali lignin	1%Zn–0.5%Ga/ZSM5	400 °C, 15 bar CH_4_ at RT, 1 h	37.4 ± 2.5	-	(29)

a Total monomers: alkylphenolics + oxygen-free aromatics + guaiacols + catechols + liner/branched alkanes + cyclic alkanes + ketones + alcohols.

b HTL oil-1: Derived from stillage at 305 °C,

c HTL oil-2: Derived from stillage at 350 °C.

#### 5.1.2 Hydrotreatment of Sulfur-Containing Lignins

**Figure 9:**
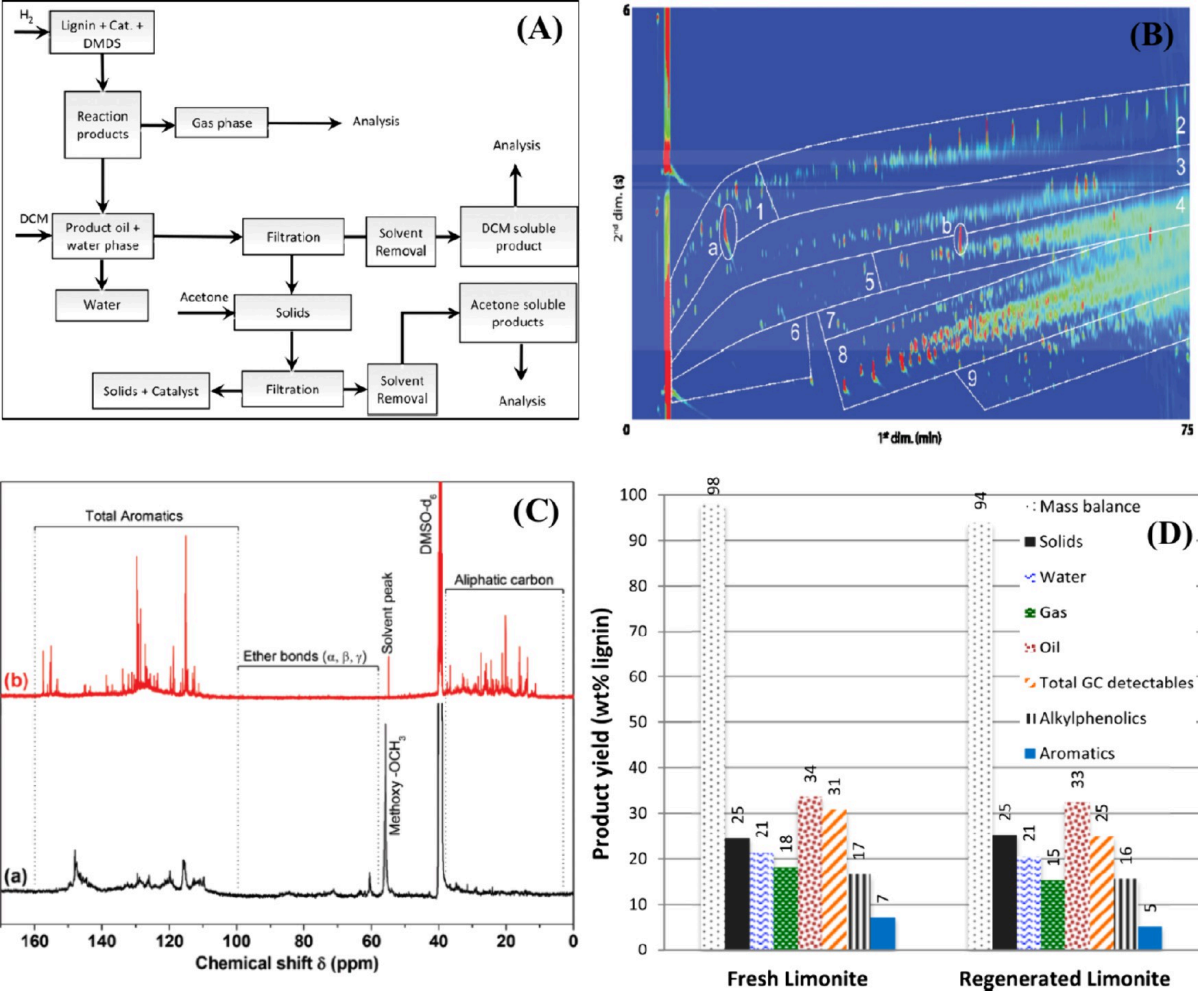
(A) Solvent-based product separation method for hydrotreated Kraft lignin, (B) GC×GC-FID chromatogram of the hydrotreated lignin oil (DSPs) obtained in the case of S-NiMo/MgO–La_2_O_3_ ((1) cyclic alkanes, (2) linear/branched alkanes, (3) aromatics, (4) polycyclic aromatics, (5) ketones/alcohols, (6) acids, (7) guaiacols, (8) alkyl phenolics, (9) catechols. “a” refers to internal standard (di-n-butylether), and “b” refers to 2,5-di-t-butylhydroxytoluene (stabilizer in THF)). (C) ^13^C NMR spectra of Kraft lignin (a), DSPs (b) obtained over the S-NiMo/MgO–La_2_O_3_ catalyst. (A–C) reproduced with permission from ref (26). Copyright RSC Publications. (D) Comparison of product yields obtained for the catalytic hydrotreatment of Kraft lignin using limonite and regenerated limonite catalyst (450 °C, 4 h, 100 bar H_2_ initial pressure, 5 wt % catalyst).^125^

As mentioned in the introduction, Kraft lignin is produced in large quantities as a byproduct of the pulp and paper industry. Lignin from the Kraft process is only soluble in alkaline solutions with a pH value >10. During the Kraft process, sulfur is incorporated into the lignin. Depending on the type of extraction, the sulfur content in Kraft lignin is generally between 2–3%. Catalytic hydrotreatment of Kraft lignin includes hydrodeoxygenation, hydrodesulfurization, hydrogenolysis (C–C and C–O bond breaking). The presence of sulfur in Kraft lignin has some advantages and disadvantages. The disadvantage is the deactivation of the catalyst during hydrotreatment. For example, if we use catalysts such as noble metals (Pd, Ru, Pt), the activity decreases rapidly due to the poisoning of the catalyst with sulfur.^120,121^ The presence of sulfur in Kraft lignin is advantageous for conventional hydrotreatment catalysts, such as Ni/Co promoted Mo/W based catalysts, which are known for the hydrodesulfurization process in petroleum refineries.^122−124^ Since these catalysts are active in the sulfide phase, the sulfur present in the Kraft lignin keeps the catalyst active to a certain extent during the hydrotreatment reaction. In our previous study, NiMo and CoMo on acidic (Al_2_O_3_, ZSM–5), basic (MgO–La_2_O_3_), and neutral supports (activated carbon) were used for the depolymerization of Kraft lignin.^26^ A solvent-based technique was developed to extract the product (Figure 9(A)). The oxygen content in the hydrotreated Kraft lignin was significantly reduced, i.e., the O/C ratio decreased from 0.37 to 0.08–0.13. The ^13^C–NMR study showed that the ether bonds disappeared, indicating that most of the C–O–C bonds were broken under the hydrotreatment conditions (Figure 9(C)). The products were quantified by GC×GC (Figure 9(B)).^26^ In all cases, the main product was alkylphenolics, followed by oxygen-free aromatics. It should be noted that the yield of monomers in the case of NiMo on acidic supports (Al_2_O_3_, ZSM–5) was relatively low due to repolymerization and the formation of char. In contrast, a high monomer yield was achieved on the basic support (NiMo/MgO–La_2_O_3_, monomer yield 26.4% with the alkylphenolics accounting for 15.7%). In the case of sulfur containing lignin, Ni/Co-promoted Mo catalysts were also relatively less active on acidic supports than on neutral/basic supports.

In another report, inexpensive iron ores (limonite, goethite, iron disulfide) are used as a catalyst for the hydrotreatment of Kraft lignin in a high-pressure batch reactor.^125^ In the reactions carried out at 350–400 °C, the yield of monomers was low (max. 17.5%). However, a drastic increase in the yield of monomers (30.9%) was observed when the reaction was carried out at 450 °C (Figure 9(D)), and at the same time, the yield of char also increased with an increase in temperature from 350–450 °C. Since minor amount of char was formed at 350 °C, a high yield of monomers is generally expected. However, the monomer yield was low due to the presence of a large number of oligomers that were not detectable by GC, which was confirmed by GPC analysis (higher molecular weight of the corresponding lignin oil). The monomer yield obtained for the limonite catalysts is comparable to that of the commercial CoMo catalyst and exhibited higher activity than other iron ores (limonite (30.9%) ≥ CoMo (29.4%) > iron disulfide (23.6%) ≥ goethite (23.5%)). However, the CoMo catalyst was best suited for the production of aromatics rather than alkylphenolics, as the yield of aromatics is twice as high (8%) with the CoMo catalyst. This is because the Co/Ni promoted MoS_2_ catalysts are known to effectively remove heteroatoms such as O, N, and S etc. Therefore, by using natural iron ores, we can replace the conventional hydrotreating catalysts for the hydrotreatment of sulfur containing lignins. This could greatly reduce production cost. However, the reusability and stability of the catalyst must be verified.

**Figure 10:**
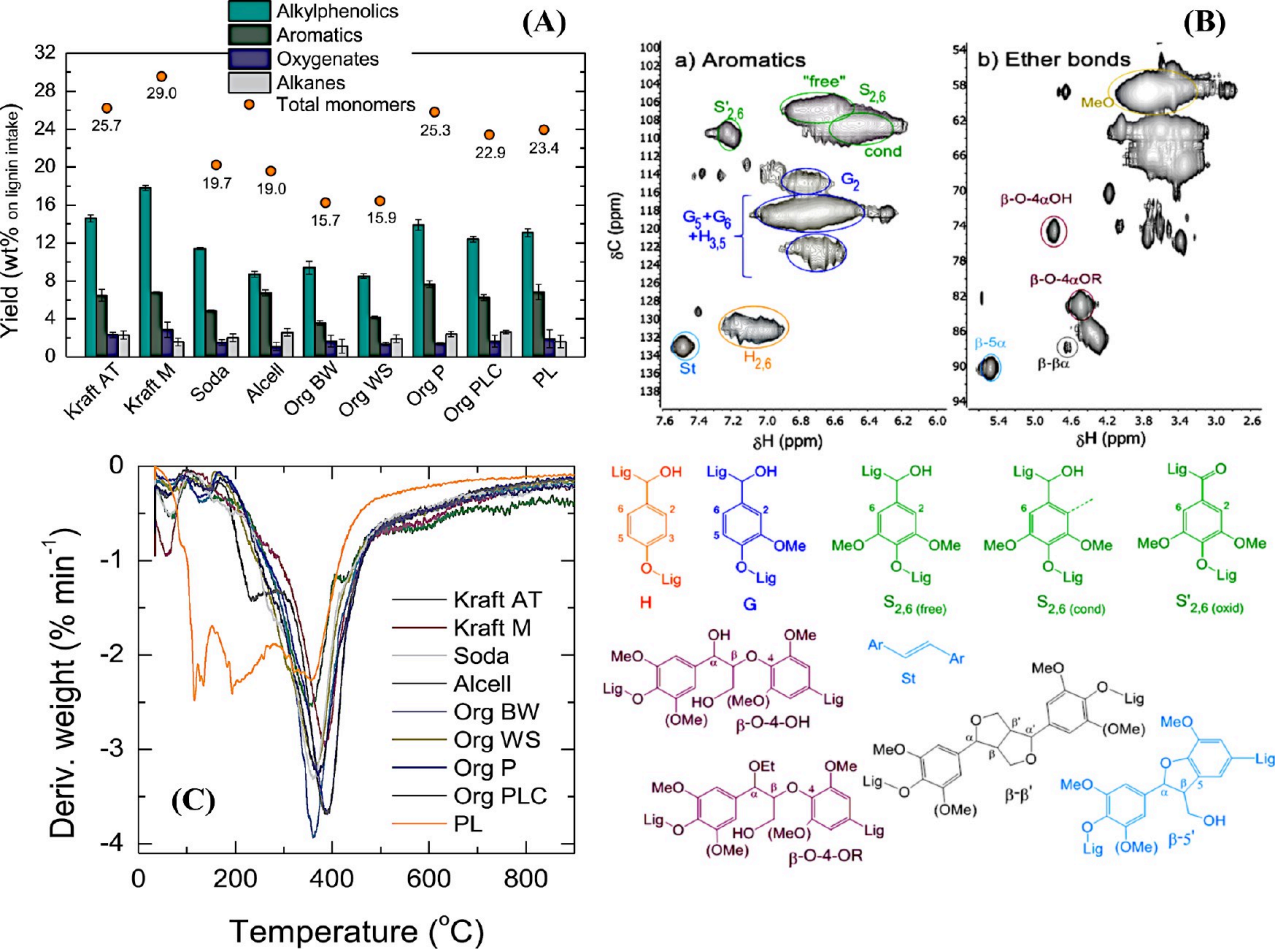
(A) Monomer yields obtained for various lignins using limonite catalyst (450 °C, 100 bar H_2_, 4 h), (B) 2D ^1^H–^31^C–HSQC NMR spectrum examples (d_6_-dimethylsulfoxide) showing (a) an aromatic spectra region for soda lignin and (b) ether linkage spectra region for the Org PLC lignin, which are representative examples of each type of structure, and (C) DTG curves of different lignins. Reproduced with permission from ref (126). Copyright 2018 Elsevier.

Apart from the reaction conditions for lignin depolymerization, other factors affect lignin depolymerization and total oil yield are complexity, source and extraction methods. This is due to the structural changes in the lignin during extraction conditions, such as high temperatures, type of solvents and chemicals used, etc. As discussed above, iron ores are also active in the hydrotreatment of lignin. Therefore, Idoia and co-workers studied the hydrotreatment of various extracted lignins from beech wood, wheat straw, pine wood, etc. as well as commercial lignins (Kraft, Soda) using limonite catalyst.^126^ The oil yield is in the range of 22–41% (Figure 10(A)). The highest yield of oil and monomers was obtained for Kraft M lignin (oil: ∼ 33%, monomers: 29%), followed by Kraft AT (monomers: 25.7%) and organosolv lignin from pine wood (25.3%). The product variations were directly related to the amount of *p*-hydroxyphenyl (H), guaiacyl (G) and syringyl (S) units and the corresponding linkages in the lignin polymer (^1^H–^13^C–HSQC NMR (Figure 10(B)). It is reported that lignins (Kraft, organosolv from pine) from softwood consisting of G units gave the best results in lignin hydrotreatment with the limonite catalyst. On the other hand, lignins from hardwood containing a mixture of G and S units (major) showed relatively lower performance than lignin from softwood.^127^ No significant amount of H units is observed in both softwood and hardwood lignins, which does not affect the overall monomer yield. The thermal stability of the S–G–H units also has a major influence on the monomer yield. Since the S units have a relatively lower thermal stability than the G units, the repolymerization reaction was accelerated and led to the formation of char in the case of hardwood lignins (Figure 10(C)). Furthermore, lignins with a high content of aromatics and a low content of methoxy groups lead to a suppression of the gasification reaction and to the formation of char. The content of aromatic structures is higher in lignins from softwood (Kraft, Soda) than in lignins from hardwood, which is due to the repolymerization reaction during the pulping process.^128^ It was also found that the sulfur-containing lignins perform better than the organosolv lignin. The molecular weight of the lignin has less influence than the type of bonds β–O–4, β–β, β–5 and S–G–H, etc. Limonite catalysts are best suited for processing of sulfur-containing lignins such as Kraft, as the sulfur content in the lignin is used to produce the catalyst in the active iron sulfide.

**Figure 11:**
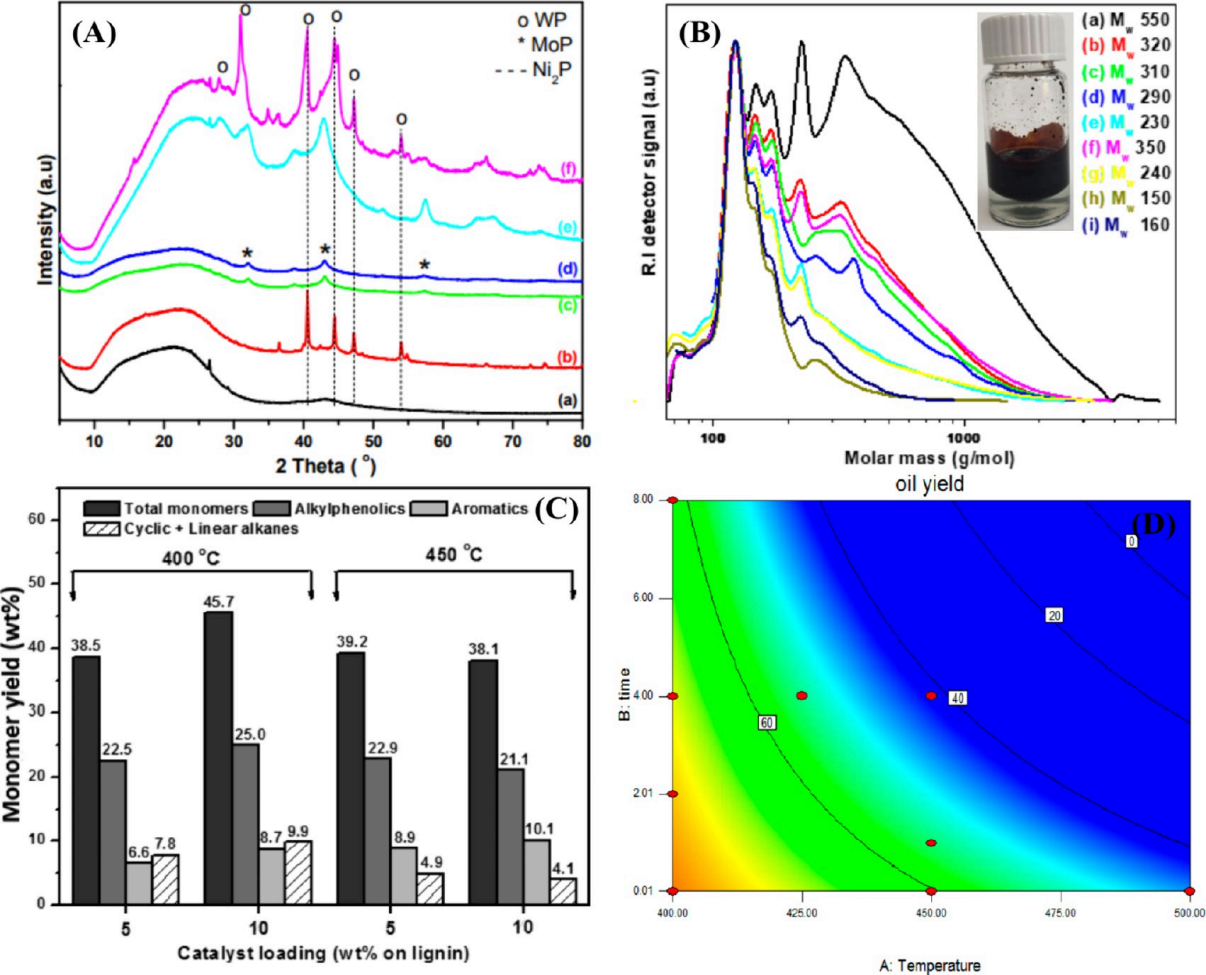
(A) XRD patterns of metal phosphides on AC ((a) AC, (b) 5NiP/AC, (c) 15MoP/AC, (d) 20NiMoP/AC, (e) 15WP/AC, (f) 20NiWP/AC), (B) gel permeation chromatograms of the lignin oils obtained using the 20NiMoP/AC catalyst at different temperatures and reaction times ((a) 400 °C–0 h, (b) 400 °C–2 h, (c) 400 °C–4 h, (d) 400 °C–8 h, (e) 425 °C–4 h, (f) 450 °C–0 h, (g) 450 °C–1 h, (h) 450 °C–4 h, and (i) 500 °C–0 h), (C) effect of catalyst loading (20 NiMoP/AC) and temperature on the monomer yield (wt % on lignin) and amounts of important product classes (wt % on lignin), and (D) lignin oil yield (wt% on lignin) versus temperature (°C) and reaction time (h).^133^

Metal phosphides are among the most active catalysts, and the activity of these catalysts is comparable to that of noble metal catalysts.^129,130^ Transition metal phosphide catalysts are generally used for heteroatom removal processes, such as hydrodesulfurization, hydrodenitrogenation, and hydroprocessing.^131,132^ Since Kraft lignin is composed of sulfur and noble metal catalysts are generally not used for hydroprocessing because they deactivate quickly. Therefore, metal phosphides are an alternative to noble metals. In our previous study, mono- and bimetallic phosphides of Ni, Mo, W on activated carbon were used for hydrotreatment of Kraft lignin in the temperature range of 400–500 °C (Figure 11(A)).^133^ The 5NiP/AC catalyst showed low activity compared to Mo– and W–containing catalysts. The promotion of Ni has a positive effect on Mo/W-based mono- and bimetallic phosphide catalysts (oil yield: 59.3–64.3% and monomer yield: 30–39.9%) and is higher in the case of Mo–based catalysts. The average molecular weight of the oils decreased with temperature, indicating that the monomer yield is proportional to the hydrotreatment temperature (Figure 11(B)). However, it is very important to select an optimum temperature to obtain a high yield of monomers with a low char. At 500 °C, the high char content was 19.8% for the MoP/AC catalyst and 15.2% for the NiMoP/AC catalyst. Considering the high oil yield (70.6%), the monomers (45.7%), and no/low char formation, the optimum conditions for hydrotreatment of Kraft lignin with the NiMoP/AC catalyst are 400 °C, 10% catalyst loading and 100 bar H_2_. In the hydrotreatment of lignin, the bimetallic metal phosphides are highly active and the monomer yield is comparable to that of noble metal catalysts (see Table 3). Moreover, a reaction network was proposed for the various parallel, consecutive reactions in the solid, liquid, and gaseous phases (*vide infra*). We also used a multivariable regression model to derive the relationship between lignin oil yield, reaction temperature and reaction time for the best catalyst, 20NiMoP/AC. The effect of the process variables (time and temperature) on the oil yield is shown in a contour plot (see Figure 11(D)).
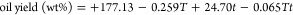
1

**Table 3:** Solvent-Free Hydrotreatment of Sulfur Containing Lignins over Heterogeneous Catalysts

S. no.	catalyst	reaction conditions	total monomers yield (%)^a^	major product: alkyl phenolics (%)	ref
1.	NiMo/Al_2_O_3_	350 °C, 100 bar H_2_ at RT, 4 h	14.8	10.5	(26)
2.	CoMo/Al_2_O_3_	350 °C, 100 bar H_2_ at RT, 4 h	10.8	7.1	(26)
3.	NiMo/ZSM–5	350 °C, 100 bar H_2_ at RT, 4 h	15.5	10.5	(26)
4.	CoMo/ZSM–5	350 °C, 100 bar H_2_ at RT, 4 h	18.2	12.3	(26)
5.	NiMo/AC	350 °C, 100 bar H_2_ at RT, 4 h	20.4	13.3	(26)
6.	CoMo/AC	350 °C, 100 bar H_2_ at RT, 4 h	21.5	12.6	(26)
7.	NiMo/MgO–La_2_O_3_	350 °C, 100 bar H_2_ at RT, 4 h	26.4	15.7	(26)
8.	CoMo/MgO–La_2_O_3_	350 °C, 100 bar H_2_ at RT, 4 h	18.1	12.5	(26)
9.	limonite	350 °C, 100 bar H_2_ at RT, 4 h	17.3	11.7	(125)
10.	limonite	400 °C, 100 bar H_2_ at RT, 4 h	17.5	12.1	(125)
11.	limonite	450 °C, 100 bar H_2_ at RT, 4 h	30.9	16.7	(125)
12.	geothite	450 °C, 100 bar H_2_ at RT, 4 h	23.5	14.6	(125)
13.	iron disulfide	450 °C, 100 bar H_2_ at RT, 4 h	23.6	15	(125)
14.	CoMo (commercial)	450 °C, 100 bar H_2_ at RT, 4 h	29.4	12	(125)
15.	limonite	450 °C, 100 bar H_2_ at RT, 4 h	25.7	14.5	(126)
16.	limonite	450 °C, 100 bar H_2_ at RT, 4 h	29.0	18	(126)
17.	limonite (soda)^b^	450 °C, 100 bar H_2_ at RT, 4 h	19.7	11.5	(126)
18.	limonite (Alcell)^b^	450 °C, 100 bar H_2_ at RT, 4 h	19.0	9.0	(126)
19.	limonite (organosolv-beachwood)^b^	450 °C, 100 bar H_2_ at RT, 4 h	15.7	10	(126)
20.	limonite (organosolv-wheat straw)^b^	450 °C, 100 bar H_2_ at RT, 4 h	15.9	8.5	(126)
21.	limonite (organosolv from pine)^b^	450 °C, 100 bar H_2_ at RT, 4 h	25.3	14.0	(126)
22.	limonite (organosolv from pine-PLC)^b^	450 °C, 100 bar H_2_ at RT, 4 h	22.9	12.5	(126)
23.	limonite (pyrolytic lignin)^b^	450 °C, 100 bar H_2_ at RT, 4 h	23.4	13	(126)
24.	5NiP/AC	400 °C, 100 bar H_2_ at RT, 4 h	13.7	9	(133)
25.	15MoP/AC	400 °C, 100 bar H_2_ at RT, 4 h	38.7	22.4	(133)
26.	15WP/AC	400 °C, 100 bar H_2_ at RT, 4 h	29.3	17.3	(133)
27.	20NiMoP/AC	400 °C, 100 bar H_2_ at RT, 4 h	39.9	22.5	(133)
28.	20NiWP/AC	400 °C, 100 bar H_2_ at RT, 4 h	30.0	17.9	(133)
29.	15MoP/AC	450 °C, 100 bar H_2_ at RT, 1 h	38.2	24.2	(133)
30.	20NiMoP/AC	450 °C, 100 bar H_2_ at RT, 1 h	39.2	22.9	(133)
31.	15MoP/AC	500 °C, 100 bar H_2_ at RT, 0 h	33.0	20.4	(133)
32.	20NiMoP/AC	500 °C, 100 bar H_2_ at RT, 0 h	36.6	22.6	(133)
33.	20NiMoP/AC^c^	400 °C, 100 bar H_2_ at RT, 2 h	45.7	25.0	(133)
34.	20NiMoP/AC^c^	450 °C, 100 bar H_2_ at RT, 1 h	38.1	21.1	(30)
35.	20NiMoP/TiO_2_^c^	400 °C, 100 bar H_2_ at RT, 2 h	40.6	25.3	(30)
36.	20NiMoP/SiO_2_–Al_2_O_3_^c^	400 °C, 100 bar H_2_ at RT, 2 h	31.2	19.1	(30)
37.	20NiMoP/SiO_2_^c^	400 °C, 100 bar H_2_ at RT, 2 h	51.8	30.6	(30)
38.	20NiMoP/MgO–La_2_O_3_^c^	400 °C, 100 bar H_2_ at RT, 2 h	34.7	20.6	(30)
39.	20NiMoP/AC^c^	400 °C, 100 bar H_2_ at RT, 2 h	43.0	28.2	(30)
40.	20NiMoP/SiO_2_^c^^d^	400 °C, 100 bar H_2_ at RT, 2 h	51.5	28.9	(30)
41.	20NiMoP/SiO_2_^c^^e^	400 °C, 100 bar H_2_ at RT, 2 h	34.4	16.2	(30)
42.	Ru/C	450 °C, 100 bar H_2_ at RT, 4 h	∼30.0	∼14.9	(31)
43.	Ru/Al_2_O_3_	450 °C, 100 bar H_2_ at RT, 4 h	∼ 25.0	∼14.8	(31)
44.	Pt/C	450 °C, 100 bar H_2_ at RT, 4 h	∼22.0	∼12	(31)
45.	Pt/Al_2_O_3_	450 °C, 100 bar H_2_ at RT, 4 h	∼33.0	∼18	(31)
46.	Pd/C	450 °C, 100 bar H_2_ at RT, 4 h	∼26.5	∼12.5	(31)
47.	Pd/Al_2_O_3_	450 °C, 100 bar H_2_ at RT, 4 h	∼31.0	∼17.5	(31)
48.	Rh/C	450 °C, 100 bar H_2_ at RT, 4 h	∼30.0	∼15.3	(31)
49.	Rh/Al_2_O_3_	450 °C, 100 bar H_2_ at RT, 4 h	∼34.0	∼17.5	(31)
50.	Ru/C (OS–W LPO)	400 °C, 100 bar H_2_ at RT, 4 h	48.6	27.9	(32)
51.	Ru/C (OS–P LPO)	400 °C, 100 bar H_2_ at RT, 4 h	41.4	16.9	(32)
52.	CoMo/Al_2_O_3_ (Kraft LPO–I)	400 °C, 100 bar H_2_ at RT, 4 h	28.8	21.2	(32)
53.	20NiMoP/AC (Kraft LPO–II)	350 °C, 100 bar H_2_ at RT, 2 h	54.9	30.7	(32)
54.	20NiMoP/AC (Kraft LPO–II)	400 °C, 100 bar H_2_ at RT, 2 h	81.9	44.3	(32)
55.	20NiMoP/AC (Kraft)	350 °C, 100 bar H_2_ at RT, 4 h	20.5	11.4	(32)
56.	20NiMoP/AC (Kraft)	400 °C, 100 bar H_2_ at RT, 2 h	38.5	22.5	(32)
57.	20NiMoP/AC (Kraft)	400 °C, 100 bar H_2_ at RT, 4 h	39.9	22.5	(32)
58.	NiMo/aluminosilica + 20% Cr_2_O_3_/alumina (Pine Kraft-Kaukas)	430 °C, 90 bar H_2_ at RT, 1 h	38.4	10.8 (aromatics)	(136)
				9.4 (alkylphenolics)	
59.	NiMo/aluminosilica + 20% Cr_2_O_3_/alumina (Birch Kraft-Kaukas)	400 °C, 100 bar H_2_ at RT, 0.5 h	13.9	5.9 (aromatics)	(136)
				5.0 (alkylphenolics)	
60.	NiMo/zeolite (Pine Kraft-Holmen)	395 °C, 90 bar H_2_ at RT, 0.33 h	17.0	5.6 (aromatics)	(136)
				4.8 (alkylphenolics)	
61.	NiMo/zeolite + 20%Cr_2_O_3_/alumina (Birch Kraft-Kaukas)	395 °C, 100 bar H_2_ at RT, 0.6 h	15.5	5.6 (aromatics)	(136)
				4.7 (alkylphenolics)	
62.	NiMo/aluminosilica +20%Cr_2_O_3_/alumina (Organocell)	395 °C, 100 bar H_2_ at RT, 0.5 h	19.1	10.5 (aromatics)	(136)
				8.9 (alkylphenolics)	
63.	NiMoP/SiO_2_ (unmilled)	400 °C, 100 bar H_2_ at RT, 2 h	13.7	∼22.5	(33)
64.	NiMoP/SiO_2_ (ball milled–5 min)	400 °C, 100 bar H_2_ at RT, 2 h	14.7	∼23.9	(33)
65.	NiMoP/SiO_2_ (ball milled–10 min)	400 °C, 100 bar H_2_ at RT, 2 h	17.6	∼29.8	(33)
66.	NiMoP/SiO_2_ (ball milled–36 min)	400 °C, 100 bar H_2_ at RT, 2 h	15.6	∼25.4	(33)
67.	NiMoP/SiO_2_ (ball milled–60 min)	400 °C, 100 bar H_2_ at RT, 2 h	15.2	∼23.5	(33)

a total monomers: alkylphenolics + oxygen-free aromatics + guaiacols + catechols + liner/branched alkanes + cyclic alkanes + ketones + alcohols.

b sulfur-free lignin.

c 10% catalyst loading on lignin intake.

d lignoboost

e Alcell, LPO-I-lignin pyrolysis oil obtained at 450 °C, LPO-II- lignin pyrolysis oil obtained at 550 °C.

**Figure 12:**
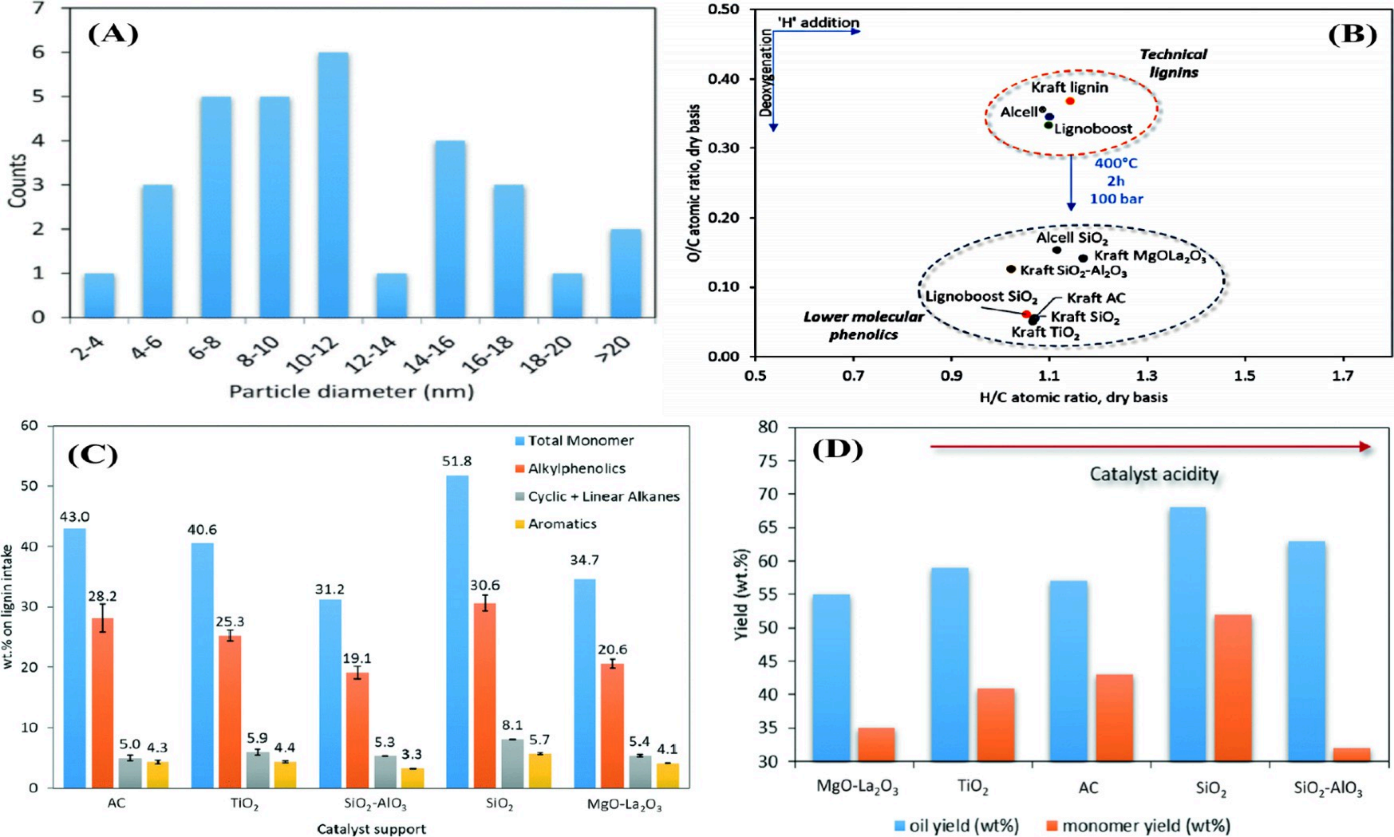
(A) Particle size distribution of the NiMoP/AC catalyst. (B) Van–Krevelen plot for the lignin oil obtained over various catalysts, (C) monomer yields obtained for various supported catalysts, (D) correlation of acidity with the oil and monomer yields for the hydrotreatment of Kraft lignin over NiMoP dispersed on different supports. (A–D) Reproduced with permission from ref (30). Copyright 2021 RSC Publications.

Since the 20NiMoP/AC catalyst showed promising results and in continuation of the detailed study on metal phosphides, Jessi et al. investigated NiMoP on TiO_2_, SiO_2_, SiO_2_/Al_2_O_3_, MgO–L_2_O_3_, etc., to study the effect of acidic and basic supports in the hydrotreatment of Kraft lignin.^30^ In the case of SiO_2_-containing supports, high lignin oil yields were obtained compared to other catalysts. The oil yields are in the following order 20NiMoP/SiO_2_ > 20NiMoP/SiO_2_–Al_2_O_3_ > 20NiMoP/TiO_2_ > 20NiMoP/AC > 20NiMoP/MgO–La_2_O_3_. The oxygen content in the hydrotreated Kraft lignin is significantly reduced compared to the parent lignin (Figure 12(B)). However, the monomer yield for the different catalysts is in the following order: 20NiMoP/SiO_2_ (51.8%) > 20NiMoP/AC (43.0%) > 20NiMoP/TiO_2_ (40.6%) > 20NiMoP/MgO–La_2_O_3_ (34.7%) > 20NiMoP/SiO_2_–Al_2_O_3_ (31.2%) (see Figure 12(C)). For all catalysts, the main product consists of alkylphenolics, and the yield of aromatics reached a maximum of 8.1% in the case of 20NiMoP/SiO_2_. The high acidity of the catalysts promotes the repolymerization of oligomers and leads to the formation of char.^92^ Therefore, a moderate acidity and small particle size are best suited to achieve a high yield of monomers (see Figure 12(A,D)). The analysis of the used catalysts shows that the sulfur present in the lignin reacts with the metal phosphide and leads to the formation of MoS_2_ and Ni_7_S_6_ species. The high sulfur content was observed in the case of the used NiMoP/MgO–La_2_O_3_ catalyst. However, the conversion of metal phosphide to metal sulfides cannot be considered as deactivation of the catalyst, since metal sulfides are active in hydrodeoxygenation reactions, as mentioned above.^26,125^ The NiMoP/SiO_2_ catalyst gave promising results for other lignins such as Lignoboost (refined Kraft lignin) and Alcell lignins indicating the efficiency of the catalyst. However, Alcell lignin gave relatively lower oil and monomer yields than Kraft and lignoboost lignin, which could be due to the absence of sulfur in Alcell lignin. In sulfur containing lignins (Kraft and Lignoboost), the metal phosphides converted to metal sulfides, and both phases are active in the hydrodeoxygenation reaction, which could lead to high monomer yields.

As mentioned in the previous section, noble metal catalysts for sulfur-free lignins (Organosolv/Alcell) have been reported. Yang et al. used noble metal catalysts for hydrotreatment of Kraft lignin in isopropanol medium.^93^ However, the major drawback of the solvent-based technique is the production of oxygen-enriched (due to the use of isopropanol) and overhydrogenated monomers. Interestingly, Hita et al. used supported noble metal catalysts (Ru, Pt, Pd, and Rh on alumina and activated carbon) for the hydrotreatment of Kraft lignin under solvent-free conditions.^31^ Regardless of the type of noble metal, the alumina supported catalysts exhibited relatively higher activity than the carbon support, with the exception of Ru. The high activity of the alumina supported catalysts is due to the presence of mesopores and acidic sites compared to activated carbon, which favors the cleavage of the carbon chain.^134^ Among the noble metal catalysts studied, Rh/Al_2_O_3_ gave the best results (oil yield: 36.3%, monomer yield: 30%). The regeneration of the Rh/Al_2_O_3_ catalyst showed a drastic decrease in catalytic activity, mainly due to the presence of metal sulfides and factors such as surface area reduction, a low amount of metallic, acidic sites in the used catalyst. Therefore, the activity of noble metal catalysts is comparable to that of Mo-based metal sulfide/phosphide catalysts.^125,133^ To efficiently remove the heteroatom with metal sulfides, the metal–sulfur binding energies must be taken into account. This is because the metal–sulfur bond energy plays an important role in the bond making and bond breaking steps. Daudin et al. studied the effects of binding energies on the removal of sulfur from gasoline.^135^ According to their study, the binding energies should be moderate for effective removal of the heteroatom (S), and the Mo–based sulfides showed better performance than the noble metal catalysts. The same could be implemented for the removal of oxygen, i.e., HDO, which is why low activity was observed for the noble metal catalysts. Moreover, no significant difference was found in the average molecular weights (GPC analysis) of lignin oils from fresh and regenerated Rh/Al_2_O_3_ catalysts, indicating that depolymerization of Kraft lignin occurred to the same extent in both cases. However, a drastic difference in monomer yield was observed, further emphasizing the decreasing ability of the catalysts to remove oxygen. When comparing the monomer yield of metal sulfides, metal phosphides and noble metal catalysts, it becomes clear that the effect of support has a significant influence. In the case of metal sulfides, neutral or basic supports are best suited, while acidic supports are also best suited for metal phosphides and noble metal catalysts in order to achieve high monomer yields. This could be due to the high spillover of hydrogen on noble metals/metal phosphides than on metal sulfides, which accelerates the depolymerization rather than the condensation reaction.

In another study, a two-step thermochemical approach was used for the production of monomers from Kraft, soda, and organosolv lignins from spruce (OS–S), poplar (OS–P) and wheat straw (OS–W) etc. using Ru/C, NiMoP/AC, and CoMo/Al_2_O_3_ catalysts.^32^ In order to achieve a high yield of aromatics/alkylphenolics, the authors compared the monomer yield from the pyrolysis process with the two-step approach (pyrolysis followed by HDO) and direct HDO. The one-step pyrolysis (at 450 °C) of various lignins resulted in a very low yield of monomers, as follows: Kraft (13.8%), OS–W (12.4%), OS–P (11.8%), OS–S (10.6%). The monomer yield was enhanced during pyrolysis at 550 °C (17.8%) and was highest for Kraft lignin (Kraft LPO–II). In the case of pyrolysis, the main components are guaiacolics, alkylphenolics, and catechols. In contrast, the two-step (pyrolysis–HDO) and single-step HDO mainly yield alkylphenolics. Based on lignin uptake and comparison of monomer yields, the 20NiMoP/AC catalyst gave the best results for single-step HDO (30.5%) at 400 °C, while the two-step approach gave only 15.4%. A similar trend was observed when the reaction was carried out at 350 °C. However, the monomer yield was 9.9% with pyrolysis–HDO and 14.3% with direct HDO. Direct pyrolysis of Kraft lignin gave 12.8% of total aromatics, which is better than the two-step approach at 350 °C. Therefore, single-step HDO at 400 °C is best approach to achieve high monomer yields, and single-step pyrolysis is best at 350 °C. It is known that the proportion of char in pyrolysis is high compared to HDO. Therefore, single-step HDO is best the approach for the production of monomers.

In an early report, Oasmaa et al. investigated the hydrotreatment of Kraft lignins from various sources and compared them with the organocell lignin using NiMo/aluminosilica, NiMo/zeolite and 20%Cr_2_O_3_/alumina catalysts (see Table 3 (entry no. 58–62)).^136^ The oil yield obtained is in the range of 49–71% and is high in the case of the organocell lignin. However, the products detectable by GC are high for Kraft lignin (38.4%). Organocell lignin, on the other hand, gave a moderate yield (19.1%). The difference in the yields of oil and monomers is related to the lignin structure of hardwood and softwood. In all individual cases, the yield of oxygen-free aromatics and alkylphenolics is almost the same. However, the yield of aromatic compounds is slightly higher. It can be observed that the yield of GC-detectable monomers increases with increasing reaction temperature and is higher at 430 °C. However, it is difficult to conclude from this report which catalyst and type of lignin is best suited to achieve a high monomer yield, as the authors used different reaction conditions.

Ball milling of wood has been reported to result in minimal structural changes (compared to chemical extraction) and a decrease in molecular weight due to the cleavage of ether bonds on the six membered aromatic rings.^137,138^ Maja et al. reported that polyesterification of lignin with ε-caprolactone is stronger with ball–milled lignin than with unground lignin due to the small particle size and large surface area.^139^ In our previous study, these concepts (breaking the β–O–4 bonds and reducing the lignin particle size by mechanical ball milling) were applied to Kraft lignin followed by hydrotreatment, resulting in high yield of monomers using the NiMoP/SiO_2_ catalyst.^33^ Ball milling has a great impact on the particle size of lignin, as tiny particles were observed in the milled lignin, which has a positive effect on lignin oil recovery and monomers. The total monomer yields for unmilled and milled lignin was 22.5% and 29.8%, respectively (based on oil uptake). The improved depolymerization of ball–milled lignin was due to the improved heat transfer, which enable better contact between lignin and catalyst particles. As described in the previous section, fractionation of lignin and subsequent hydrotreatment also resulted in a high yield of monomers compared to the parent lignin.^118^ From the analysis of the lignin fractions, the presence of chemical bonds and their properties can be deduced so that, depending on the degree of difficulty, a suitable catalyst can be used for efficient depolymerization to achieve a high yield of monomers. The combination of the above two strategies could open new routes, such as milling and fractionation in the first step, followed by hydrotreatment, which could lead to low char and high monomer yield.

A summary of all the above catalytic systems for hydrotreatment of sulfur-containing lignins can be found in Table 3. In the case of sulfur-containing lignins, the most studied catalysts are metal sulfides and metal phosphides. The noble metal catalysts have also been reported for sulfur-containing lignins. The sulfur present in lignin has positive and negative effects on the monomer yield. In the case of metal sulfides and phosphides, the sulfur content in the lignin has a positive effect on lignin hydrotreatment. Whereas, noble metal catalysts, on the other hand, are deactivated during reuse due to the formation of metal sulfides. Direct HDO and ball milling effects also have a positive influence on the overall monomer yields

#### 5.1.3 Pyrolytic Lignin Hydrotreatment

There are two main processes for obtaining a liquid product from biomass: HTL and fast pyrolysis. A brief description of the HTL and pyrolysis processes has already been given in section 3. The main difference between these two techniques is that the HTL process treats wet biomass at moderate temperatures (200–400 °C), while the pyrolysis uses dry biomass at elevated temperatures (450–600 °C).^140^ Fast pyrolysis is another effective method for converting woody biomass (including grass, bagasse, forest residues and other biomass mixtures) into liquid products (pyrolysis oil) through a thermochemical process in the absence of oxygen/air with the main disadvantage of a high percentage of char formation.^141^ Pyrolysis oil is a brown, viscous liquid consisting of oxygenated (20–40%) chemicals. The composition of pyrolysis oil is uniform and has a higher energy density than that of lignocellulosic biomass. However, due to its high water content (15–30%), reactive oxygenated components, pH, and corrosiveness, pyrolysis oil cannot be used directly as a transport fuel.^142^ Due to the acidic nature of pyrolysis oil and the presence of compounds such as aldehydes and ketones, stability is compromised, i.e., undesirable repolymerization reactions and the fractionation of pyrolysis lead to a significant amount of char formation.^143^ Therefore, the stabilization of pyrolysis oil could be achieved by removing reactive components such as aldehydes and ketones (oxygenated compounds), i.e., by hydrotreatment/hydrodeoxygenation of pyrolysis oil. The hydrotreating process is generally carried out in the range of 300–500 °C and 100–200 bar hydrogen to reduce the oxygen content down to 10% to produce fuel-grade bio-oil and/or process it in an FCC reactor together with conventional refinery streams.^144,145^ Since pyrolysis oil is a complex mixture of several components, such as monomers and oligomers of cellulose, hemicellulose, and lignin etc., it is suitable for the production of aromatics from pyrolysis oil. There are two ways to produce aromatics from pyrolysis oil, namely, (i) direct hydrotreatment of the pyrolysis oil, (ii) separation of the lignin fraction from the pyrolysis oil followed by hydrotreatment of the pyrolytic lignin (*vide infra*). As this review article focuses on the conversion of lignin into valuable chemicals, the following section deals with the hydrotreatment of lignin from pyrolysis.

Compared to native or extracted lignins, pyrolytic lignins differ significantly in terms of their chemical structure and average molecular weight, as pyrolytic lignins are obtained from already processed woody biomass at high temperatures (450–600 °C). Therefore, the average molecular weight of pyrolytic lignins is in the range of 560–840 Da, which is very low compared to technical lignins (1600–2000 Da for Alcell, 4800–5200 Da (bagasse lignin)).^146,147^ The chemical bonds in pyrolytic lignins also differ from those in native lignins. The most common bonds in pyrolytic lignin are C–C (phenylcoumarin (β–1), resinol (β–β) and stilbene, diphenyl (5–5) etc.). The use of pyrolytic lignin instead of lignin has the additional advantage that the *M*_w_ is very low, which promotes high monomer yield. However, there are only a few research articles reporting on the catalytic hydrotreatment of pyrolytic lignins under solvent-free conditions. In this context, this section is dedicated to the solvent-free catalytic hydrotreatment of pyrolytic lignins from different wood sources.

**Figure 13:**
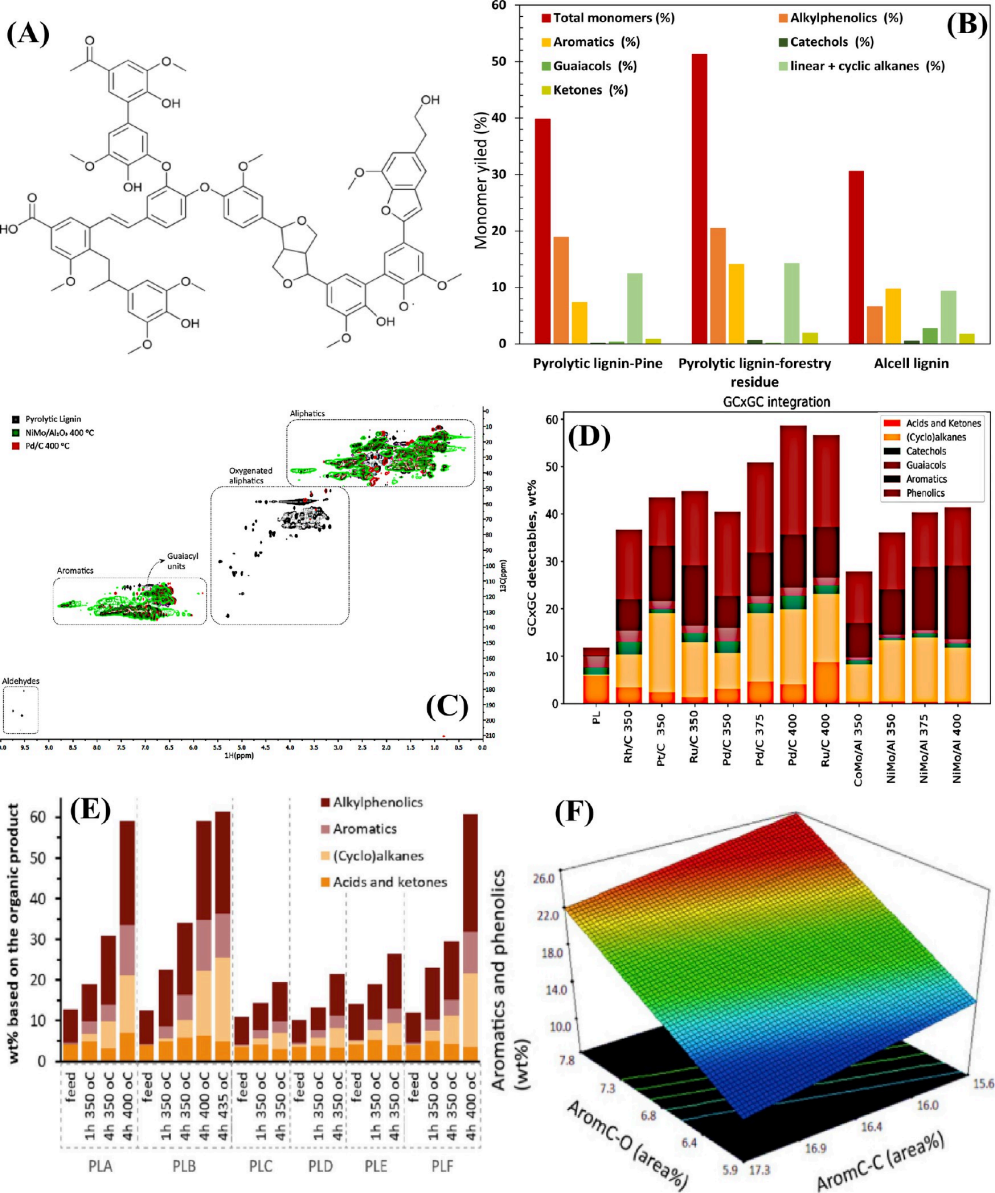
(A) A model structure of pyrolytic lignin. Reproduced with permission from ref (147). Copyright 2009 Elsevier. (B) Monomer yields from pyrolytic lignins from pine and forestry residue and Alcell lignin (data obtained from ref (146)). (C) HSQC spectra of pyrolytic lignin and hydrotreated pyrolytic lignins over NiMo/Al_2_O_3_ and Pd/C catalysts, (D) monomer yields over supported noble metal and NiMo/CoMo catalysts. (C,D) reproduced with permission from ref (34). Copyright 2019 Elsevier. (E) Monomer yields from the hydrotreated pyrolytic lignins from pine (PLA), commercial pine (PLB), pruning (PLC), verge grass (PLD), miscanthus (PLE), sunflower seed peel (PLF), etc., (F) statistical model surface plot for the alkylphenolics/aromatic yields versus chemical linkages in the PL feed. E and F reproduced with permission from ref (151). Copyright 2020 Elsevier.

Arjan et al. performed catalytic hydrotreatment of pyrolytic lignins derived from the pyrolysis of FR and pine wood using a 5% Ru/C catalyst.^146^ A proposed model structure for pyrolytic lignin is shown in Figure 13(A).^147^ The main product observed in the hydrotreated pyrolytic lignins (FR and Pine) were alkylphenolics as also observed for the lignins discussed in the previous sections. The overall monomer yield is highest (51.3%) for FR (Figure 13(B)). For the pyrolytic lignins from pine wood, the total monomer yield is 39.8%. These values are higher than the monomer yield of hydrotreated Alcell lignin (30.6%). The pyrolytic lignin from FR gave 3–times higher yield of alkylphenolics than the Alcell lignin. The high monomer yield for pyrolytic lignin is not surprising, as these lignins are derived (i.e., thermally treated) from the pyrolysis oils and have a low average molecular weight, which in turn leads to a lower *M*_w_ of the hydrotreated pyrolytic lignins. In addition, the ^13^C NMR spectra showed that ether linkages (α, β, γ: chemical shift range 58–100 ppm) are absent in pyrolytic lignin and are present to a significant extent in the case of Alcell lignin. Therefore, hydrotreatment of pyrolytic lignin is more advantageous for the production of aromatics than that of lignins. However, when comparing the monomers from the direct hydrotreatment of lignin and pyrolytic lignin, the overall production costs and energy efficiency must be taken into account, as pyrolytic lignin has undergone a thermal treatment i.e., pyrolysis. Regarding catalysis, in the case of pyrolytic lignin, noble metal catalysts are highly active than metal sulfides, as observed for simple lignins. A similar observation was made in the hydrotreatment of commercial pyrolytic lignins with noble metal catalysts, as reported by Figueiredo et al.^34^ In this study, the hydrotreatment of commercial pyrolytic lignin over noble metal catalysts (Ru, Pt, Pd, Rh on carbon) was investigated and compared with commercial HDS catalysts (sulfided NiMo, CoMo on alumina). Under similar reaction conditions, the oxygen content in the product oil decreased significantly (O/C ratio: 0.2–0.4), but the hydrogen content in the product oils varied for noble metals (H/C ratio: 1.35–1.52) and conventional HDS catalysts (H/C ratio: 1.15–1.38). This is due to the fact that noble metal catalysts are known for hydrogenation reactions and Ni(Co)–promoted Mo catalysts are known for heteroatom removal.^148,149^ This is evidenced by the comparison of aromatic yields for conventional hydrotreating catalysts with noble metal catalysts (see Figure 13(D)). In addition to the decreasing H/C ratio, the NiMo/Al_2_O_3_ catalyst produces aromatic rings from aliphatics (dehydrogenation reaction), which is a common fact observed in the hydrotreatment of heavy oils.^150^ The highest monomer yield (59%) was observed for Pd/C, with alkylphenolics and aromatics accounting for 35% (Figure 13(D)). These results are also confirmed by ^13^C and HSQC NMR, as the signals corresponding to the oxygenated aliphatic region disappeared in the hydrotreated pyrolytic lignin, while simultaneously the signal population in the aromatic and aliphatic regions increased (Figure 13(C)). The authors also performed PCA of the products, which revealed that 83.2% of the total variance contributing from PC1 (52.6%) and PC2 (30.6%). PC1 depends on the structural properties of the oils (such as HSQC, O and H content) and PC2 describes the extent of hydrocracking (average *M*_w_, monomer yield and residue). The PCA analysis shows that temperature has a strong influence on depolymerization, as a point trend was observed at different temperatures. Compared to simple lignins, pyrolytic lignin resulted in a high yield of aromatics. Therefore, the hydrotreatment of PL is one of the most attractive strategies and should have a positive impact on pilot-scale studies and techno-economic evaluation.

As mentioned above, pyrolysis oil showed the best results in catalytic hydrotreatment than simple lignins over a Pd/C catalyst.^34^ In order to derive a general statistical model to determine the relationship between the different feedstock properties and product yield, Figueiredo et al. investigated the hydrotreatment of different types of pyrolytic lignins (extracted from the pyrolysis oils of pine (PLA), commercial pine (PLB), prunings (PLC), verge grass (PLD), miscanthus (PLE), sunflower seed peel (PLF), etc.) over Pd/C catalyst.^151^ The reactions performed at 350 °C resulted in a more viscous oil with high an oxygen content than oils obtained at 400 °C. The optimum temperature for the production of aromatics is 400 °C and was found in the case of PL (PLA and PLB) with a total monomer yield of about 60% (Figure 13(E)). Based on the lignin feed, the monomer yield for PLA, PLB and PLF was 39.1%, 38.8%, and 37.4%, respectively. PCA analysis of the lignin oils also showed clear trends and clusters formed by two major components, viz., PC (171.7%) and PC2 (17.7%). However, the properties of the hydrotreated PLs differed from those of the original PLs. The score plot showed that the reaction temperature has a significant influence on the product properties, while the reaction time has the least effect. Furthermore, the authors proposed a linear model representing the relationship between the PL properties and the aromatics yield (see eq 2). The statistical model surface for the dependence of the yield of alkylphenolics/aromatic on the chemical linkages in the PL feedstock is shown in Figure 13(F).
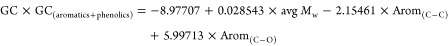
2

All the above catalytic systems used for the depolymerization of pyrolytic lignin are listed in Table 4. Noble metal and conventional hydrotreatment catalysts were used in the depolymerization of pyrolytic lignin. Since the pyrolytic lignins are sulfur-free, noble metal catalysts are best suited for the production of valuable products. Compared to simple/parent lignins, pyrolytic lignins lead to a high yield of monomers. However, the production of aromatics from pyrolytic lignins involves two steps: i. pyrolysis of wood and extraction of lignin, ii. hydrotreatment of pyrolytic lignin. Therefore, it is very important to compare the overall production when considering a pilot/industrial scale pyrolytic hydrotreatment process.

**Table 4:** Hydrotreatment of Pyrolytic Lignins over Heterogeneous Catalysts

S. no.	source	catalyst	reaction conditions	total monomers yield (%)^a^	major product alkylphenolics (%)	ref
1.	pyrolytic lignin from pine wood	Ru/C	400 °C, 100 bar H_2_ at RT, 4 h	39.8%	18.9	(146)
2.	pyrolytic lignin from forestry residue	Ru/C	400 °C, 100 bar H_2_ at RT, 4 h	51.3	20.5	(146)
3.	pyrolytic lignin	-	350 °C, 100 bar H_2_ at RT, 4 h	∼11.5	∼1.6	(34)
4.	pyrolytic lignin	Rh/C	350 °C, 100 bar H_2_ at RT, 4 h	∼36.5%	∼16.9	(34)
5.	pyrolytic lignin	Pt/C	350 °C, 100 bar H_2_ at RT, 4 h	∼43%	∼10.8	(34)
6.	pyrolytic lignin	Ru/C	350 °C, 100 bar H_2_ at RT, 4 h	∼44.5%	∼16.9	(34)
7.	pyrolytic lignin	Pd/C	350 °C, 100 bar H_2_ at RT, 4 h	∼40%	∼20.0	(151)
8.	pyrolytic lignin	Pd/C	375 °C, 100 bar H_2_ at RT, 4 h	∼50.5%	∼20.8	(151)
9.	pyrolytic lignin	Pd/C	400 °C, 100 bar H_2_ at RT, 4 h	∼59%	∼23.4	(151)
10.	pyrolytic lignin	Ru/C	400 °C, 100 bar H_2_ at RT, 4 h	∼56.5%	∼21.5	(151)
11.	pyrolytic lignin	CoMo/Al_2_O_3_	350 °C, 100 bar H_2_ at RT, 4 h	∼27%	∼12.3	(151)
12.	pyrolytic lignin	NiMo/Al_2_O_3_	350 °C, 100 bar H_2_ at RT, 4 h	∼35%	∼13.1	(151)
13.	pyrolytic lignin	NiMo/Al_2_O_3_	375 °C, 100 bar H_2_ at RT, 4 h	∼39.5%	∼12.3	(151)
14.	pyrolytic lignin	NiMo/Al_2_O_3_	400 °C, 100 bar H_2_ at RT, 4 h	∼40.5%	∼13.9	(151)
15.	PLA	-	350 °C, 100 bar H_2_ at RT, 1 h	∼12.6%^b^	∼8.2	(151)
16.	PLA	Pd/C	350 °C, 100 bar H_2_ at RT, 1 h	∼18.6%^b^	∼8.6	(151)
17.	PLA	Pd/C	350 °C, 100 bar H_2_ at RT, 4 h	∼30.7%^b^	∼16.8	(151)
18.	PLA	Pd/C	400 °C, 100 bar H_2_ at RT, 4 h	∼58.5%^b^	∼24.6	(151)
19.	PLB	-	350 °C, 100 bar H_2_ at RT, 1 h	∼12.1%^b^	∼7.8	(151)
20.	PLB	Pd/C	350 °C, 100 bar H_2_ at RT, 1 h	∼22.8%^b^	∼13.6	(151)
21.	PLB	Pd/C	350 °C, 100 bar H_2_ at RT, 4 h	∼33.9%^b^	∼16.4	(151)
22.	PLB	Pd/C	400 °C, 100 bar H_2_ at RT, 4 h	∼58.5%^b^	∼23.6	(151)
23.	PLB	Pd/C	435 °C, 100 bar H_2_ at RT, 4 h	∼60.4%^b^	∼24.3	(151)
24.	PLC	-	350 °C, 100 bar H_2_ at RT, 1 h	∼10.7%^b^	∼7.8	(151)
25.	PLC	Pd/C	350 °C, 100 bar H_2_ at RT, 1 h	∼14.3%^b^	∼6.4	(151)
26.	PLC	Pd/C	350 °C, 100 bar H_2_ at RT, 4 h	∼18.8%^b^	∼9.7	(151)
27.	PLD	-	350 °C, 100 bar H_2_ at RT, 1 h	∼10%^b^	∼5.4	(151)
28.	PLD	Pd/C	350 °C, 100 bar H_2_ at RT, 1 h	∼12.8%^b^	∼5.7	(151)
29.	PLD	Pd/C	350 °C, 100 bar H_2_ at RT, 4 h	∼21.4%^b^	∼10	(151)
30.	PLE	-	350 °C, 100 bar H_2_ at RT, 1 h	∼14%^b^	∼8.6	(151)
31.	PLE	Pd/C	350 °C, 100 bar H_2_ at RT, 1 h	∼18.6%^b^	∼8.6	(151)
32.	PLE	Pd/C	350 °C, 100 bar H_2_ at RT, 4 h	∼25.7%^b^	∼12.8	(151)
33.	PLF	-	350 °C, 100 bar H_2_ at RT, 1 h	∼11.8%^b^	∼7.1	(151)
34.	PLF	Pd/C	350 °C, 100 bar H_2_ at RT, 1 h	∼22.8%^b^	∼12.8	(151)
35.	PLF	Pd/C	350 °C, 100 bar H_2_ at RT, 4 h	∼29.3%^b^	∼14	(151)
36.	PLF	Pd/C	400 °C, 100 bar H_2_ at RT, 4 h	∼60%^b^	∼28.6	(151)

a Total monomers: alkylphenolics + oxygen-free aromatics + guaiacols + catechols + liner/branched alkanes + cyclic alkanes + ketones + alcohols.

b Yields are based on organic phase.

### 5.2 Continuous Catalytic Hydrotreatment of Lignin

In most research reports, batch reactors are used for the solvent-free catalytic hydrotreatment of lignin. Continuous catalytic experiments for solvent-free depolymerization of lignin are limited and few reports can be found in the literature. The major drawback of solvent-free depolymerization is the solid form of the lignin, which makes continuous pumping into the reactor difficult. However, there are very few reports of continuous reactor systems in which lignin is fed into the reactor in a solid state or in lignin oils. The operating conditions for the reaction are 375–450 °C and 40–180 bar hydrogen pressure. This review article is about solvent-free catalytic hydrotreatment, but mixing lignin with lignin or slurry oil is also considered as solvent-free hydrotreatment. Since the product selectivity does not change after the hydrotreatment of lignin, as is the case with protic solvents such as methanol, ethanol, etc. as mentioned earlier.

**Figure 14:**
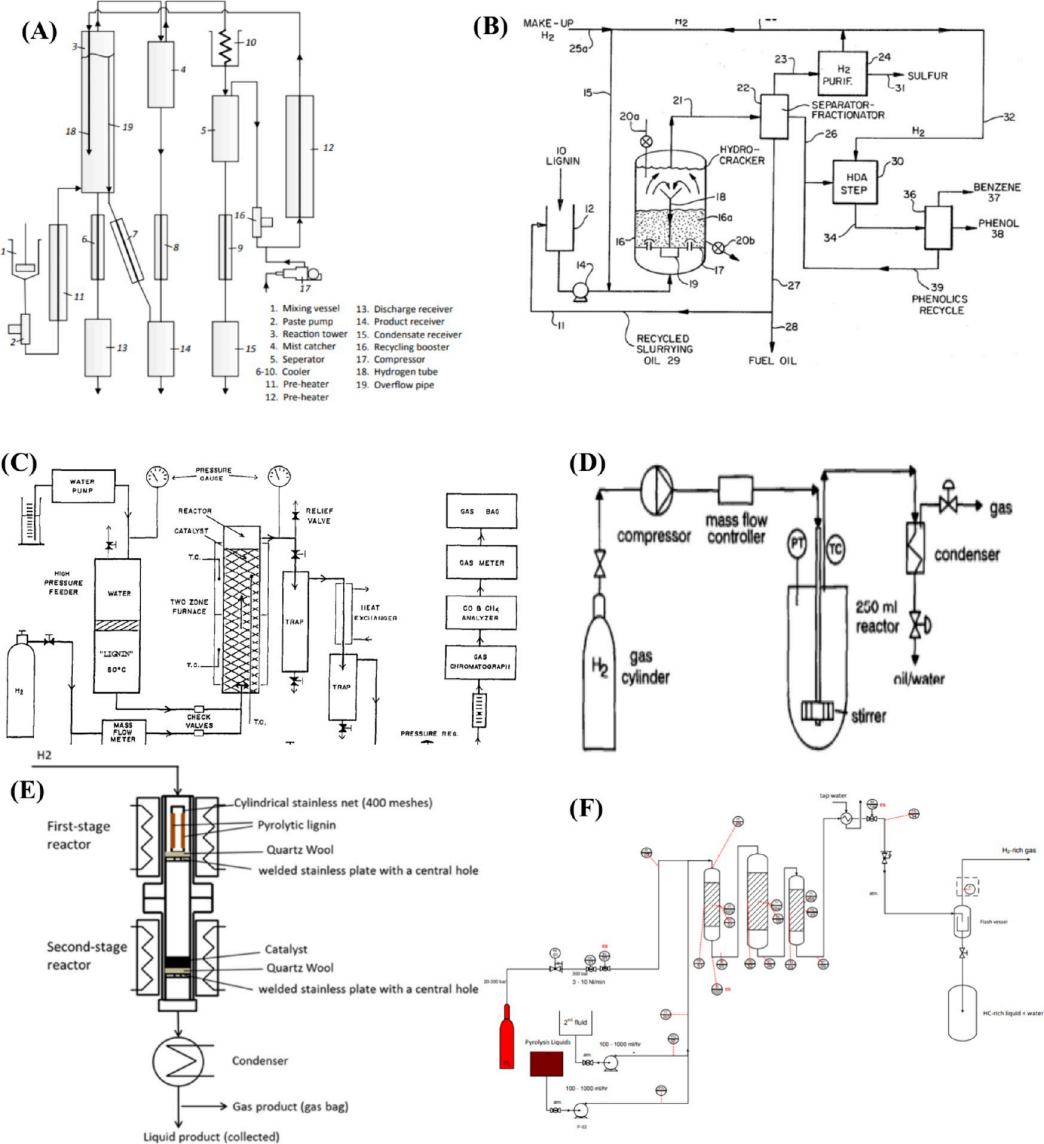
(A) Schematic diagram of the continuous reactor for the lignin liquefaction,^152,159^ (B) reactor design for hydrocracking of Kraft lignin,^153^ (C) continuous hydrogenation and hydrodeoxygenation reactor,^154^ (D) semicontinuous reactor for the hydropyrolysis of lignin. Reproduced with permission from ref (155). Copyright 1993 Elsevier. (E) Dual stage reactor for hydrocracking and cracking. Reproduced with permission from ref (157). Copyright 2023 Elsevier. (F) Continuous reaction setup for the hydrotreatment of pyrolysis oil. Reproduced with permission from ref (158). Copyright 2016 RSC Publications.

One of the earlier patents on liquefaction of lignin was published by Motoyoshi et al. from the Noguchi Institute in Japan, where different types of lignin were used for continuous liquefaction using the heterogeneous catalyst, FeS along with cocatalysts such as sulfided forms of Cu, Ag, Sn, Co, Ni, Zn, Mo, etc.^152^ For the catalytic experiments, the authors mixed lignin in one or more of the following proportion of about 20–40%: Lignin tar, tetralin, phenols, gas oil, creosote oil, coal oil, and water. The reaction conditions for the continuous catalytic depolymerization of lignin were 350–400 °C, up to 220 bar hydrogen and 1–2 h. A schematic diagram of the reactor setup is shown in Figure 14(A). After the reaction, two layers of the liquid product were observed (aqueous and organic). The aqueous layer consists of acetone and methanol. The organic layer on the other hand, contians monophenols, catechols and heavy oil, etc. The various organic components contained in the organic layer were separated by distillation. From the list of cocatalysts used in this study, the Cu catalyst prevents the formation of hydrocarbons. The yield of monophenols was about 51% and the main components of the mixture are p-cresol (20.4 parts), 4-ethylphenol (15.6 parts), o-cresol (7 parts) and 4-propylphenol (4 parts).

In the later patent, Huibers et al. developed the hydrotreatment of Kraft lignin in a continuous reactor to produce phenol and benzene.^153^ The schematic diagram of the ebullated hydrocracking reactor is shown in Figure 14(B). In this process, the lignin was first mixed with the process-derived oil and used for hydrocracking. The aromatic fuel oil and hydrogen gas were separated from the hydrocracking products, and some of the aromatic fuel oil, called slurry oil, was recycled. During the hydrocracking reaction, the bond cleavage leads to the formation of free radicals, which were stabilized by the hydrogen in the slurry oil. To obtain phenol and benzene, the monoaromatic portion or the heavy alkylated aromatics were hydrodealkylated. The reaction conditions for the hydrocracking of lignin were 340–460 °C, 34–172 bar hydrogen and space velocity 1–10 pounds lignin h^–1^ pound catalyst^–1^. The catalyst consists of a combination of active components (Fe, Mo, Co, Ni) and supports (alumina, silica). The authors stated that the production of alkylphenolics was about 37.5%. The detailed composition of alkylphenolics is as follows: *p*-propylphenol (20%), *p*-ethylphenol (33.2%), *m*-cresol (11.9%), p-cresol (9.7%), 2,4-xylenol (7%), phenol (6.6%). In addition, the authors claimed that 60 mol % of the cresols were converted to phenol in the second stage of hydrodealkylation, for which a low temperature and a high residence time are suggested. The main advantage of this process over the Noguchi process is that the formation of gaseous and heavy oils is relatively low.

In the two reports mentioned above, the lignin is mixed with lignin tar or lignin derived oil, etc. Interestingly, Piskorz et al. studied the hydrotreatment of pyrolytic lignin (from fast pyrolysis of hog fuel) in a continuous fixed-bed reactor (see Figure 14(C)) without the use of solvent.^154^ For the continuous feed, pyrolytic lignin was preheated to liquefaction and pumped in at a flow rate of 0.51–1 g_lignin_ g_cat_^–1^ h^–1^ (upward flow). The reaction parameters used for lignin upgrading were 140 bar H_2_, 400–415 °C, CoMo catalyst. The obtained liquid fraction was in the range of 81–85% and consisted of organic (60–65%) and aqueous (20%) phases. The oxygen content in the organic phase decreased to 0.5%. The organic phase consisted of aliphatics (62%) such as cyclohexane, methylcyclohexane, dodecane, and aromatics (38%) such as toluene, ethylbenzene, propylbenzene, etc.

It is clear from the above that lignin must be mixed with lignin oil or preheated for continuous lignin hydrotreatment. The depolymerization of lignin in continuous reactors has some advantages over batch reactors, such as a shorter contact time of the lignin fragments, which can lead to the formation of the desired products such as phenolics or aromatics. In batch reactors, on the other hand, the contact time is relatively longer than in continuous reactors, which can lead to the formation of saturated cyclic alkanes as the main product. The selectivity of the product also depends on the catalyst. Therefore, continuous reactors are suitable when working with active catalysts such as noble metals. However, when conventional catalysts, such as Ni/Co promoted Ni/W based catalysts, are used in continuous reactors, a moderate contact time might be required for efficient removal of oxygen in the formation of aromatics. This could be achieved by varying the space velocities to obtain the desired product. One of the problems of the fixed bed reactor is the pressure drop due to char formation during lignin depolymerization. The semicontinuous reactor has some advantages over the fixed bed reactor, such as homogeneous mixing of catalyst and reactants, removal of the desired product at regular intervals, etc. Meier et al. used milled wood lignin and organocell lignin for the hydropyrolysis in a semicontinuous reactor.^155^ The schematic diagram of the semicontinuous reactor is shown in Figure 14(D). The reactions were carried out at 400 °C, 140 bar H_2_ and 6 h with Ni, Cr_2_O_3_ on Al_2_O_3_–SiO_2_. After the hydropyrolysis reaction, three product fractions were obtained: (i) light oil from the condenser, (ii) middle oil in the reactor, (iii) acetone insoluble fraction (char). From the optimization of the reaction parameters such as hydrogen pressure and residence time, it was concluded that the reaction pressure has a significant influence on the total oil yield. The highest yield of lignin oil (>80%) was obtained for organocell lignin, and the quantified products are mainly monophenolics (12.3%). In another report, organocell lignin was mixed with different slurry oils for hydrocracking in a semicontinuous reactor using NiMo and zeolite catalysts.^156^ The reaction temperature was in the range of 375–450 °C and the hydrogen pressure was 75–180 bar. The authors reported that char formation was lowest (0.3%) when slurry oils were used. In the first case, the char yield was higher.^155^ The highest oil yield (81.9%) was obtained when lignin was mixed with lignin-derived slurry oil (at 375 °C, 100 bar H_2_, and 2 h). The quantified monomer yield was 12.8%, including phenol, guaiacol, xylenols and cresol etc. In the case of the zeolite catalyst, the formation of char (29.4%) was high, as zeolites are known for their high acidity properties leading to a repolymerization reaction. The oil obtained in this case was 54.1% and the quantified monomer yield was only 7% (see Table 5).

**Table 5:** Lignin Hydrotreatment in Continuous/Semicontinuous Reactors

S. no.	lignin source	catalyst	reaction conditions	oil yield (%)	major product	ref.
1.	different lignin sources (lignosulfonates, thio-lignin, bark lignin, lignin from hydrolysis of wood)	FeS (cocatalysts: Cu, Ag, Sn, Co, Ni, Zn, Mo)	lignin is mixed with lignin tar/naphthene hydrocarbon (tetralin)/phenols/gas oil/creosote oil/hydrogenated oil from coal)	95	alkylphenolics 50%	(152)
2.	Kraft lignin	combination of Fe, Mo, Co, Ni on supports Al_2_O_3_ and SiO_2_	340–460 °C, 34–172 bar H_2_, and WHSV 1–10 pound of lignin h^–1^ pound catalyst^–1^, solvent: process–derived oil	62.5	alkylphenolics 37.5%	(153)
3.	pyrolytic lignin from hog fuel	CoMo/Al_2_O_3_	400–415 °C, 140 bar hydrogen, WHSV 0.51–1 g_lignin_ g_cat_^–1^ h^–1^	60–65	aliphatics hydrocarbons	(154)
4.	organocell lignin	NiO, Cr_2_O_3_ supported on Al_2_O_3_ and SiO_2_	400 °C, 140 bar H_2_ and 6 h	>80	monophenols 12.3%	(155)
5.	organocell lignin	sulfided NiMo	lignin in slurry oil, 450 °C, 180 bar H_2_, 2 h	81.9	total monomers 12.8%	(156)
6.	organocell lignin	zeolite	lignin in slurry oil, 375 °C, 100 bar H_2_, 2 h	54.1	total monomers 7%	(156)
7.	pyrolytic lignin from rice husk	Ni/HZSM–5	500 °C, 40 bar H_2_, 3 h	49.8	total monomers 35.6%	(157)
8.	pyrolytic lignin from rice husk	Ni/mUSY	500 °C, 40 bar H_2_, 3 h	∼48	total monomers 32.2%	(157)
9.	pyrolytic lignin from rice husk	Ni/Al–MCM–41	500 °C, 40 bar H_2_, 3 h	∼46.5	total monomers 29.8%	(157)
10.	pyrolytic lignin from rice husk	Ni/Al_2_O_3_	500 °C, 40 bar H_2_, 3 h	∼47	total monomers 27.2%	(157)
11.	pyrolytic lignin from rice husk	Ni/SiO_2_	500 °C, 40 bar H_2_, 3 h	∼40.5	total monomers 16.1%	(157)
12.	pyrolytic lignin from rice husk	Ni/HZSM–5	500 °C, 1 atm N_2_, 3 h	45.8	total monomers 22.9%	(157)
13.	pyrolytic lignin from rice husk	Ni/mUSY	500 °C, 1 atm N_2_, 3 h	48.6	total monomers 20.0%	(157)
14.	pyrolytic lignin from rice husk	Ni/Al–MCM–41	500 °C, 1 atm N_2_, 3 h	38.5	total monomers 22.0%	(157)
15.	pyrolytic lignin from rice husk	Ni/Al_2_O_3_	500 °C, 1 atm N_2_, 3 h	40	total monomers 23.2%	(157)
16.	pyrolytic lignin from rice husk	Ni/SiO_2_	500 °C, 1 atm N_2_, 3 h	39	total monomers 12.8%	(157)
17.	pyrolysis oil from pine wood	Ni–Cu/SiO_2_–ZrO_2_	410 °C, 200 bar H_2_,0.2 g_P_ g_cat_^–1^ h^–1^	42	-	(158)

Interestingly, Cai et al. used a two-stage reactor system for the hydrocracking and cracking of pyrolytic lignin.^157^Figure 14(E) shows the reactor setup, where lignin is vaporized in the first stage at 600 °C and passed through the second stage of the reactor, where the catalytic bed has a temperature of 500 °C. Hydrogen and nitrogen were used for the hydrocracking and cracking of pyrolytic lignin, respectively. For this study, the author synthesized Ni on various acidic supports such as HZSM–5, mUSY, Al–MCM–41, Al_2_O_3_ and SiO_2_, etc. Regardless of the catalyst type, the hydrocracking experiments resulted in higher yields of oil and monomers compared to the cracking experiments (see Table 5). This is due to the promotion of Ni and acidic sites during hydrocracking (hydrodeoxygenation and depolymerization reactions) in the presence of hydrogen. In addition, the char content during hydrocracking is relatively lower than during cracking. The oil and monomer yields achieved in hydrocracking with a Ni/HZSM–5 catalyst was 49.5% and 35.6%, respectively. The differences in the catalytic activity of the Ni-supported catalysts are due to the available Ni and acid sites and depend on the interactions between the metal and support. In the case of the cracking process, a high lignin oil content was obtained with Ni/mUSY (45.8%) and Ni/HZSM–5 (48.6%). However, no major difference was observed in the monomer yields (22–23.2%) for all Ni–based catalysts, except in the case of the Ni/SiO_2_ catalyst (12.8%). The authors also demonstrated the HDO mechanism using phenol as a model compound. It was reported that the acidic sites are responsible for the tautomerization of phenol, followed by hydrogenation at the active Ni sites forming cyclohexanedienol, and finally dehydration at the acidic site leading to the aromatics formation. Compared to mesoporous supports, the microporous support HZSM–5 is more favorable for monoaromatics and suppresses the formation of char.

Yin et al. studied the hydrotreatment of fast pyrolysis oil in a continuous reactor system with Ni–Cu/SiO_2_–ZrO_2_ catalyst.^158^ The reactor setup consists of three reactors, as shown in Figure 14(F). Each reactor was maintained at a different temperature: first reactor: 75 °C, second reactor: 180 °C, and third reactor: 180–410 °C. The reactor pressure was maintained at 200 bar hydrogen. It is reported that the yield of product oil decreased from 86–42% when the temperature of the third reactor was increased from 180–410 °C. However, the monomers detectable by GC were low in the lignin oils obtained at low temperatures (180 °C) and higher in the lignin oils obtained at 410 °C. This is due to greater depolymerization of the pyrolysis oil, which leads to the formation of low molecular weight oils and contributes to an increase in the yield of monomers such as aldehydes, ketones, acids, phenolics, aromatics and hydrocarbons, etc. The yield of phenolics, aromatics, and hydrocarbons increases with temperature at the expense of aldehydes and sugars. The oxygen content of the lignin oil obtained at 410 °C was reduced to <13%. Table 5 shows a summary of the different continuous and semicontinuous catalytic hydrotreatment approaches for lignin depolymerization.

J. Horacek et al. studied the conversion of lignin into valuable chemicals in a semi–continuous tubular reactor using a conventional NiMo/Al_2_O_3_ catalyst at 320–380 °C and 40–70 bar hydrogen pressure.^160^ The authors optimized various reaction parameters such as temperature, hydrogen pressure, catalyst to lignin ratio, and catalyst activation method etc. It is reported that the aqueous phase consists mainly of alkylphenolics and the organic phase contains aromatics and naphthenes. The main products in the gas phase were carbon dioxide, methane, and H_2_S in the case of lignosulfonates. In the reaction carried out below 380 °C, the formation of char was higher and decreased with increasing temperature. The high catalyst-to-lignin ratio has a positive effect on the formation of liquid products due to the long contact time between the active sites and the lignin fragments. In addition, a high partial pressure promotes the depolymerization of lignin by stabilizing the primary lignin fragments and thus increasing the yield of liquid products. The sulfidation method (*in situ* or presulfidation) has the least effect on product yield and is slightly higher in the case of the presulfided catalyst.

As discussed above, there are few reports on the continuous catalytic hydrotreatment of lignin. However, there are reports available on the hydrotreating of pyrolysis oil in continuous fixed bed reactors. Since pyrolysis oil is rich in lignin content, the lignin obtained from it is referred to as pyrolytic lignin.^161^ The hydrotreatment of pyrolytic lignin has already been explained in the previous sections. The upgrading of pyrolysis oil is one of the most important intermediate steps before it is used in the petroleum refineries for coprocessing in FCC plants with the conversion material (e.g., vacuum residue (VR)). This is because the direct use of pyrolysis oil leads to the formation of char and a low yield of the desired products.^162^ For the upgradation of pyrolysis oil, Biomass Technology Groups (BTG), Netherlands, in collaboration with the University of Twente, has developed a reactor in which HPTT technology was used. In this process, the pyrolysis oil is treated at 300–340 °C and 140 bar with a residence time of several minutes.^142^ As no catalyst or hydrogen is used in this technique, high pressures are generally used to avoid the formation of water vapor, which could accelerate the formation of char. The advantages of HPTT are that it is a cost-effective deoxygenation process, the saturation of the aromatic rings is lower and the quality of the pyrolysis oil is improved by the removal of oxygen and water. Mercader et al. performed the HPTT experiments in a continuous tubular reactor in the temperature range of 200–350 °C, 200 bar H_2_, and different residence times (3.3–3.5 min).^142^ The authors found that the pyrolysis oil concentration has a direct effect on the molecular weight of the hydrotreated pyrolysis oil. When the pyrolysis oil is only subjected to HPTT, an increase in the molecular weight of the oil phase was observed, and this effect was reversed when it was diluted with water. The increase in the molecular weight of the HPTT oil was due to the repolymerization of the sugar fraction contained in the pyrolysis oil. Temperature and residence time have no significant influence on the properties of the product oil. However, the dilution of the pyrolysis oil has different effects on the desired decarboxylation and the undesirable increase in molecular weight. A significant decrease in water and oxygen content was observed in the HPTT oil. However, the molecular weight of the HPTT oil also has a great influence on the corefining process, i.e. on the miscibility with the refinery stream. Therefore, further refining of the HPTT oil using the HDO technique is required to reduce the molecular weight and lower the oxygen content. The advantage of the HPTT process over the HDO process is that hydrogen consumption is lower compared to the direct HDO process for pyrolysis oil, as the HPTT oil has a very low oxygen content and water-soluble components are excluded during the HPTT process. In addition, the absence of low molecular weight components in the HPTT oil leads to a high hydrogen partial pressure in the reactor, which in turn shortens the reaction times. For the depolymerization of lignin using the hydrotreatment technique, high temperatures (>350 °C) and high pressure are required to produce monomers. Under such harsh reaction conditions, the aromatic rings can become saturated. This increases production costs, as this process consumes a lot of hydrogen and produces low valuable chemicals such as cyclohexane. HPTT technology therefore could be used for the lignin pyrolysis oil. The reaction can be carried out at low temperatures to obtain aromatics instead of alkanes.

As discussed in the previous sections, solvent-free hydrotreatment of lignin can be carried out in batch, semicontinuous and continuous fixed-bed reactors. However, there are advantages and disadvantages associated with each mode of operation, which are listed in Table 6

**Table 6:** Comparison of Batch, Semicontinuous, and Continuous Fixed Bed Reactors for the Hydrotreatment of Lignin under Solvent-Free Conditions

parameter	batch	semicontinuous	continuous fixed bed
1. solid lignin feed	suitable	not suitable: in order to process, lignin must be mixed with lignin oil or alkylphenolics	not suitable: lignin must be mixed with lignin oil or alkylphenolics
2. catalyst particle size	from micro size to pelletized catalyst particle can be used	a catalyst basket is suitable with pelletized particles	a fixed catalyst bed required pelletized catalyst particles
3. homogeneous mixing	possible	possible	not applicable
4. pressure drop	not applicable	not applicable	yes, due to the formation of char
5. separation of aqueous, organic and gas fractions	can be done only after the reaction	can be done in semicontinuous mode	separation of aqueous and organic phased can be done continuously
6. lignin conversion	high	low to moderate	low
7. yield of aromatics	due to longer contact time the aromatics yield may decreases.	moderate contact time can improve aromatics yield	aromatic yield could be improved with manipulation of contact time/whsv
8. large scale production	limited scope	suitable	suitable

### 5.3 Comparison of Sulfur-Free, Sulfur-Containing, and Pyrolytic Lignins Hydrotreatment

The solvent-free catalytic hydrotreatment of lignin is mainly concerned with the cleavage of C–O, C–C and C–S (in the case of sulfur containing lignins). The reaction network is the same for all types of lignin (*vide infra*, Section 6.1). During the reaction, lignin produces oligomers and forms monomers in the subsequent steps.^26,27,31,146^ However, the conversion of lignin to monomers depends mainly on the type of lignin, the catalyst and the reaction conditions, etc. The *M*_w_ of lignin has a great influence on the hydrotreatment, as the *M*_w_ of pyrolytic lignins is far less than that of parent lignin^163,164^ and therefore has higher activity than simple lignins (sulfur-free and sulfur-containing). Since pyrolytic lignins are obtained from pyrolysis oil and are sulfur-free. Therefore, the catalytic activity in the conversion of sulfur-containing and sulfur-free lignins is compared and discussed as follows:

Various mono- and bimetallic catalyst systems such as metal sulfides (Ni, Mo, Fe),^26,28,113,125,126,151^ phosphides (Ni, Mo, W),^30,32,133^ noble metals (Ru, Pd, Pt),^27,31,118,119^ zeolites (ZSM–5, USY)^29,157^ and other transition metal catalysts (Cu, Ga, Zn)^27,29^ have been described for hydrotreatment of different types of lignins. Regardless of the type of lignin, noble metal catalysts are the most active catalysts for the production of aromatics.^27,31,34,118,146,151^ However, noble metal catalysts are not suitable for sulfur-containing lignins, as the active metals convert to sulfides (deactivation).^31^ Therefore, noble metals are more suitable for the hydrotreatment of sulfur-free and pyrolytic lignins. Other catalytic systems such as metal sulfides and metal phosphides are also used for the conversion of lignins. When metalsulfides are used for sulfur-free lignins, a sulfur source (e.g., DMDS) is an additional requirement for maintaining the active phase of the catalyst. The reason for this that the sulfur in lignins has an additional advantage over metal sulfides and phosphide catalysts. In the case of metal sulfides, the sulfur contained in the lignin keeps the catalyst in the active phase, i.e., the catalyst is stable during the hydrotreatment process. Metal phosphides, on the other hand, are unstable as these catalysts tend to form metal sulfides (i.e., another active form) during the hydrotreatment process. Therefore, metal phosphides could still be used for the hydrotreatment of sulfur-containing lignins, as both phases (metal sulfides and phosphides) are active in the hydrotreatment reaction. Compared to the metal sulfides, the metal phosphide catalysts are relatively active in the conversion of sulfur-containing lignins (e.g., Kraft). The transition metal catalysts and supported zeolites have a moderate monomer yield. The main factors influencing the yield of monomers and aromatics from lignins are discussed in detail in section 6.

## 6 Key Factors in Solvent-Free Hydrotreatment of Lignin

### 6.1 Reaction Conditions, Catalyst Acidity, and Reaction Network

**Scheme 1:**
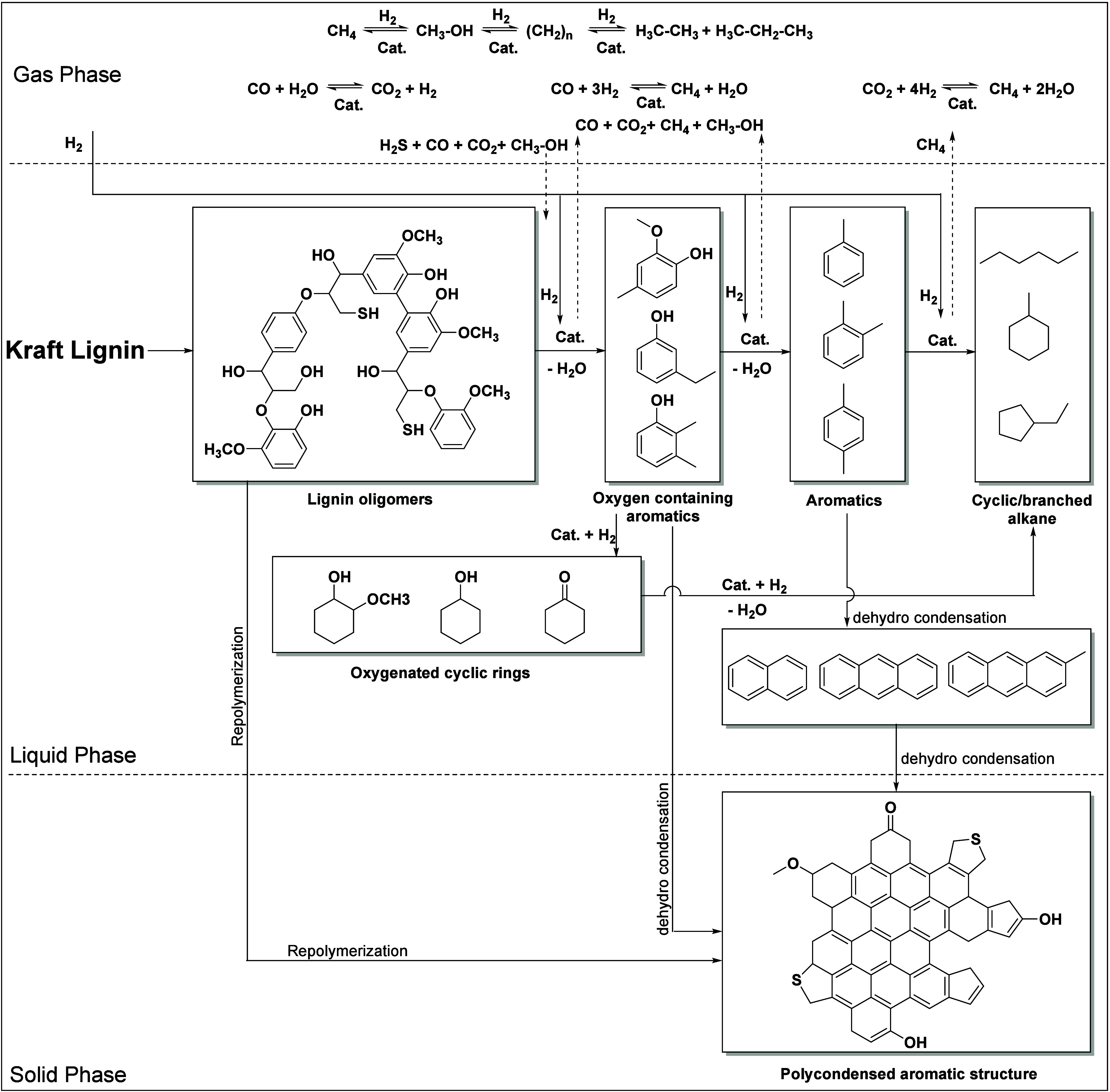
Reaction Network in Gas, Liquid, and Solid Phased during the Hydrotreatment of the Kraft Lignin under Solvent-Free Conditions Reproduced from ref (133). Copyright 2019 ACS Publications.

The reaction parameters are important factors that influence the extent of depolymerization and the formation of monomers. It is known from the literature that the solvent-free depolymerization of lignin is proportional to the reaction temperature.^32,119,125,133^ However, if valuable products such as oxygen-free aromatics like BTX are to be formed, the reaction temperature must be optimized, as different reactions occur in the gas, liquid and solid phases, as shown in Scheme 1. Scheme1 shows the hydrotreatment of Kraft lignin under solvent-free conditions, and this reaction network is almost similar in the case of sulfur-free lignins using noble and non-noble metal catalysts.^27,30,31,34,62,80,118,119,146,178^ The only exception is the formation of H_2_S in the gas phase with sulfur-containing lignins. During catalytic hydrotreatment, the lignin melts (at 140–170 °C) and serves as a solvent, while large polymeric species are fragmented into oligomers and monomers. As shown in Scheme 1, several consecutive, parallel reactions take place during the formation of monomers. In general, harsh reaction conditions are used for hydrotreatment than for hydrodeoxygenation. As high temperatures and pressures are used in the hydrotreatment technique, several reactions such as hydrogenolysis (breaking of C–C, C–X (X = heteroatoms like S, O bonds), saturation of double bonds) take place simultaneously. Saturation of the aromatic ring cannot be avoided under the harsh conditions of the hydrotreatment technique. For example, Jiang et al. investigated the hydrogenolysis of lignin model compounds such as DPE and BPE with electron-rich Ni nanoparticles on α-Al_2_O_3_.^165^ Under the mild reaction conditions (180 °C and 5 bar), the hydrogenation of the aromatic ring led to the formation of ring-hydrogenated products such as cyclohexanol and cyclohexene. Similar observations were made in the case of Ru/C (for hydrogenolysis)^166^ and Ru on Ga-doped HZSM-5 (HDO of the lignin model compounds).^167^ During the hydrotreatment, lignin forms oligomers and other lignin fragments. As discussed previously, lignin contains a large number of oxygen-containing functional groups, and the cleavage of β–O–4 bonds (C–O–C) is relatively higher than that of other bonds (C–C). This leads to the formation of more lignin fragments such as oligomers with hydroxyl groups. Therefore, the process of hydrotreating facilitates the HDO of lignin fragments in the subsequent stage, resulting in the formation of oxygen-free/low-oxygen aromatics (alkylphenolics). Alkylphenolics can be subjected to ring hydrogenation and/or HDO. Ring hydrogenation of alkylphenolics produces oxygenated rings such as cyclohexanol and its derivatives. On the other hand, HDO of alkylphenolics leads to the formation of oxygen-free aromatics (BTX). As the residence time in batch reactions is relatively long compared to fixed bed reactors, the hydrogenation of oxygen-free aromatics leads to the formation of cyclic/branched alkanes. Polycyclic aromatics and polycondensed aromatics are formed by the dehydrocondensation reaction of aromatics and/or the repolymerization of oligomers. The cause of polycondensed aromatics is the acidity of the catalyst and the high reaction temperatures. In the case of the gas phase, the main products observed are C_1_–C_3_ hydrocarbons, CO, CO_2_, and H_2_S (in the case of Kraft lignin).^26,125,151^ It should be noted that the formation of gas and char is also proportional to the reaction temperature. It is therefore very important to know the dependence of the reaction network on the reaction temperature so that the desired products are formed during the depolymerization of lignin. Since there is no solvent in the reactor, sufficient hydrogen pressure is required to suppress the repolymerization of lignin fragments and reduce the formation of char (solid phase). Instead of hydrogen, other gases such as methane can be used for the depolymerization of lignin, which generates hydrogen through a reforming process under *in situ* conditions.^29^ In reactions with a temperature of more than 400 °C, the of char and gas phase content usually increases at the expense of gasification of the monomers present in the reaction mixture.^119,125,126^ In addition, the choice of catalyst also plays an important role in lignin depolymerization and monomer yield. Catalyst properties include characteristics such as the ability to cleave the lignin polymer (acid/base) and the removal of oxygen (metal). For example, the depolymerization of Kraft lignin over NiMo on basic/neutral supports (MgO–La_2_O_3_ and AC) was found to have a high monomer yield and low char compared to acidic supports (Al_2_O_3_, ZSM5).^26^ In the case of metal phosphide (NiMoP) supported on AC, TiO_2_, SiO_2_–Al_2_O_3_, SiO_2_, MgO–La_2_O_3_, the best results were obtained for SiO_2_.^30^ These results shows a different support is more suitable for different active components, which also depends on the reaction conditions. The acidity of the catalyst must be optimized in order to control the repolymerization reaction. Regardless of the catalyst type, the optimum reaction temperature for most batch reactions is 400 °C, which leads to a high yield of monomers and aromatics.

### 6.2 Analytical Techniques

#### 6.2.1 Gel Permeation Chromatography

The extracted lignins contain various chain lengths with different masses, which is why it is necessary to determine the lignin property in the form of the average molecular weight. There are two very common average molecular weights for polymers, i.e., *M*_w_, and *M*_n_. The PDI is the ratio of *M*_w_, and *M*_n_. The corresponding equations are given below:^168−170^
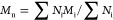
3
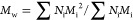
4
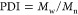
5

**Figure 15:**
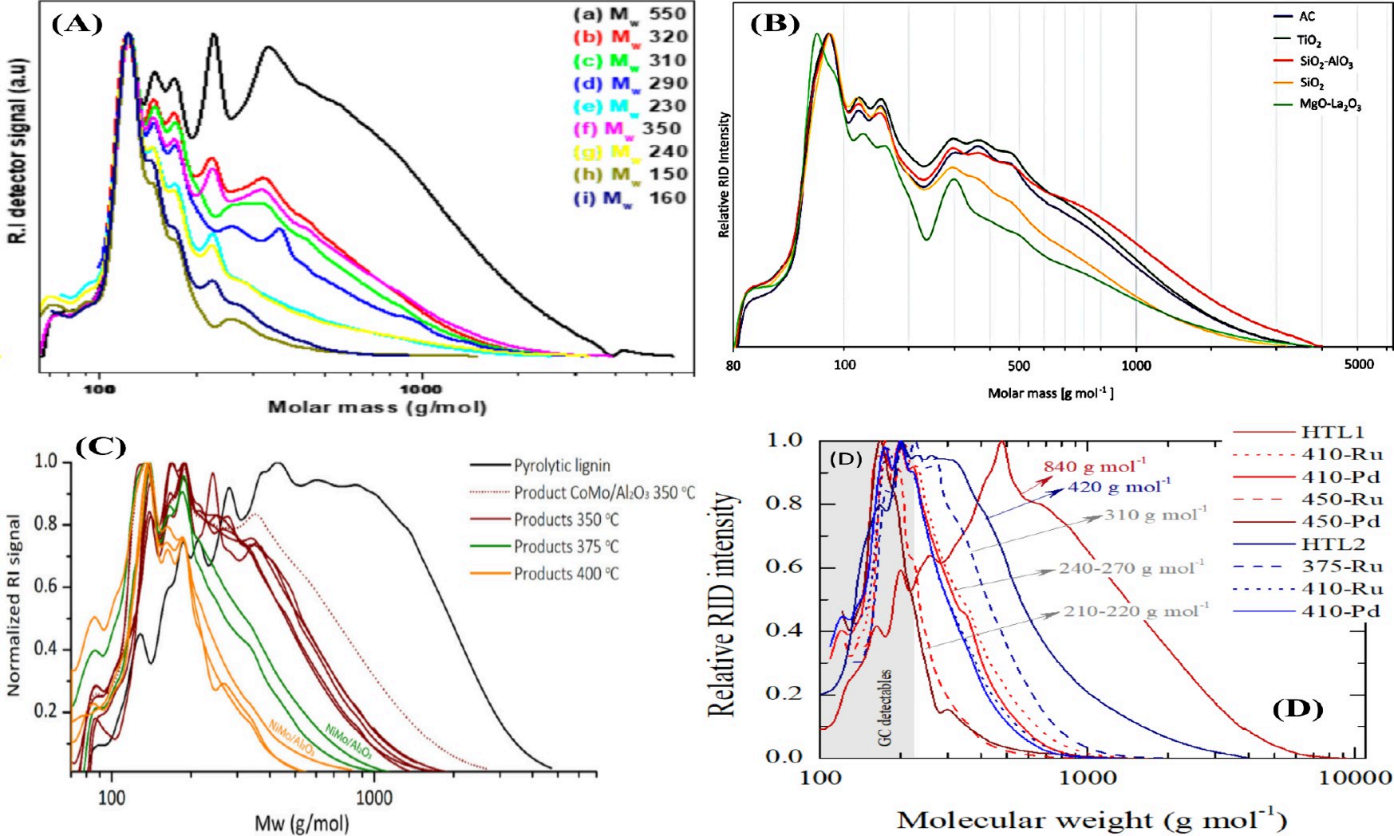
Gel permeation chromatograms of (A) hydrotreated Kraft lignin at different temperatures over 20NiMoP/AC,^133^ (B) hydrotreated Kraft lignin over NiMoP on various supports (reproduced with permission from ref (30); copyright 2021 RSC Publications), (C) hydrotreated pyrolytic lignin from pine wood over NiMo and CoMo supported on Al_2_O_3_ catalysts (reproduced with permission from ref (34); copyright 2019 Elsevier), (D) hydrotreated HTL oils from BPS over Ru, PD catalysts at different temperatures (reproduced with permission from ref (119); copyright 2021 Elsevier).

Gel permeation chromatography is the basic technique for estimating the average molecular weight of lignin and the extent of lignin depolymerization. The average molecular weight of extracted lignin depends on the wood type and extraction methods. The average molecular weight of Kraft, soda, organosolv, and pyrolytic lignins is in the range of 1500–25000, 1000–15000, 500–5000, and 350–1900 g mol^–1^, respectively.^163,164^Figure 15 shows the GPC analysis of various lignin sources and their hydrotreated products. A broad peak is generally observed in the chromatogram, indicating the presence of lignin polymer fragments/oligomers of different sizes. The reaction temperature and time have a great influence on the reduction of the average molecular weight of the hydrotreated lignin. In the case of Kraft lignin hydrotreated at 400 °C with a 20NiMoP/AC catalyst, GPC analysis showed a broad peak (Figure 15(A)). With increasing reaction time and temperature, the peak becomes narrow and sharp when the reactions are carried out at >450 °C.^133^ The catalyst properties also influence the average molecular weight of the lignin oils. However, the effect is relatively lower than the reaction temperature. The lignin oils (obtained at 400 °C for 4 h) from Kraft using NiMoP dispersed on different supports such as AC, TiO_2_, SiO_2_–Al_2_O_3_, SiO_2_ and MgO–La_2_O_3_ etc. show a similar type of chromatograms with an average molecular weight in the range of 360–450 g mol^–1^ (Figure 15(B)).^30^ However, a keen observation of the chromatogram reveals that the peak extends to higher number for SiO_2_–Al_2_O_3_ supports, while narrow peaks are observed for SiO_2_ and MgO–La_2_O_3_ supports. However, since NiMoP shows a similar chromatogram on SiO_2_ and MgO–La_2_O_3_ supports, the final monomer yield depends on the total number of monomers detected by GC analysis and is high in the case of the NiMoP/MgO–La_2_O_3_ catalyst. These results show that the catalyst support also has a significant influence on the reduction of the average molecular weight. Compared to the technical lignins, the pyrolytic lignin shows a narrow peak in the chromatogram (see Figure 15(C)). This is due to the fact that pyrolytic lignins are derived from pyrolysis oil (i.e., from woody biomass that has already been treated at very high temperatures). Therefore, the pyrolytic lignin treated with hydrogen showed very sharp peaks in the reaction at 400 °C than at 350 °C (Figure 15(C)).^34^ The molecular weights of the lignin oils obtained from pyrolytic lignins are lower than those of the corresponding parent lignins. A similar trend can be observed in the hydrothermal liquefaction (HTL) of stillage. The HTL oil obtained at 305 °C (HTL1) and 350 °C (HTL2) had an average molecular weight of 840 and 430 g mol^–1^, respectively.^119^ Hydrotreatment of the HTL oil with Ru and Pd based catalysts resulted in narrow peaks in the GPC analysis, and the average molecular weight of the hydrotreated HTL oil is slightly lower for HTL2. However, the products detectable by GC are within a limited range, as shown in Figure 15(D). From the GPC analysis, it can be generalized that for a given set of hydrotreatment conditions, a lower *M*_w_ of the feedstock leads to a lower *M*_w_ of the resulting oil and a higher monomer yield. In other words, if the average *M*_w_ of the various feedstocks are in the order of lignin > pyrolytic lignin > HTL oil, then the resulting hydrotreated product follows the same trend.

#### 6.2.2 Qualitative and Quantitative Analysis (GC–MS, 2D–GC, NMR)

The analysis of lignin oil is another important aspect of lignin depolymerization. The general and widely used technique for the qualitative and quantitative analysis of lignin oils is GC–MS. Since lignin is a complex mixture, it consists of several types of chemicals such as oxygen-free aromatics, oxygenated aromatics, liner/cyclic aliphatics, oxygenated cyclohexanes and derivatives of all these classes, etc. The separation and identification of each component in the complex lignin oil by GC–MS analysis is tricky since there are several components with the same carbon and oxygen number with a general formula. Therefore, differentiation of the individual components with a general GC is not easy and there may be deviations in the quantified product yields. Advanced analytical techniques such as GC×GC (2D–GC) analysis is also used to efficiently separate and identify the components in lignin oil. The main advantage of this technique is that various classes of compounds can be separated in a clearly defined area. A typical GC×GC chromatogram is shown in Figure 9(B). In the chromatogram, a clear separation of the various group components such as cyclic alkanes, linear/branched alkanes, oxygen-free aromatics, polycyclic aromatics, ketones/alcohols, acids, guaiacols, alkylphenolics, catechols etc. can be recognized. In general, GC-detectable (monomeric) yields are proportional to the low average molecular weight of the lignin oil, i.e. the lower the molecular weight of the oil, the higher the GC-detectable monomeric products. However, even with low average molecular weights of lignin oil, there is a limitation to the detection of products in GC. Most reports have found that for reactions performed up to 400 °C, the monomer yield is always less than the lignin oil yield due to the presence of lignin oligomers, which also show up in GPC analysis as the peak extends to higher molecular weight region. For example, Hita et al. presented the GC detectable product range in the gel permeation chromatogram of hydrotreated lignin oils, which is below 230 g mol^–1^ of molecular weight (see Figure 15(D)).^119^ A large proportion of the peaks that are not detectable by GC indicate the presence of oligomers or high molecular weight monomers. To understand the linkages still present after hydrotreatment, the sample need to be analyzed, which can be done using NMR techniques such as ^13^C NMR and ^1^H–^13^C HSQC NMR. The overall bonds present in the lignin oil can be understood by comparison with the ^13^C NMR spectra of the starting material. For example, the ^13^C NMR spectra of Alcell lignin and its various fractions show peaks related with various functional groups such as aliphatic (δ 0–38 ppm), methoxy (δ 52–58), α-, β-, γ- ether bonds (δ 58–100), unbranched aromatics (δ 100–125), branched aromatics (δ 125–160), carbonyl groups (δ 160–210), etc. (see Figure 6(E,F)). After hydrotreatment of Alcell lignin and its fraction, ^13^C NMR showed drastic changes, particularly in the aliphatic and aromatic compound regions, where the intensity of peaks increased; the peaks related to ether and carbonyl groups diminished. Using ^13^C NMR, a whole class of bonds could be identified and quantified. However, the identification and quantification of a particular linkage can be done using ^1^H–^13^C HSQC NMR.^28,118,126^ The linkages of particular bonds in Alcell lignin and its fractions have already been discussed in the previous section (Figure 6(A–D), *vide supra*). The representative HSQC NMR spectra of hydrotreated soda lignin and pyrolytic lignins are shown in Figure 10(B) and Figure 13(C), respectively.

#### 6.2.3 Oxygen and Hydrogen Content: Van–Krevelen Plot

**Figure 16:**
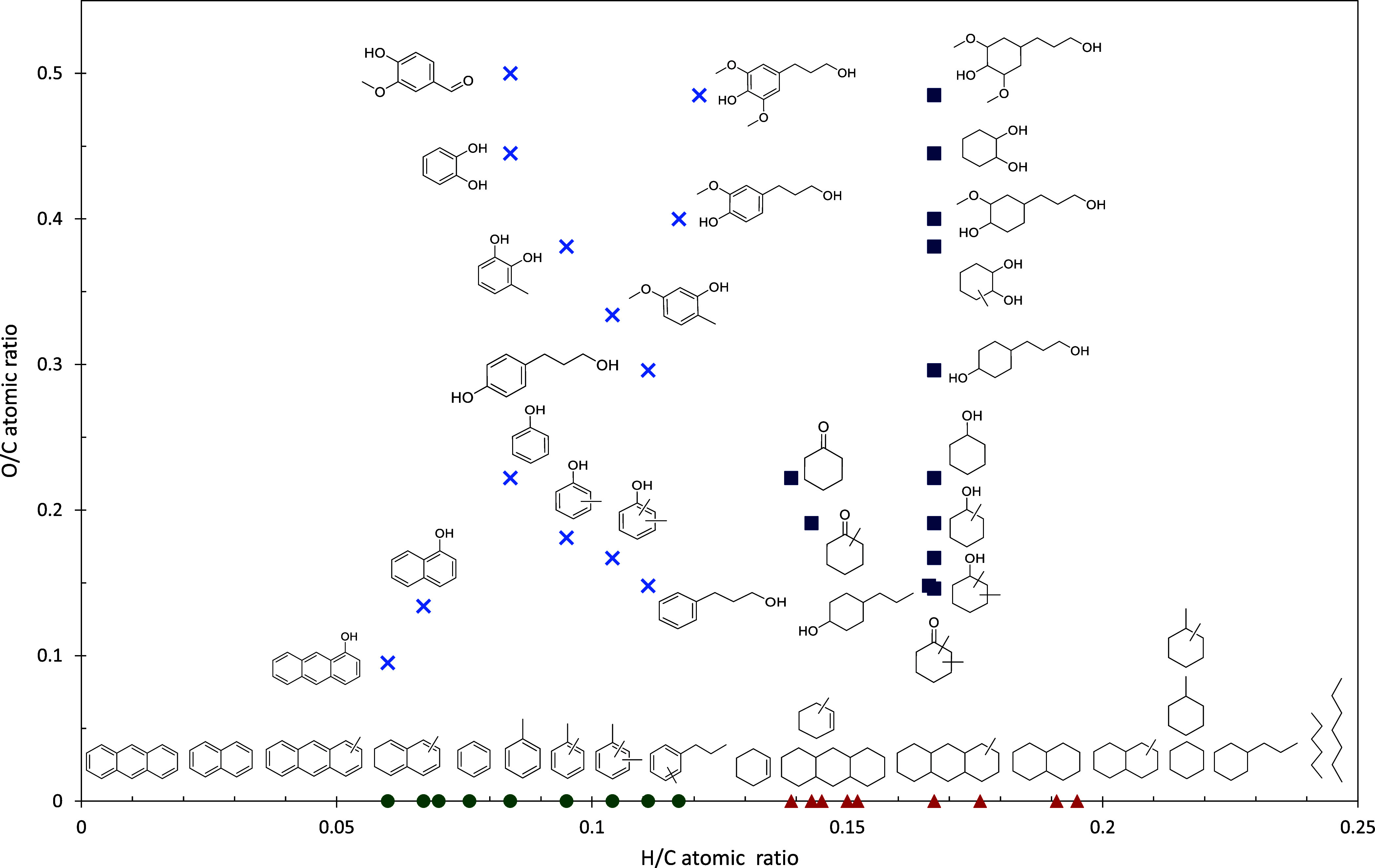
Van–Krevelen plot for various products of lignin monomers (Blue cross mark: Oxygenated aromatics, Green dot: oxygen-free aromatics, Royal blue square: oxygenated cyclohexanes, Red triangle: aliphatics).

The Van–Krevelen diagram is a graphical representation of the O/C atomic ratio versus the H/C atomic ratio and is useful for estimating compound categories, especially for the biomass samples. Figure 16 shows a typical Van–Krevelen diagram for monomers derived from the hydrotreatment of lignin. As this report focuses on the solvent-free hydrotreatment method for the production of aromatics from lignin, the products observed in this process are of importance. The Van–Krevelen diagram is one of the analytical techniques used to estimate product type based on oxygen and hydrogen content. Figure 16 shows the Van–Krevelen diagram for the various lignin fragments and monomers that are expected to form after the lignin hydrotreatment reaction. For better understanding, only one of the isomers of each component is considered in the diagram i.e., cumene and propyl benzene, for example have similar H/C ratios. Here, the compound categories are divided into four classes, namely oxygen-free aromatics, oxygenated aromatics, oxygenated cyclohexane, aliphatics, etc. The literature shows that in the hydrotreatment of lignin under solvent-free conditions, the most important product category is alkylphenolics.^26−32,118,119,125,126,133,146^ The main repeating units in the lignin polymer, *p*-coumaryl alcohol, coniferyl alcohol, and sinapyl alcohol etc. consist of oxygen, so that these primary units are found in Van–Krevelen diagram between the O/C ratio 0.5–0.29 and the H/C ratio 0.1–0.14. The oxygenated aromatics (alkylphenolics and catechols) are generally observed at an O/C ratio of 0.14–0.22 and an H/C ratio of 0.08–0.11. For high-value chemicals such as BTX and other oxygen-free aromatics derived from lignin, the H/C ratio ranges from 0.05 to 0.12 (the O/C ratio is zero as these compounds do not contain oxygen). These products are derived from the deoxygenation of oxygenated aromatics as shown in Scheme 1 (such as phenolics, guaiacols, cresols, and corresponding derivatives, etc.). The hydrogenation of aromatics leads to the formation of aliphatics (cyclic and linear) and this class of compounds contains a high hydrogen content, which is why it appears in the Van–Krevelen diagram with a high H/C ratio of 0.13 to 0.2. These products are generally observed when the hydrotreatment reaction is carried out under harsh reaction conditions (high reaction temperatures and high pressure). On the other hand, oxygenated cyclohexane appears at an H/C ratio of 0.13–0.16 and an O/C ratio of 0.15–0.5. In the Van–Krevelen plot, extracted lignin appears at O/C: 0.36 and an H/C 1.15 and Alcell lignin at an O/C value of 0.34–1.05. In the depolymerization of lignin, the main expected product class is oxygen-free aromatics, therefore the expected H/C ratio must be below 0.12. Since the first and second main products of hydrotreated lignin/pyrolytic lignins are alkylphenolics and oxygen-free aromatics, respectively. Hence in the Van–Krevelen plot hydrotreated lignin/pyrolytic lignin (oils) are observed between the O/C ratio: 0.15–0.02 and the H/C ratio: 0.8–1.6. The Van–Krevelen diagrams for hydrotreated Alcell and Kraft lignins are shown in Figures 5(D) and 12(B), respectively.

## 7 Techno-economical Evaluation on Solvent-Free Catalytic Hydrotreatment of Lignin

The potential for scale-up and the techno-economic application of lignin upgrading depends on the lignin source, availability, extraction technology and technical requirements. In catalytic hydrotreating, the production of oxygen-free aromatics instead of oxygenated aromatics or saturated hydrocarbons (of low value) is crucial for the profitability of the investment. The cost of the feedstock is very important for the construction of the hydrotreating plant. This is because pulp, paper, and biorefineries produce enormous amounts of lignin, which is considered low value and is used for power generation.^10^ Therefore, the conversion of technical lignin into aromatic compounds offers significant economic benefits and a reasonable return on investment. For the production of aromatics, it is advantageous for the industry to use the established Kraft process. The hydrotreatment process has been presented as an economic necessity for the production of valuable chemicals in addition to biofuels from lignin.^171−175^ However, detailed information on the technical and economic performance is required for the commercialization of lignin to aromatic chemicals. However, scaling up lignin depolymerization technology to an industrial level remains a major challenge, as this evaluation is still at an early stage and only limited knowledge is available. Despite the inherent uncertainties, an early stage technology assessment can be extremely valuable. This is because it provides insight into the capabilities of cutting-edge technology and provides incentives and targets for further advances in lignin depolymerization. The evaluation of the depolymerization of lignin into valuable chemicals includes technical analysis, process modeling and economic feasibility. Process modeling can be performed using software such as Aspen Plus.^176^ Reports on the techno-economic evaluation of solvent-free lignin depolymerization are scarce, as there are few research reports on this topic. One report on the techno-economic evaluation of direct HDO of lignin by I. Vural Gursel et al. was found. In this report, the authors compared different approaches for the production of aromatics, such as direct HDO of lignin, pyrolysis, and hydrothermal upgrading, etc.^177^ Among the investigated processes, HDO of lignin is the best for the production of aromatics with a return on investment. For HDO of lignin, the authors have considered the solvent-free approach as it avoids the use of extensive solvent recovery and this is one of the positive effects from a techno-economic point of view.^26−28,118,133,146,178^ It should be noted that the quality and type of lignin are important to obtain the desired products. However, organosolv lignin is more expensive than the Kraft/soda lignins. When modeling the process, the authors started from Kraft lignin, assuming a similar yield of alkylphenolics obtained in the lab scale batch experiments. For the HDO of lignin, the capital cost was estimated using the equation derived for the fast pyrolysis system reported by Bridgewater (eq 6).^179^
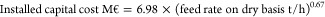
6

**Figure 17:**
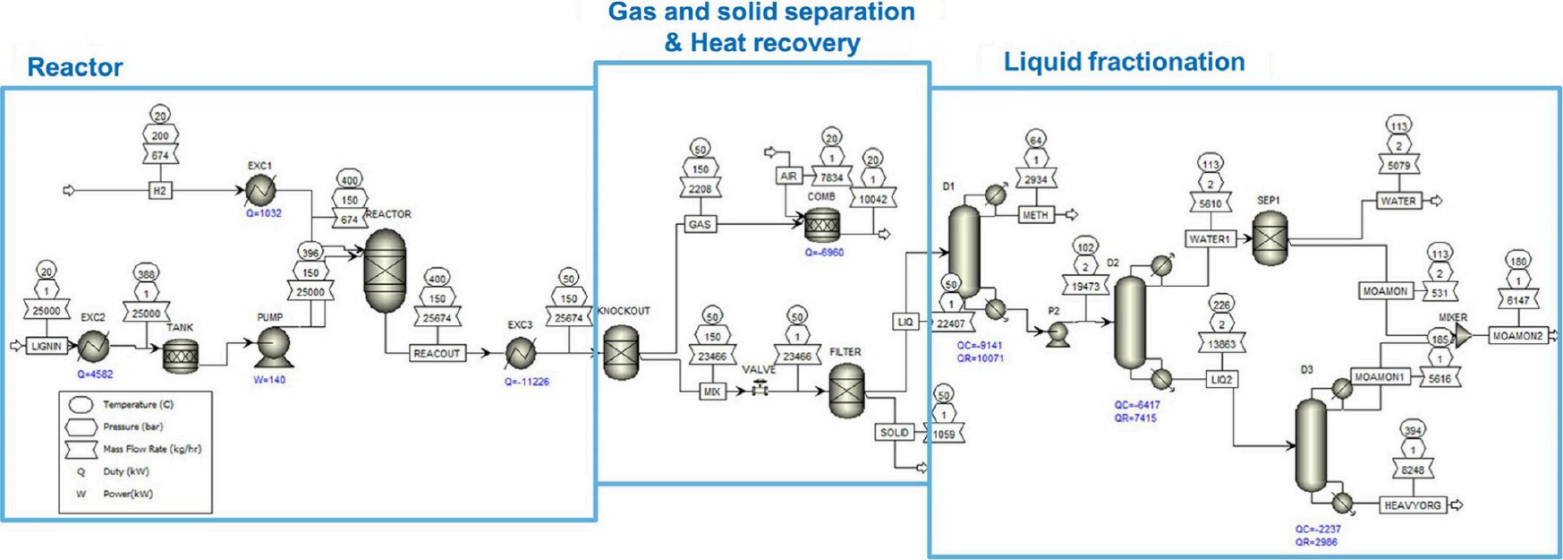
Aspen plus model for the direct hydrodeoxygenation of lignin. Reproduced with permission from ref (177). Copyright 2019 Wiley.

A scaling exponent of 0.65 used for cost estimation in the case of the HDO reactor system and hydrogen, as reported by Jones et al.^180^ For the FCI estimate, the authors chose 2012 as the reference for estimating the FCI for a plant size of 200 kt year^–1^ of lignin input, which is equivalent to 1Mt year^–1^ of lignocellulosic biomass (assuming 20% lignin yield on a dry basis).^181^ For utilities, 2016 prices were considered as prices fluctuated widely in the period 2012–2015. The proposed process model (Aspen Plus) for HDO of lignin consists of three main units namely, the main reactor system, the separation units and the fractionation units, as shown in Figure 17.^177^ In the main reactor, lignin and hydrogen are fed into the reactor at assumed operating conditions of 400 °C and 150 bar H_2_, and the NiMo catalyst was used as a reference. The consumption of hydrogen was assumed to be 300 NL kg^–1^. After the reaction, the products are cooled to 50 °C at the outlet and the pressure is reduced to separate the liquid and solid phases. The gas phase is used for heat generation. The liquid phase consists of an organic and an aqueous phase. The organic phase contains light organic substances (e.g., methanol, acetic acid), mixed oxygenated monoaromatics (e.g., phenol, guaiacol, catechol, syringol, and *m*-cresol) and heavy organic substances (oligomers). The components of the liquid phase are separated in the distillation/fractionation unit. Light organic substances (methanol) and water are separated one after the other in the first and second distillation column. A hydrophobic membrane is used for efficient separation of the organic substances in the water. In the third distillation column, the oxygenated aromatics and the heavy organic substances are separated. The conversion of lignin is 96% and no significant amount of char was formed. The product yield in the HDO is as follows: Gases 8%, light organics 11%, oxygenated monoaromatics 24%, heavy organics 32%, water 20%. The details of mass, energy, utility requirement, capital investment input/outputs, energy balances, external utility requirements and annual revenues, etc. are shown in Table 7. As the feasibility and implementation of this process depends on the production of valuable chemicals, the economic analysis is positive. It is reported that lignin at an input of 25000 kg h^–1^ produces a large amount of valuable chemicals such as light organics (2934 kg h^–1^), oxygenated aromatics (6147 kg h^–1^) and heavy organics (8248 kg h^–1^). The required gross heating and cooling capacity is 26.1 and 29.0 MW, respectively.

**Table 7:** Equipment Cost, Mass, Energy Balances, and Utility Requirements for the HDO of Lignin (Data Obtained from Ref (177))

input (kg h^–1^)		equipment cost (M€)^a^	
lignin	25000	reactor system^a^	47
hydrogen (kg h^–1^)	674	separation system^b^	4
air (kg h^–1^)	7834	fractionation^c^	40
output (kg h^–1^)		indirect cost^d^	27
light organics	2934	contingency^e^	24
oxygenated aromatics	6147	FCI^f^	142
heavy organics	8248	working capital^g^	21
waste water	5079	TCI^h^	163
unconverted lignin	1059	raw material cost (per year)	69
flue gas	10042	utilities (per year)	4
energy balances (MW)		fixed cost (per year)	24
gross heating duty	26.1	annual revenue (M€)	
gross cooling duty	29.0	oxygenated aromatics	71
external utility requirements		light organics	8
electricity (kW)	140	heavy organics	53
cooling water (t h^–1^)	348	ROI	12.1%
natural gas (MW)	1.8	PBP	5 years

a f = sum of (a, b, c, d, e), h = g + f.

The estimated costs for the solids processing reactor, the separator, and the fractionation sections are 47 M€, 4 M€, 40 M€, respectively. Due to the complexity of the separation units, a high investment is required. This includes indirect costs (27 M€), costs for contingencies (24 M€) and working capital of 21 M€. Together with the techno-economic assessment of the HDO of lignin, it shows that a high investment (163 M€) is required as this process uses expensive hydrogen. This amount does not include the operating costs for lignin, hydrogen and operating resources. As the reactor system is designed to process 200kt year^–1^, the calculated costs for lignin are 50 M€ year^–1^ and for hydrogen 14 M€ year^–1^. The total operating costs for the lignin HDO are 97 M€ year^–1^. Although it is a large investment, the production of mixed oxygenated aromatics (alkylphenolics) and heavy organics contributes to a high return on investment. Total revenues amount to 131 M€ year^–1^, with the individual compound classes contributing as follows: mixed oxygenated aromatics, 71 M€ (54% of revenues), heavy organics 53 M€ (40% of revenues) and light organics 8 M€ (6% of revenues). The calculations resulted in a good return on investment of around 12% with a promising payback period of 5 years. The positive NPV (47 M€) demonstrates the feasibility of producing valuable chemicals from lignin through the HDO process.

**Figure 18:**
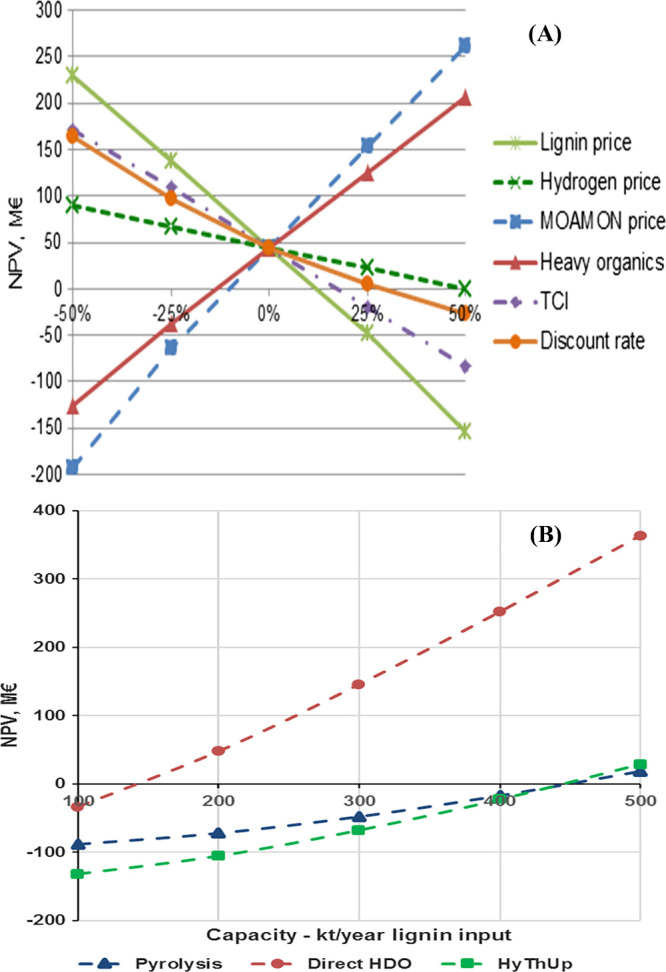
(A) Sensitivity analysis for the HDO of Kraft lignin and (B) sensitivity analysis on variation in lignin input capacity. Reproduced with permission from ref (177). Copyright 2019 Wiley.

The authors performed a sensitivity analysis to assess the impact of fluctuations in raw material and product prices on the economic performance. Since the main sources of revenue in the HDO of lignin are the mixed oxygenated aromatics and the heavy organics. Therefore, the prices of these products have a major impact on economic performance. Figure 18(A) shows the lignin and product prices together with the discount rate. The results show that the cost of hydrogen has a small impact. However, the lignin price has a large influence. Figure 18(A) shows that a positive NPV is obtained for the production of chemicals from Kraft lignin. A reduction in the price of lignin and the TCI leads to a positive NPV. For example, reducing the lignin price by 25%, i.e., 250 M€ ton^–1^, leads to a positive NPV. However, this process is not feasible in the following cases: (i) 20% increase in TCI price, (ii) 10% increase in lignin price, (iii) 15% decrease in mixed oxygenated monoaromatics prices. Apart from this, the lignin feeding capacity of the plant also has a major impact on the feasibility of the process. Figure 18(B) shows the effect of lignin feed capacity on the NPV. Since the current plant is designed for 200 kt year^–1^, this results in a positive NPV (47 M€). In the case of a lower capacity, i.e., about 100 kt year^–1^, the HDO process for lignin has a negative NPV on an economic scale. The HDO process for lignin has a high NPV compared to other processes such as pyrolysis and the hydrothermal upgrading process. This techno-economic assessment demonstrates the feasibility and risks of the HDO process for lignin to biobased aromatics at an early stage and provides guidance for the further development and commercialization of the HDO technology.

## 8 Crude Oil vs Lignin Oil Refining

Similar to crude oil, lignin oil is a mixture of several components. Crude oil consists mainly of long-chain hydrocarbons, aromatics, asphaltenes etc.^182^ On the other hand, the main components of lignin oil are six-membered aromatics, alkanes, oligomers, etc. A comparison between lignin oil and crude oil is shown in Table 8. The heteroatoms in lignin oil are oxygen and sulfur (sulfur containing lignins: Kraft and lignosulfonates), whereas in crude oil the most important heteroatoms are sulfur and nitrogen. The atmospheric and subsequent vacuum distillation of crude oil produces VR (also known as short residue (SR)). VR is converted into fuels by catalytic cracking and, in a further step, heteroatoms such as sulfur and nitrogen are removed from the distillates by a hydrotreatment process. Zeolites are generally used as catalysts for cracking, whereby the choice of zeolite (ZSM/Y etc.) depends on the desired product.^183^ For the hydrotreatment process, Ni-promoted Mo/W based catalysts are used in petroleum refineries. Cracking and HDX (X = S or N) are two different processes in the refinery that are comparable to the depolymerization of lignin. In solvent-free lignin hydrotreatment, cracking and oxygen removal can be performed in a single step. The cracking of lignin requires a catalyst with acidic properties and oxygen removal ability. This could be achieved with Ni/Co promoted Mo/W on acidic supports such as Al_2_O_3_, SiO_2_, SiO_2_–Al_2_O_3_, ZrO_2_ and zeolites, etc., as reported in the literature.^26,97,113^ In addition to these catalysts, other non-noble and noble metal catalysts have also been reported.^27−29,125,158^ However, noble metal catalysts are highly active and the hydrogenation of the benzene ring can lead to the formation of low value products such as cyclic alkanes/alkanes. Moreover, noble metal catalysts are not suitable for sulfur-containing lignins such as Kraft and lignin sulfonates, etc. On the other hand, non-noble metal catalysts show low activity in the depolymerization of lignin.^27,28,158^ In this context, catalysts based on Ni/Co promoted Mo/W are suitable for the removal of oxygen from lignin to produce aromatics, as these catalysts are known for the removal of heteroatoms.^26,122^

**Table 8:** Comparison of Crude Oil Characteristics with Lignin Oil

parameter/characteristic	crude oil	lignin oil
separation	initial distillation followed by secondary catalytic processes	distillation of various components only after the hydrotreatment of lignin
composition	long chain hydrocarbons, aromatics, asphaltenes, heterocyclic compound containing S, N, and O	alkylphenolics, aromatics, cyclic/hydrocarbons, guaiacols, ketones, oligomers
	major: long chain hydrocarbons	major: aromatics including oxygenated
polarity	nonpolar/less polar	highly polar
	(depending upon the source, crude oil contains salts, some polar components like nitrogen, sulfur containing molecules and trace amount of chlorine)	(due to presence of hydroxy, methoxy functional groups)
heteroatoms	N, S, O, etc.	O and S (in the case of sulfur containing lignins)
catalysts	for crude oil, various catalytic systems are used such as Pt/Al_2_O_3_ for reforming, Ni/Co promoted Mo/W catalysts for HDS, HDN, Zeolites for cracking.^184^	Ni/Co promoted Mo/W catalysts for HDO^26,28,113^ or noble metal catalysts^27,31,118,146^
catalyst stability	the petroleum distillates are mainly composed of sulfur as heteroatom; therefore, the HDS catalysts have a long-lifetime.	HDS catalysts deactivate during the reaction, which is due to the presence of oxygen. Therefore, a continuous supply of a sulfidation source (DMDS) is required along with the feed to make the catalyst active.

## 9 Conclusions, Challenges, and Future Perspectives

Catalytic hydrotreatment of lignins under solvent-free conditions is one of the green approaches for the sustainable production of valuable chemicals. In this review article, the lignin structure, different lignin sources and depolymerization routes, the need for a solvent-free approach and recent developments in catalytic hydrotreatment of lignin under solvent-free conditions in batch, semicontinuous and continuous reactors are discussed. Special emphasis is given to the key factors of lignin hydrotreatment, such as the role of the catalyst, the reaction mechanism, and the analytical techniques, etc. This overview shows that in most cases, regardless of the type of lignin, catalyst and reaction conditions, the hydrotreatment of lignins usually produces alkylphenolics as the main product and oxygen-free aromatics as the second main product.

In the solvent-free catalytic hydrotreatment of lignins, the minimum temperature for the liquefaction of lignin to separate aqueous and organic fractions is 400 °C. The most studied catalysts for solvent-free lignin hydrotreatment are metal sulfides and noble catalysts. Compared to the sulfur-free lignins, the technical lignins such as Kraft, lignosulfonate and lignoboost etc. are an attractive source as they are readily available and inexpensive. Another advantage of sulfur-containing lignins is that the sulfur they contain serves as a source of sulfidation to maintain the activity of metal sulfides (such as Ni/Co promoted Mo/W) during the hydrotreatment process. In the case of sulfur-containing lignins, the best reaction temperature for a high yield of monomers and aromatics (oxygen-free and oxygen-containing) is in the range of 400–450 °C. Metal sulfides could also be used for sulfur-free lignins. However, in order to maintain the activity of the catalyst, an additional sulfur source (e.g., DMDS) must be continuously supplied with the feedstock.

The noble metal catalysts are mainly used for sulfur-free lignins (Alcell, Soda, Organocell etc.) and are not suitable for sulfur-containing lignins as the catalyst is quickly deactivated. The most studied and best reaction temperature for the hydrotreatment of sulfur-free lignins over noble metal catalysts is 400 °C. The main disadvantage of noble metal catalysts is ring hydrogenation at higher temperatures, which is undesirable. In addition to metal sulfides and noble metal catalysts, other transition metal catalysts have also been described for the depolymerization of lignin with moderate activity. Supported metal phosphides are also active in lignin hydrotreatment. However, these catalysts need to be tested over a long period of time to verify their stability and activity. The reason for this is that the metal phosphides are unstable in the presence of sulfur sources (H_2_S and sulfur in lignin), which leads to a conversion of the metal phosphides into sulfides (also active in hydrotreatment).

The use of fractionated lignin and/or ball-milled lignin shows better performance than the starting lignin in terms of monomer yield. Thus, knowing the properties of ball-milled and fractionated lignin (bonds such as C–C, C–O, aliphatic, aromatic etc.), one can use a suitable catalyst for efficient depolymerization to produce aromatics. The hydrotreatment of pyrolytic lignins showed a high monomer yield compared to simple lignins. However, since pyrolytic lignins are obtained from pyrolysis oil (this includes pyrolysis of wood, extraction of lignin from pyrolysis oil, and hydrotreatment of pyrolytic lignin), a detailed techno-economic evaluation of this process needs to be carried out. However, the literature shows that the one step approach is more efficient in the production of alkylphenolics than the two-step approach (pyrolysis followed by hydrotreatment). Therefore, the direct solvent-free hydrotreatment of lignin has a positive impact on the techno-economic feasibility, as the alkylphenolics and aromatics are important industrial chemicals and have a higher economic value (>1000€ ton^–1^) than aliphatic fuel components (<600€ ton^–1^). Despite high investments, solvent-free catalytic hydrotreatment of lignins brings high returns compared to other processes such as pyrolysis, hydrothermal upgrading etc. However, if selective oxygen-free aromatics are targeted, other alternative approaches such as mild hydrotreatment of alkylphenolics in a second step might be a better option to control the hydrogenation of the aromatic ring.

### 9.1 Challenges and Future Perspectives

#### 9.1.1 Char Content and Product Yield

One of the biggest challenges in the solvent-free hydrotreatment of lignin is the deactivation of the catalyst and the formation of char. The selection of the catalyst, the type of lignin and the reaction conditions are the most important factors that determine the product yield and production costs. Therefore, the selection of the catalyst is very important, especially the acidity of the catalyst must be adjusted to control the repolymerization of the lignin fragments and the gasification of the products at high temperatures. Zeolites, for example, are known for cracking. Under hydrotreatment conditions, lignin forms oligomers and other lignin fragments. Therefore, the compatibility of the pore size with the depolymerized products is important to avoid the formation of char. The acidity of the catalyst can also be controlled by introducing metal ions such as Na^+^ or Cs^+^ into the zeolite network or by lowering the Al content. The acidity of the catalyst can be controlled by supporting the active components on neutral and mild acidic supports. The formation of gaseous products is unavoidable under hydrotreatment conditions. The main gaseous components observed in solvent-free catalytic hydrotreatment are C_1_–C_3_, CO, CO_2_, etc. The gasification of lignin or lignin products in the solvent-free hydrotreatment could be controlled by tuning the catalyst properties such as acidity, use of various transition metals (oxides, sulfides, carbides, phosphides etc.).

#### 9.1.2 Yield of Oxygen-Free Aromatics

Another challenge in the depolymerization of lignin is the production of oxygen-free aromatics with high yield. As already mentioned, the solvent-free catalytic hydrotreatment process mainly produces alkylphenolics. In order to selectively produce oxygen-free aromatics, the hydrotreatment of lignin could therefore be carried out in two steps, i.e., initial hydrotreatment of lignin and subsequent HDO of alkylphenolics. The reason for this is that the hydrotreating reaction involves high temperatures and pressures, so that the hydrogenation of the aromatic ring cannot be controlled. The HDO reaction, on the other hand, requires mild hydrotreating conditions, i.e., relatively low T and P values. Therefore, the product obtained in the first stage (alkylphenolics) can be used as starting material for the next HDO stage. For example, considering conventional hydrotreatment catalysts, Ni-promoted Mo/W based catalysts could be used for lignin hydrotreatment in the first stage and Co-promoted Mo catalysts for the HDO of alkylphenolics in the later stage. This is comparable to the HDT and HDS units in petroleum refineries.

#### 9.1.3 Future Perspectives for the Production of Oxygen-Free Aromatics

**Figure 19:**
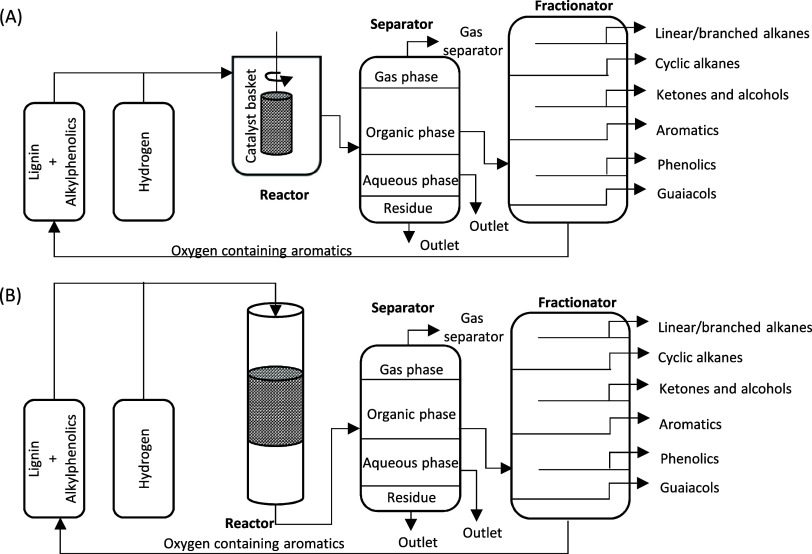
Schematic diagram for the semicontinuous (A) and continuous (B) catalytic hydrotreatment of lignin to valued chemicals.

The main disadvantage of batch reactors is the long contact time, which is why the aromatic rings in the depolymerized products become hydrogenated/saturated, leading to an increase in the yield of low-value chemicals. An alternative to this is the use of semicontinuous reactors. For example, as shown in Figure 19(A), a semicontinuous reactor with a catalyst basket could be used for lignin hydrotreatment. To continuously pump the lignin into the reactor, the lignin must be mixed with alkylphenolics/cresols forming a homogeneous feed (Note: the lignin content in the feed must be optimized to minimize char formation and deactivation of the catalyst). Hydrotreatment of lignin produces a mixture of gas, liquid (consisting of water and lignin oil), residue, etc., which can be separated in a separation unit. The various components of the lignin oil can be separated in the fractionator. In this way, the desired products (oxygen-free aromatics) can be extracted from the fractionator and the remaining oxygenated aromatics can be reused for the production of oxygen-free aromatics. Alternatively, a fixed-bed reactor could be used instead of a semicontinuous reactor, as shown in Figure 19(B). However, the main problem of the fixed bed reactor is the pressure drop due to the formation of solid residues/char during the hydrotreatment of lignin.

Literature shows that conventional metal sulfide catalysts and simple (untreated) lignins are used for single-stage hydrotreatment. The mechanical milling of lignin followed by hydrotreatment had a positive effect on the overall monomer yield. Therefore, the use of ball-milled lignin together with the hierarchical micro/nano- hollow structures of Ni/Co promoted Mo/W based catalysts would be an additional advantage for efficient depolymerization and high yields of oxygen-free aromatics.

In the case of solvent-based hydrotreatment, a protic solvent such as alcohol is used as the hydrogen source. In solvent-free hydrotreatment on the other hand, expensive hydrogen is used. Therefore, it is necessary to look for other alternatives for the hydrogen source. A novel route such as using natural gas (for *in situ* generation of the hydrogen) for lignin hydrotreatment will be beneficial for conventional petroleum and biorefineries. As methane is burnt as flare gas, it can be used instead of hydrogen for the depolymerization of lignin i.e., methane reforming can be combined with the lignin hydrotreatment process. This could generate additional revenue for the petroleum industry and reduce the production costs of lignin hydrotreatment. Alternatively, lignin hydrotreatment technology could be coupled with green electrocatalytic hydrogen, as this research is at an advanced stage and is most cost-effective method of producing hydrogen through water splitting. Utilizing renewable hydrogen and lignin sources for aromatics production could provide additional revenue for the biomass processing industry.

**Figure 20:**
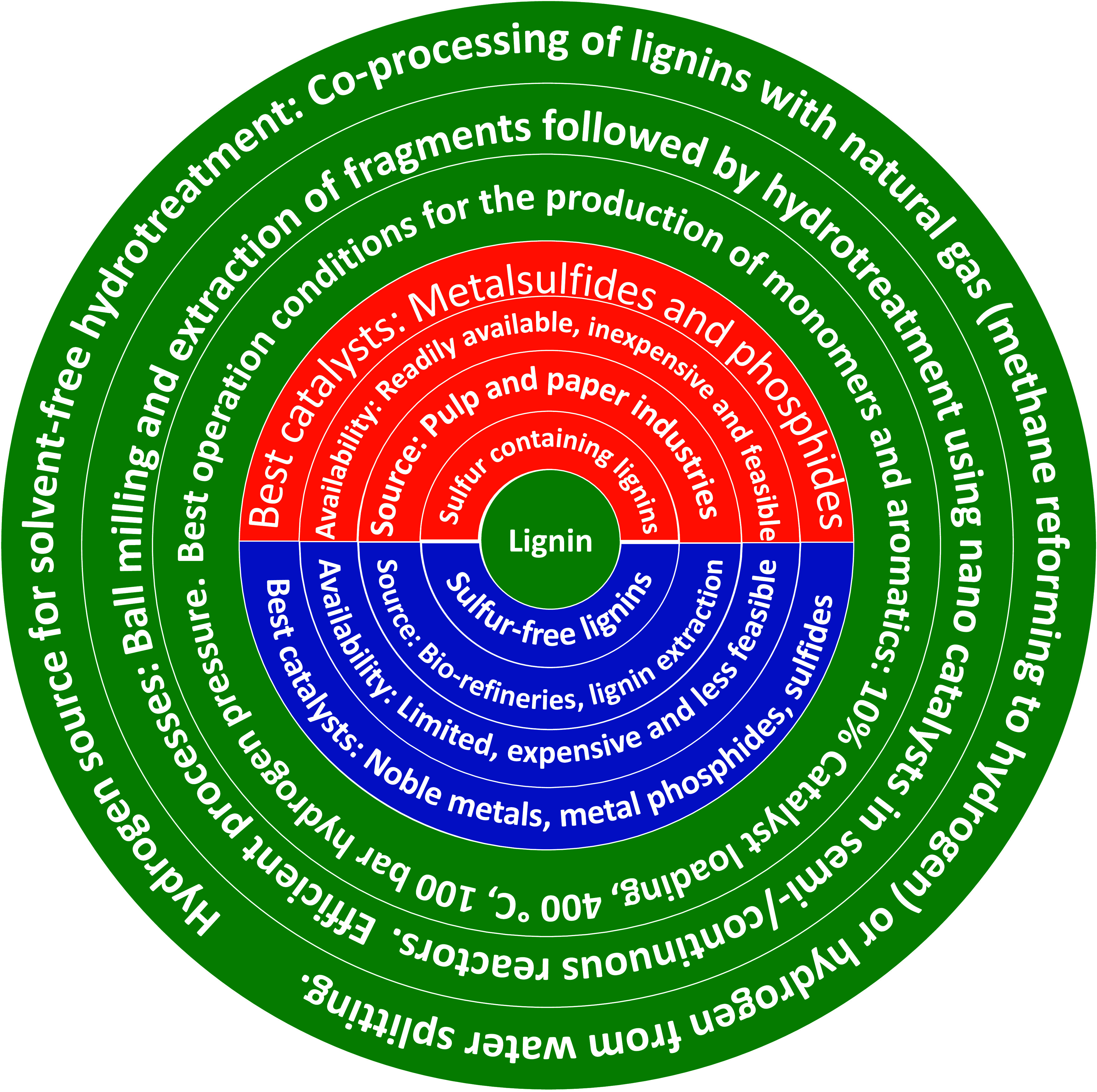
Overall summary and future perspectives of solvent-free hydrotreatment of lignins.

In addition, the gas mixture produced during the hydrotreatment of lignin could contribute to the revenue. This is because the gas mixture consists of H_2_, CO, CO_2_, methane, ethane, ethylene, propane and propylene, etc. With the help of PSA technology, the gas mixture could be separated in to its constituents and hydrogen could be recycled for the hydrotreatment process. These byproducts can be used directly or further processed into valuable chemicals. For example, catalytic hydrogenation of CO, CO_2_ to methanol, ii. C_1_–C_3_ hydrocarbons can be used as fuels or converted into olefins and other valuable chemicals. As mentioned above, lignin could be utilized together with the C1–C3 hydrocarbons (instead of hydrogen). Figure 20 shows the overall summary and future prospects for solvent-free catalytic hydrotreatment of sulfur-containing and sulfur-free lignin.

## Abbreviations

BPE: benzyl phenyl ether
BPS: bioethanol production stillage
BTEX: benzene, toluene, ethylbenzene, and xylene
BTX: benzene, xylene, toluene
CHNS: carbon, hydrogen, nitrogen, sulfur
CP/MAS: cross-polarization/magic angle spinning
DES: deep eutectic solvent
DMDS: dimethyldisulfide
DPE: diphenyl ether
DSPs: dichloromethane soluble products
FCC: fluidized catalytic cracking
FCI: fixed capital investment
FT-IR: Fourier transform infrared
FR: forestry residue
GC–MS/FID: gas chromatography–mass/flame ionization detector
GC–TCD: gas chromatography–thermal conductivity detector
GPC: gel permeation chromatography
GVL: γ-valerolactone
HDN: hydrodenitrification
HDO: hydrodeoxygenation
HDS: hydrodesulfurization
HDT: hydrotreatment
HHV: high heating value
HPTT: high-pressure thermal treatment
HSQC: heteronuclear single quantum coherence
HTL: hydrothermal liquefaction
IL: ionic liquid
LPO: lignin pyrolysis oil
*M*_n_: number-average molecular weight
*M*_w_: weight-average molecular weight
NPV: net present value
PBP: payback period
PCA: principal component analysis
PDI: polydispersity index
PL: pyrolytic lignin
PLA: pyrolysis oil from pine wood
PLB: pyrolysis oil from commercial pine wood
PLC: pyrolysis oil from prunings
PLD: pyrolysis oil from verge grass
PLE: pyrolysis oil from miscanthus
PLF: pyrolysis oil from sunflower seed peel
PSA: pressure swing adsorption
ROI: return on investment
RT: room temperature
SLRP: sequential liquid-lignin recovery and purification
SR: short residue
TCI: total capital investment
THF: tetrahydrofuran
TGA/DTA: thermogravimetric analysis/differential thermal analysis
VGO: vacuum gas oil
VR: vacuum residue
